# Genome-Wide Association Study of Blood Pressure Traits by Hispanic/Latino Background: the Hispanic Community Health Study/Study of Latinos

**DOI:** 10.1038/s41598-017-09019-1

**Published:** 2017-09-04

**Authors:** Tamar Sofer, Quenna Wong, Fernando P. Hartwig, Kent Taylor, Helen R. Warren, Evangelos Evangelou, Claudia P. Cabrera, Daniel Levy, Holly Kramer, Leslie A. Lange, Bernardo L. Horta, COGENT-BP consortium, Jingjing Liang, Thu H. Le, Digna R. Velez Edwards, Bamidele O. Tayo, Kyle J. Gaulton, Jennifer A. Smith, Yingchang Lu, Richard A. Jensen, Guanjie Chen, Lisa R. Yanek, Karen Schwander, Salman M. Tajuddin, Wonji Kim, James Kayima, Colin A. McKenzie, Ervin Fox, Michael A. Nalls, Hunter J. Young, Yan Sun, Jacqueline M. Lane, Sylvia Cechova, Jie Zhou, Hua Tang, Myriam Fornage, Solomon K. Musani, Heming Wang, Juyoung Lee, Adebowale Adeyemo, Albert W. Dreisbach, Terrence Forrester, Pei-Lun Chu, Anne Cappola, Michele K. Evans, Alanna C. Morrison, Lisa W. Martin, Kerri L. Wiggins, Qin Hui, Wei Zhao, Rebecca D. Jackson, Erin B. Ware, Jessica D. Faul, Michael Bray, Joshua C. Danny, Thomas H. Mosley, Walter Palmas, Xiuqing Guo, George J. Papanicolaou, Alan D. Penman, Joseph F. Polak, Kenneth Rice, Eric Boerwinkle, Erwin P. Bottinger, Kiang Liu, Neil Risch, Steven C. Hunt, Charles Kooperberg, Alan B. Zonderman, Cathy C. Laurie, Diane M. Becker, Jianwen Cai, Ruth J. F. Loos, Bruce M. Psaty, David R. Weir, Sharon L. R. Kardia, Donna K Arnett, Sungho Won, Todd L. Edwards, Susan Redline, Richard S. Cooper, D. C. Rao, Charles Rotimi, Aravinda Chadravarti, Xiaofeng Zhu, Kathleen F. Kerr, Alex P. Reiner, Nora Franceschini

**Affiliations:** 1 Department of Biostatistics, University of Washington, Seattle, WA USA; 2 Postgraduate Program in Epidemiology, Federal University of Pelotas, Pelotas, Brazil; 3 Institute for Translational Genomics and Population Sciences, Los Angeles Biomedical Research, Institute and Department of Pediatrics, Harbor-UCLA Medical Center, Torrance, CA USA; 4 William Harvey Research Institute, Barts and The London School of Medicine and Dentistry, Queen Mary University of London, London, UK; 5 National Institute for Health Research Barts Cardiovascular Biomedical Research Unit, Queen Mary University of London, London, UK; 6 Department of Epidemiology and Biostatistics, School of Public Health, Imperial College, London, UK; 7 Department of Hygiene and Epidemiology, University of Ioannina Medical School, Ioannina, Greece; 8 The Framingham Heart Study, Framingham, MA and the Population Sciences Branch, National Heart, Lung, and Blood Institute, National Institutes of Health, Bethesda, MD USA; 9 Departments of Medicine and Public Health Sciences, Loyola University Chicago, Maywood, IL USA; 10 Department of Medicine, University of Colorado Denver, Aurora, CO USA; 11 Division of Public Health Sciences, Fred Hutchinson Cancer Research Center, Seattle, WA USA; 12 Department of Epidemiology, University of North Carolina, Chapel Hill, NC USA; 13 Department of Epidemiology & Biostatistics, School of Medicine, Case Western Reserve University, Cleveland, OH USA; 14 Department of Medicine, Division of Nephrology, University of Virginia, Charlottesville, Virginia USA; 15 Department of Obstetrics and Gynecology, Institute for Medicine and Public Health, Vanderbilt Genetics Institute, Vanderbilt University Medical Center, Nashville, Tennessee USA; 16 Department of Public Health Sciences, Loyola University Chicago Stritch School of Medicine, Maywood, Illinois USA; 17 Department of Pediatrics, University of California San Diego, La Jolla, California USA; 18 Department of Epidemiology, School of Public Health, University of Michigan, Ann Arbor, Michigan USA; 19 The Charles Bronfman Institute for Personalized Medicine, Icahn School of Medicine at Mount Sinai, New York City, New York USA; 20 The Genetics of Obesity and Related Metabolic Traits Program, Ichan School of Medicine at Mount Sinai, New York City, New York USA; 21 Division of Epidemiology, Department of Medicine, Vanderbilt-Ingram Cancer Center, Vanderbilt Epidemiology Center, Vanderbilt University School of Medicine, Nashville, Tennessee USA; 22 Cardiovascular Health Research Unit, Departments of Medicine, Epidemiology & Health Services, University of Washington, and Group Health Research Institute, Group Health Cooperative, Seattle, Washington USA; 23 Center for Research on Genomics and Global Health, National Human Genome Research Institute, National Institutes of Health, Bethesda, Maryland USA; 24 Department of Medicine, Johns Hopkins University School of Medicine, Baltimore, Maryland USA; 25 Division of Biostatistics, School of Medicine, Washington University in St. Louis, St. Louis, Missouri USA; 26 Laboratory of Epidemiology and Population Sciences, National Institute on Aging, National Institutes of Health, Baltimore, Maryland USA; 27 Interdisciplinary Program of Bioinformatics, Seoul National University, Seoul, Republic of Korea; 28 Division of Adult Cardiology, Uganda Heart Institute, Makerere University College of Health Sciences, Kampala, Uganda; 29 Makerere University College of Health Sciences, Kampala, Uganda; 30 Tropical Medicine Research Institute, University of the West Indies, Mona, Jamaica; 31 Department of Medicine, University of Mississippi Medical Center, Jackson, Mississippi USA; 32 Data Tecnica International, Glen Echo, MD USA; 33 Laboratory of Neurogenetics, National Institute on Aging, National Institute of Health, Bethesda, Maryland USA; 34 Department of Epidemiology, Rollins School of Public Health, Emory University, Atlanta, Georgia USA; 35 Center for Human Genetic Research Massachusetts General Hospital, Boston, Massachusetts USA; 36 Anesthesia, Critical Care and Pain Medicine, Massachusetts General Hospital and Harvard Medical School, Boston, Massachusetts USA; 37 Program in Medical and Population Genetics, Broad Institute, Cambridge, Massachusetts USA; 38 Department of Genetics, Stanford University School of Medicine, Stanford, California USA; 39 Division of Epidemiology, School of Public Health, University of Texas Health Science Center at Houston, Houston, TX USA; 40 Department of Medicine, Harvard Medical School, Boston, Massachusetts USA; 41 Division of Structural and Functional Genomics, Center for Genome Science, Korea National Institute of Health, Cheongju, Republic of Korea; 42 Department of Internal Medicine, Graduate Institute of Biomedical and Pharmaceutical Science, College of Medicine, Fu Jen Catholic Univerisity, New Taipei City, Taiwan China; 43 Division of Endocrinology, Diabetes, and Metabolism, Perelman School of Medicine at the University of Pennsylvania, Philadelphia, USA; 44 Human Genetics, Center, School of Public Health, University of Texas Health Science Center, Houston, Texas USA; 45 Cardiovascular Institute, The George Washington University, Washington DC, USA; 46 Department of Internal Medicine, Ohio State University, Columbus, Ohio USA; 47 Survey Research Center, Institute for Social Research, University of Michigan Ann Arbor, Michigan, USA; 48 Department of Biomedical Informatics, Department of Medicine, Vanderbilt University Medical Center, Nashville, Tennessee USA; 49 Department of Medicine, Columbia University, New York City, New York USA; 50 Medical Genetics Institute, Cedars-Sinai Medical Center, Los Angeles, CA 90048 USA; 51 Division of Cardiovascular Sciences, National Heart, Lung, and Blood Institute, National Institutes of Health, Bethesda, Maryland USA; 52 Tufts Medical Center, Tufts University School of Medicine, Boston, Massachusetts USA; 53 Institute for Human Genetics, University of California, San Francisco, California USA; 54 Department of Preventive Medicine, Northwestern University Medical School, Chicago, Illinois, USA; 55 Cardiovascular Genetics, University of Utah, Salt Lake City, Utah USA; 56 Department of Biostatistics, Gillings School of Global Public Health, University of North Carolina, Chapel Hill, NC USA; 57 The Mindich Child Health and Development Institute, Ichan School of Medicine at Mount Sinai, New York City, New York USA; 58 University of Kentucky, College of Public Health, Lexington, KY USA; 59 Department of Public Health Science, Seoul National University, Seoul, Republic of Korea; 60 Division of Epidemiology, Department of Medicine, Institute of Medicine and Public Health, Vanderbilt Genetics Institute, Vanderbilit University Medical Center, Nashville, Tennessee USA; 61 McKusick-Nathans Institute of Genetic Medicine, Johns Hopkins University School of Medicine, Baltimore, Maryland USA

## Abstract

Hypertension prevalence varies between ethnic groups, possibly due to differences in genetic, environmental, and cultural determinants. Hispanic/Latino Americans are a diverse and understudied population. We performed a genome-wide association study (GWAS) of blood pressure (BP) traits in 12,278 participants from the Hispanics Community Health Study/Study of Latinos (HCHS/SOL). In the discovery phase we identified eight previously unreported BP loci. In the replication stage, we tested these loci in the 1982 Pelotas Birth Cohort Study of admixed Southern Brazilians, the COGENT-BP study of African descent, women of European descent from the Women Health Initiative (WHI), and a sample of European descent from the UK Biobank. No loci met the Bonferroni-adjusted level of statistical significance (0.0024). Two loci had marginal evidence of replication: rs78701042 (*NGF*) with diastolic BP (*P* = 0.008 in the 1982 Pelotas Birth Cohort Study), and rs7315692 (*SLC5A8*) with systolic BP (*P* = 0.007 in European ancestry replication). We investigated whether previously reported loci associated with BP in studies of European, African, and Asian ancestry generalize to Hispanics/Latinos. Overall, 26% of the known associations in studies of individuals of European and Chinese ancestries generalized, while only a single association previously discovered in a people of African descent generalized.

## Introduction

Hypertension affects approximately one-third of adults in the United States (US) and is a major risk factor for cardiovascular disease (CVD) morbidity and mortality^1–3^. Blood pressure (BP) is a complex, polygenic trait^4, 5^. Prior genome-wide association studies (GWASs) have identified hundreds of genetic variants associated with BP traits (systolic and diastolic BP [SBP and DBP], pulse pressure [PP], mean arterial pressure [MAP], and hypertension [HT]) in individuals of European^6–10^, East Asian^11^, and African descent^12, 13^, or using trans-ethnic approaches^14–17^. Hispanics/Latinos are the largest minority ethnic group in the US, yet the genetic determinants of hypertension in this population remain poorly examined. In particular, only four genome-wide scan of BP traits to date have included Hispanics/Latinos, and these studies interrogated a limited number of single nucleotide polymorphisms (SNPs) from the Metabochip array^18^, or had very small number of Hispanics/Latinos^15–17^.

Hispanics/Latinos are likely to have more undiagnosed, untreated, or uncontrolled hypertension than other ethnic groups^19, 20^. Most studies of hypertension prevalence among US Hispanics have focused on adults with Mexican background (from the National Health and Nutrition Examination Survey [NHANES]), while studies that included diverse representation of Hispanics/Latinos have shown a marked heterogeneity in the prevalence of hypertension based on Hispanic/Latino background^19, 21, 22^. In the Hispanic Community Health Study/Study of Latinos (HCHS/SOL), the overall age-adjusted prevalence of hypertension was 25.5%, but prevalence ranged from as low as 17% in South American women to 34% in Dominican men^19^.

Hispanics/Latinos in the US have varying degrees of Amerindian, European, and African ancestry. We previously have described the genetic diversity among HCHS/SOL participants based on their country of origin and genetic ancestry^23^. The HCHS/SOL comprises of 12,278 ethnically diverse US individuals, classified into two subgroups: Mainland (individuals with Mexican, Central American, and South American background, and a relatively large proportion of Amerindian ancestry) and Caribbean (individuals with Cuban, Dominican, and Puerto Rican background, and a relatively large proportion of African ancestry). We performed a GWAS of BP traits in the HCHS/SOL, in the Mainland and Caribbean groups separately and combined, with the goal of studying genetic diversity within Hispanics/Latinos with respect to BP traits, and to discover novel BP loci.

## Results

Table 1 shows the characteristics of 12,278 Hispanic-/Latino- Americans from the HCHS/SOL, which included 6,722 Mainland and 5,556 Caribbean individuals. The mean age was 46 years and 59% were female. The prevalence of hypertension was 28% overall, but substantially higher among Caribbean than Mainland subgroups (35% vs. 22%).

**Table 1:** Characteristics of HCHS/SOL study participants 2008–2011, in Mainland and Caribbean groups, and Overall.

	Overall	Mainland	Caribbean
n	12278	6722	5556
Mean age (SD)	46 (14)	45 (14)	48 (14)
female sex	7259 (59.1%)	4062 (60.4%)	3197 (57.5%)
Mean BMI (SD)	30 (6)	30 (5.8)	30 (6.3)
Hypertension	3445 (28.1%)	1476 (22%)	1969 (35.4%)
Mean SBP (SD)	125 (20.1)	122 (19.1)	128 (20.8)
Mean DBP (SD)	75 (11.9)	73 (11.3)	78 (12.1)
Mean MAP (SD)	92 (13.8)	89 (13)	94 (14.1)
Mean PP (SD)	50 (13.4)	49 (12.8)	51 (14.1)

Means and standard deviations (SD) of the continuous BP traits were calculated after adjustment for using hypertensive medication.

**Figure 1:**
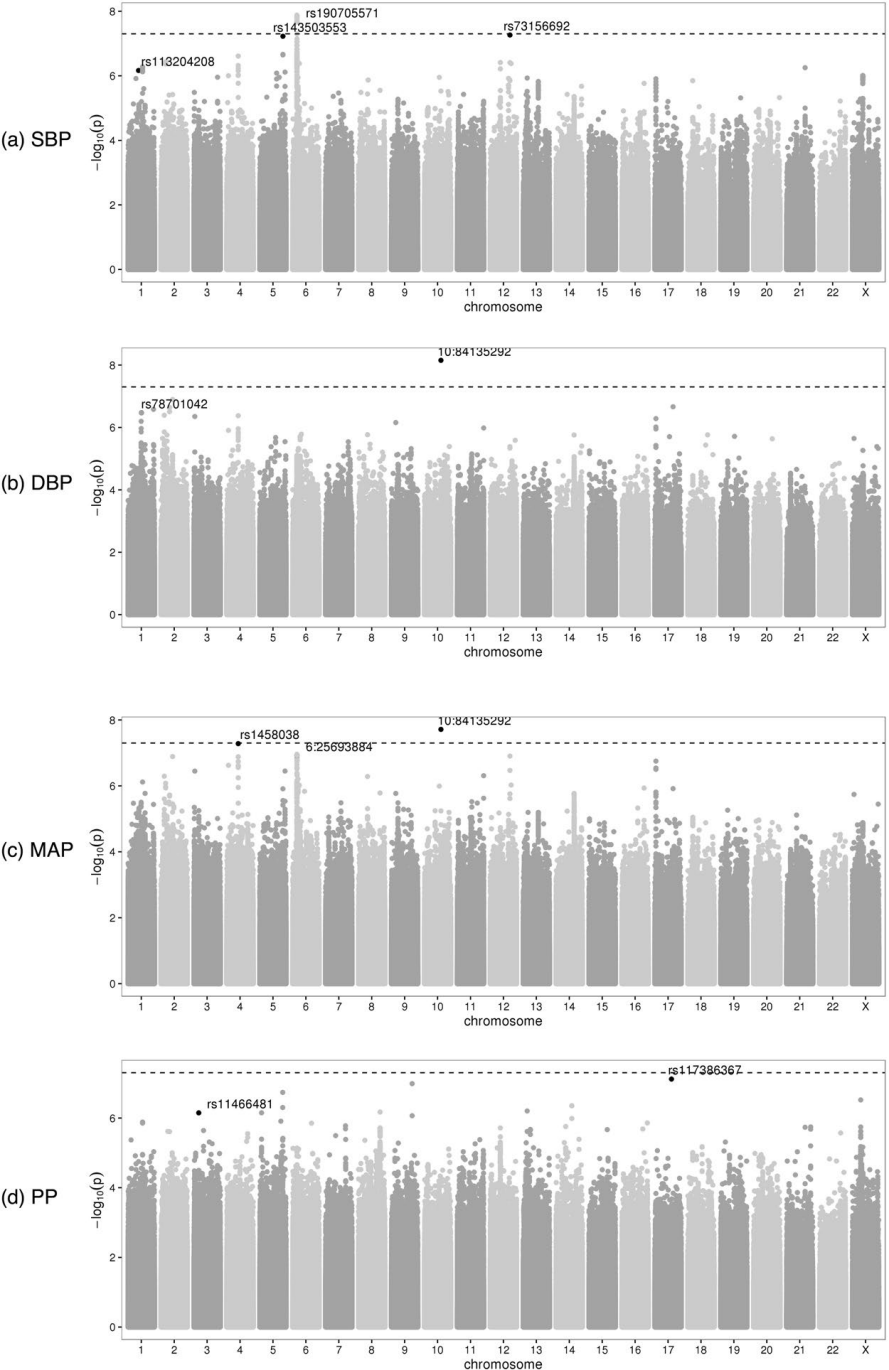
Manhattan plots from the combined analyses of all HCHS/SOL study individuals, for the four quantitative BP traits. For each of the available variants with MAF ≥ 0.01 and imputation quality oevar ≥0.3, a Manhattan plot provides its −log_10_(*P*) against its genomic position. The top SNPs of interest described Tables 2 and 3 are highlighted.

GWAS genomic inflation factors ranged from 1.006 to 1.034 across the GWAS of the 5 BP traits and three subgroups (Mainland, Caribbean, and combined), indicating minimal population stratification. Manhattan and QQ plots for the 4 quantitative BP traits and hypertension across all analyses (Mainland, Caribbean, and combined) are provided in Fig. 1 (quantitative traits in the combined cohort) and in the Supplementary Information. All analyses excluded SNPs with low minor allele frequency (MAF) < 0.01 and imputation quality score < 0.3. There were no associations with *P* < 1 × 10^−7^ in the HT analyses. We provide information about associations detected in the combined, Mainland, and Caribbean analyses below. Accompanying LocusZoom plots portraying the LD structure in these association regions and across the three subgroups, and forest plots comparing effect sizes are provided in Figures S16–S26 in the Supplementary Information. In addition, Tables S1 and S2 in the Supplementary Information provide summary of association analyses for these variants in all BP trait analyses.

### Association testing with quantitative BP traits in the combined discovery sample

Table 2 provides the lead SNPs from each of the regions reaching the genome-wide significance threshold (*P* < 5 × 10^−8^), or the suggestive significance level (*P* < 1 × 10^−7^) in the combined discovery sample. Two common variants reached genome-wide significance (Table 2). The minor allele of a 1 bp indel intronic to *NRG3* located at the genomic region 10q23.1 (MAF = 0.30) was associated with higher DBP (*P* = 7.05 × 10^−9^) and MAP (*P* = 1.93 × 10^−8^). An intronic variant rs190705571 of *SCGN* at the genomic region 6p25.7 (MAF = 0.35) was associated with higher SBP (*P* = 2.16 × 10^−8^). The same variant was also associated with higher MAP, although with a suggestive *p*-value (*P* = 2.29 × 10^−7^). The allele frequencies of the *SCGN* rs190705571 variant differed considerably between Amerindian and African or European ancestral populations (Table S3 in the Supplementary Information). The minor allele of rs190705571 is more common among the Mainland group (MAF = 0.43) compared to the Caribbean group (MAF = 0.25). The associations of both SBP and MAP with rs190705571 in the combined groups were driven primarily by the Mainland group (*β* = 1.65, *P* = 3.93 × 10^−9^, compared to the Caribbean group *β* = 0.56, *P* = 0.14). However, there was no significant evidence for heterogeneity between the Mainland and the Caribbean groups (p-value for heterogeneity = 0.21). Therefore, the Mainland group results are due to both higher MAF and larger sample size.

**Table 2:** Main association results for BP traits in the overall HCHS/SOL discovery sample.

Trait	rsID	Chr	position	A1	A2	type	EAF	beta	SE	p-value	heterogeneity p-value	Gene
MAP	rs1458038	4	81164723	T	C	g	0.24	0.97	0.178	5.22E-08	0.84	*FGF*5
SBP	rs143503553	5	159593663	G	C	i	0.01	7.994	1.475	5.94E-08	0.26	
SBP	rs190705571	6	25693887	T	G	i	0.65	1.268	0.226	2.16E-08	0.21	*SCGN*
MAP	rs190705571	6	25693887	T	G	i	0.65	0.827	0.16	2.29E-07	0.19	*SCGN*
MAP		10	84135292	CA	C	i	0.3	1.031	0.184	1.93E-08	0.59	*NRG*3
DBP		10	84135292	CA	C	i	0.3	0.938	0.162	7.05E-09	0.54	*NRG*3
SBP	rs73156692	12	101608695	A	G	i	0.16	1.646	0.303	5.44E-08	0.37	*SLC*5*A*8
PP	rs117386367	17	53098512	A	G	i	0.01	5.006	0.931	7.61E-08	0.11	

For each locus associated with a BP trait we provide the lead SNP. The effect size, beta, is of the effect allele A1. EAF is the frequency of A1 in the overall sample. Imputation “type” is either ‘i’ (imputed) or ‘g’ (genotyped). The effect estimates, standard errors (SEs) and heterogeneity test p-values were obtained from a fixed-effects meta-analysis across the genetic analysis groups.

Four additional variants had suggestive evidence of association with BP (Table 2). Two of these variants were common (MAF ≥ 0.05) and two were low frequency (MAF ≈ 0.01). The minor allele of rs117386367 (MAF = 0.01) on chromosome 17 was associated with higher PP (*P* = 7.61 × 10^−8^), the minor allele of rs73156692 (MAF = 0.16) located 5 kb 5′ of *SLC*5*A*8 on chromosome 12 was associated with higher MAP (*P* = 5.44 × 10^−8^), the minor allele of rs143503553 (MAF = 0.01) on chromosome 5 was associated with higher SBP (*P* = 5.94 × 10^−8^), and the minor allele of rs1458038 (MAF = 0.24) on chromosome 4 (*FGF*5) was associated with higher MAP (*P* = 5.22 × 10^−8^). rs1458038 is a known association variant for BP traits^8^. There were no significant differences in MAF or evidence of heterogeneity of effect for any of the variants among Mainland and Caribbean groups (Table 2) except for rs73156692, which had slightly higher MAF among Caribbean individuals (MAF = 0.19, compared to Mainland MAF = 0.13). However, there was no significant evidence of heterogeneity between Mainland and Caribbean Hispanic/Latino subgroups for this SNP.

### Mainland- and Caribbean-specific associations

The GWAS restricted to the Caribbean and Mainland subgroups of the HCHS/SOL identified three genome-wide significant variants associated with BP traits, all for low frequency variants (MAF of 0.01) in the Caribbean group (Table 3). rs11466481, an intronic variant to *TGFBR*2 on chromosome 3 was associated with PP; rs78701042, an intronic variant to *NGF* on chromosome 1, was associated with DBP; and rs113204208, an intergenic variant on chromosome 1, was associated with SBP. However, these variants were not significantly associated with the corresponding traits in the Mainland subgroup (all p-value > 0.2). Nonetheless, the estimated directions of these variant associations in the Mainland group were consistent with those in the Caribbean group. Therefore, it is possible that these association were not detected in the Mainland group and in the combined cohort due to lack of power. Specifically, even if the effect size in the Caribbean group is the true effect size, given the frequencies of the variants the powers to detect these associations in the combined group (with *p*-value < 5 × 10^−^^8^) are <0.1, and the powers to detect these associations in the Mainland group with *p*-value < 0.05 are 0.4–0.6). The *NGF* variant rs78701042 is in the same region as an unvalidated variant rs11102916 reported by ref. 17. In conditional analysis adjusted for rs11102916, the DBP association of our *NGF* variant remained genome-wide significant in the Caribbean group. Also note that the previously-reported rs11102916 was only marginally associated with DBP in the HCHS/SOL (*p*-value = 0.051 in the combined cohort).

**Table 3:** Association results in analyses stratified by Mainland and Caribbean subgroups.

Trait	rsID	Chr	position	A1	A2	type	Caribbean				Mainland				overall	heterogeneity
							EAF	beta	SE	p-value	EAF	beta	SE	p-value	p-value	p-value
SBP	rs113204208	1	90549106	G	C	i	0.01	7.919	1.416	2.23E-08	0.01	1.363	2.037	5.03E-01	6.83E-07	<0.001
DBP	rs78701042	1	115841602	T	C	i	0.01	5.443	0.957	1.31E-08	<0.005	0.035	1.927	9.85E-01	3.40E-07	0.03
PP	rs11466481	3	30664148	T	C	i	0.04	3.182	0.568	2.08E-08	0.01	0.336	0.916	7.13E-01	7.10E-07	<0.001

For each locus associated with a BP trait in one of the Mainland or Caribbean subgroups we provide the lead SNP. Imputation “type” is either ‘i’ (imputed) or ‘g’ (genotyped). The effect size, beta, is of the effect allele A1. EAF is the frequency of A1 in the appropriate subsample. The effect estimates, standard errors (SEs) and heterogeneity p-values were obtained from a fixed-effects meta-analysis across the genetic analysis groups.

### Replication of newly discovered loci in independent samples

Table 4 reports association testing results for leading variants from the loci that were identified in the HCHS/SOL in three independent data sets of admixed Southern Brazilians (the 1982 Pelotas Birth Cohort Study, *n* = 2,764), African American (COGENT-BP consortium *n* = 22,000–32,000), and European ancestry (WHI, *n* = 14,900–17,200, and UK Biobank, *n* = 140,886). Results for 6 of the lead SNPs and traits were available in the 1982 Pelotas Birth Cohort Study, 4 lead SNPs were available in COGENT-BP, and 4 (different) lead SNPs were available in the European ancestry follow-up. Lead SNPs were not available when they were monomorphic in African or European populations. MAP data was not available in COGENT-BP and UK Biobank, so we also examined the association of an MAP variant of *SCGN* with SBP, since it was also near-significant for this trait in our discovery sample. Overall, we corrected for 21 hypothesis tests for replication testing, leading to significance threshold of 0.0024. Of the 11 variants in 8 regions examined in replication, 4 were proxies, i.e not the lead HCHS/SOL variants in their region.

**Table 4:** Association testing results in follow-up studies.

trait	rsID	Chr	position	A1	HCHS/SOL			COGENT			Pelotas			EA meta	
					EAF	beta	*p*-value	EAF	beta	*p*-value	EAF	beta	*p*-value	beta	*p*-value
SBP	rs113204208	1	90549106	G	0.01	5.78	6.83E-07	0.06	−0.48	2.28E-01	0.01	−4.28	3.20E-02		
DBP	rs78701042	1	115841602	T	0.01	4.38	3.40E-07	0.04	0.28	3.35E-01	0.01	4.24	8.56E-03		
PP	rs11466481	3	30664148	T	0.02	2.4	7.10E-07	0.15	−0.04	8.49E-01	0.03	−0.16	8.15E-01		
SBP	rs143503553	5	159593663	G	0.01	7.99	5.94E-08				0.01	0.8	7.41E-01	0.42	3.70E-01
SBP	rs9366626*	6	25684953	G	0.55	1.18	8.75E-08	0.7	0.14	5.00E-01	0.6	−0.5	1.12E-01	−0.16	1.50E-02
MAP	rs9366626*	6	25684953	G	0.55	0.79	3.04E-07				0.6	−0.29	2.56E-01	−0.31	2.25E-02
DBP		10	84135292	CA	0.3	0.94	7.05E-09							0.06	3.15E-01
MAP	rs7909484*	10	84206002	T	0.4	0.68	1.50E-05							0.15	3.37E-01
DBP	rs7909484*	10	84206002	T	0.4	0.6	1.55E-05							0.01	8.97E-01
SBP	rs73156692	12	101608695	A	0.16	1.65	5.44E-08	0.16	0.11	6.85E-01	0.2	−0.53	1.71E-01	0.21	7.05E-03
PP	rs117386367	17	53098512	A	0.01	5.01	7.61E-08				0.01	2.38	6.76E-02	−0.1	8.05E-01

For each locus reported in Tables 2 and 3 (associated with a BP trait in either the overall sample, or one of the Mainland or Caribbean groups), and available in the follow-up studies, we provide effect allele frequency (EAF), estimated effect size (beta), and p-value in both the HCHS/SOL (from analysis in the overall sample) and the follow-up cohorts. *In a few instances, we report available proxy SNP rather than the lead SNPs, or more weakly associated trait.

Using the 0.0025 significant replication threshold, none of the associations replicated. However, a few loci had suggestive evidence for replication: rs78701042 association with DBP had p-value 0.0086 in the 1982 Pelotas Birth Cohort Study and a similar effect size to the HCHS/SOL (HCHS/SOL *β* = 4.38, PELOTAS *β* = 4.24). This variant is more common in African Americans (MAF = 0.04 in COGENT-BP), yet the estimated effect size in COGENT was 0.28 and the p-value was 0.33. In addition, the SBP locus rs73156692 had *p*-value = 0.007 in the European ancestry replication results. Note that this variant has similar estimated effect directions and sizes in all replication studies (between 0.16 to 0.21). Other loci were nominally associated with BP (*P* = 0.01–0.07), but some of these associations had different directions of effect between discovery and replication studies.

### Generalization of previously reported associations to the HCHS/SOL

To assess the generalizability of previously identified BP loci to HCHS/SOL Hispanics/Latinos, we tested previously reported associations using a directional False Discovery Rate (FDR)-based generalization testing procedure. A comprehensive table with results is provided in the Supplementary Information. We here report a summary of these results that account for most prior published BP GWAS papers, excluding those published in 2017. That is, although we performed and report results from generalization testing using^17^ results (3 generalized associations), these are not used in the summary presented here.

Based on 314 SNP-trait associations, involving 178 unique SNPs in 114 distinct genomic regions of 1 MB around a SNP reported in blood pressure GWAS^6–9, 11–14, 16^ in populations of European, Chinese, and African ancestries, and in a trans-ancestry analysis. Overall, 58 (18%) associations generalized to one of the HCHS/SOL groups (Mainland/Caribbean, or combined). Of the 44 associations reported in studies of African ancestry, only 1 association generalized to Hispanics/Latinos. Of the 57 associations reported in Chinese ancestry studies, 15 (26%) generalized to Hispanics/Latinos. Of the 36 associations reported in trans-ancestry analyses 4 generalized to the HCHS/SOL, of these, 2 associations were also reported in European ancestry studies. Finally, of the 188 associations reported in studies of European ancestry, 41 (22%) associations generalized.

Most of the associations that generalized in the Caribbean and in the Mainland groups, also generalized in the combined analysis. There are five exceptions. First, rs1173771 (*NPR3-C5orf23*) and rs13359291 (*PRDM6*), both on chromosome 5, and rs1378942 (chromosome 15, *CYP1A1-ULK3*) generalized in Mainland group but not in the combined (or Caribbean group) analysis. These associations were all reported in studies of Europeans. rs1378942 was additionally reported in a trans-ancestry analysis which was potentially driven by a large European sample. The directions of estimated associations of these SNPs were the same in the discovery studies and in the Mainland and the Caribbean subgroups but with attenuated estimates in the Caribbean group. This is possibly due to the lower proportion of European admixture in the Caribbean group, compared to the Mainland group^23^. Second, two PP-association variants rs7255 (chromosome 2) and rs57448815 (chromosome 21) reported in trans-ethnic analyses generalized only in the Caribbean group. These variants also had the same directions of associations in the Mainland group, but with smaller effect size.

**Figure 2:**
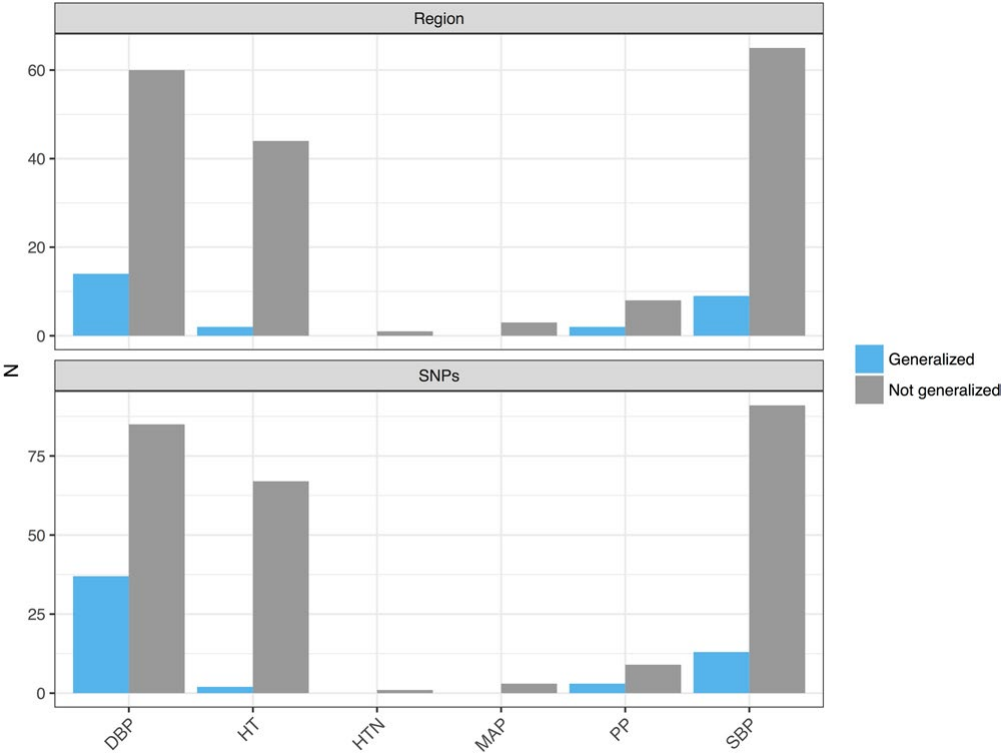
Generalization of BP association SNPs and regions in the HCHS/SOL participants (n = 12,278). For each of the traits investigated in the HCHS/SOL, the figure provides the counts of SNP-trait associations that generalized, and did not generalize to the HCHS/SOL Hispanics/Latinos.

In general, a very low proportion of the variants investigated for hypertension and SBP (Fig. 2) generalized across these populations (hypertension: 2 of 69 interrogated SNPs, or 2 SNPs from 46 regions (4%), SBP: 14 of 105 (13%), or 10 out of 75 (13%) of regions, while a high proportion of DBP SNP associations generalized: 39 of 125 SNPs (31%) corresponding to 16 of 76 (21%) regions.

## Discussion

This is the largest GWAS of BP traits conducted using high density imputed and genotyped SNPs in Hispanics/Latinos, a culturally and genetically diverse population comprised of many subgroups. Our main findings are (1) the identification of two potential novel loci for SBP and DBP at *SCGN* and *NRG3*, (2) the identification of three potential loci in the stratum of Caribbean subgroup, which showed heterogeneity of effects across the Mainland and Caribbean subgroups, and (3) the validation of several established loci for BP traits identified in GWAS of European, African and East Asian ancestries. However, none of the newly discovered BP loci in HCHS/SOL replicated when accounting for multiple testing the four independent samples of admixed Southern Brazilian, African American, or European American ethnicity.

We tested multiple traits, and multiple subsets of the data set (Caribbean, Mainland, and combined), and also investigated variants with MAF at least 1%, while the conventional 5 × 10^−8^ genome-wide significance threshold was developed based on testing common variants^24^. We attempted to replicate all potential novel findings, as it is less likely that a handful of false discoveries will be replicated or generalized in an independent study^25^. It is possible that associations did not replicate because the findings are false positives. However, non-replication could also be due to low power. Under the (unlikely) assumption that the variants reported in Table 4 are causal in all populations and have the same effect size as estimated using the combined cohort, and that BP traits have similar variance in all study populations, the combined cohort of European ancestry had power >0.99 to detect all associations in the available variants, the COGENT-BP consortium had power >0.9 for most associations (0.58 for one), while the 1982 Pelotas birth cohort study had power <0.1 for all associations. However, these power calculations do not account for the “winner’s curse”, and true effects are probably smaller than the effects estimated from the discovery GWAS^26^. Moreover, it is likely that the detected variants are not in fact the causal variants, but rather tag them. Due to differing genetic architectures, we expect that optimal tag variants may differ among populations. Since the estimated effect at a tag SNPs is related to the effect of the true causal SNP via the LD between them, the same tag SNP may have different association with the trait in different populations, suggesting that the associations of the interrogated SNPs may be lower in the replication populations compared to the HCHS/SOL.

One of the newly identified BP loci in the overall sample is located in a region of high LD on chromosome 6p22 intronic to *SCGN*. The association signal spans about 75 kb and extends into a region containing the large histone gene cluster and a family of sodium-dependent phosphate and urate transporter genes (*SLC*17*A*1, *SLC*17*A*3, and *SLC*17*A*4). The sodium/phosphate cotransporter *NPT*1 (*SLC*17*A*1; MIM 182308) is located in the renal proximal tubule and regulates renal phosphate excretion. *SLC*17*A*4 is a similar sodium/phosphate cotransporter in the intestinal mucosa that plays an important role in the absorption of phosphate from the intestine^27^. The Hispanic BP variant is also located about 500 kb from the HFE BP GWAS locus previously reported in Europeans. HFE encodes the protein associated with hemochromatosis. Several LD proxies for the SCGN intronic index SNP are located within intestinal and liver promoter regions and are cis-eQTLs for SLC17A3, SLC17A4, TRIM38, an E3 ubiquitin ligase reported to regulate signaling in innate immune and inflammatory responses. The extended 6p22 region also contains a number of GWAS signals for iron and red blood count traits and serum uric acid levels.

In generalization analysis, we investigated and summarized more than 300 previously reported SNP associations with BP traits, corresponding to 115 genomic regions, and also close to 500 additional associations that were mostly not replicated or validated before, which we report in the Supplementary Information. We say that a region generalizes if at least one SNP-BP trait association in the region generalizes. While about 18% of the associations and regions generalized to Hispanics/Latinos, most of the generalized associations are DBP loci. Interestingly about the same proportion of associations generalized from Chinese (26%) and European (22%) ancestry studies to Hispanics/Latinos, while only a single association reported in an African ancestry study generalized to Hispanics/Latinos. The slightly lower percentage of generalized associations from Europeans compared to Asians is likely due to the recent GWAS studies with very large sample sizes, detecting small effect sizes that the HCHS/SOL is not powered to detect. In fact, the power for detecting associations reported in the large recent GWAS^9, 16^ was no higher then 0.34 and usually lower than 0.2 for all associations, when using the liberal *α* level 0.05. When requiring correction for multiple testing (as needed), power for all associations is close to 0. Considering all studies used in generalization analyses, there were only 3 associations with power larger than 0.8 at the liberal 0.05 *α* level. Two of them were reported in Chinese and did in fact generalize, the third was reported in a population of European ancestry and did not generalize. While these analyses were done under the assumption that the estimated effect sizes in the previously reported SNPs are the true ones, and are the same in the discovery populations and in the HCHS/SOL, these assumptions likely do not hold. Interestingly, for 33% of the SNPs, the effect size estimated in HCHS/SOL was larger than the one observed in the previous studies, while usually we would expect to see lower effect sizes in a follow-up study compared to a discovery study. Future whole genome sequencing studies will help unveil the underlying genetic architecture of these traits and association loci.

## Methods

### HCHS/SOL Population

The HCHS/SOL is a community-based cohort study of 16,415 self-identified Hispanic/Latino persons aged 18–74 years selected from households in predefined census-block groups from four US field centers (Chicago, Miami, the Bronx, and San Diego). The census-block groups were chosen to provide diversity with regard to socioeconomic status and national origin or background. Participants self-identified as having a Hispanic/Latino background; the largest groups were Central American, Cuban Dominican, Mexican, Puerto Rican, and South American. The sample design and cohort selection have been previously described^28^. HCHS/SOL participants were recruited between 2008 and 2011 and underwent a baseline clinical examination^29^ including biological, behavioral, and sociodemographic assessments. The study was approved by the institutional review boards at each field center, where all subjects gave written informed consent. All analyses were in accordance with the relevant guidelines and regulations.

### Genotyping and Quality Control in HCHS/SOL

Consenting HCHS/SOL participants were genotyped at Illumina on the HCHS/SOL custom 15041502 B3 array. The custom array comprised the Illumina Omni 2.5 M array (HumanOmni2.5–8v.1-1) ancestry-informative markers, known GWAS hits and drug absorption, distribution, metabolism, and excretion (ADME) markers, and additional custom content including ~150,000 SNPs selected from the CLM (Colombian in Medellin, Colombia), MXL (Mexican Ancestry in Los Angeles, California), and PUR (Puerto Rican in Puerto Rico) samples in the 1000 Genomes phase 1 data to capture a greater amount of Amerindian genetic variation^30^.

We applied standardized quality-assurance and quality-control (QA/QC) methods^31^ to generate recommended SNP- and sample-level quality filters. Samples were checked for sex discrepancies, gross chromosomal anomalies, relatedness and population structure, missing call rates, batch effects, and duplicate-sample discordance. SNPs were checked for Hardy-Weinberg equilibrium, minor allele frequency (MAF), duplicate-probe discordance, Mendelian errors, and missing call rate. A total of 12,803 unique study participants passed QC and met specific clinical inclusion criteria. A total of 2,232,944 SNPs passed filters for both quality and informativeness (polymorphic and unduplicated) and were carried forward for imputation and downstream association analyses.

### Imputation in the HCHS/SOL

Genome-wide imputation was carried out with the full, cosmopolitan 1000 Genomes Project phase 1 reference panel (n = 1,092)^32^. The HCHS/SOL samples were imputed together with genotyped SNPs passing the quality filter and representing unique genomic positions on the autosomes and non-pseudoautosomal portion of the X chromosome. Genotypes were first pre-phased with SHAPEIT2 (v.2.r644) and then imputed with IMPUTE2 (v.2.3.0)^33, 34^. Only variants with at least two copies of the minor allele present in any of the four 1000 Genomes continental panels were imputed. In addition to calculating the quality metrics output by IMPUTE2, we also calculated oevar (the ratio of the observed variance of imputed dosages to the expected binomial variance) by using the MaCH imputation software^35^. We assessed overall imputation quality by looking at the distribution of imputed quality metrics across the MAF spectrum and by examining results from the IMPUTE2 internal masking experiments. We performed downstream association analyses on the results 27,887,661 variants, and considered only variants with imputation quality oevar >0.3 and MAF ≥1%.

### Outcomes

We analyzed five blood pressure outcomes. Systolic and diastolic blood pressure (SBP, DBP), Pulse Pressure (PP), defined as SBP-DBP, and Mean Arterial Pressure (MAP), defined as DBP + 1/3PP. The SBP and DBP values used were adjusted for hypertensive medication use, by adding 5 mmHg to DBP values and 10 mmHg to SBP values. Hypertension was defined by an indication of antihypertensive drug use, or by either SBP ≥ 140 mmHg or DBP ≥ 90 mmHg. For all outcomes, we excluded 95 individuals with inconsistencies in their measured SBP or DBP (Omron mean and mean of raw measures difference ≥5 mmHg), 19 individuals with high degree of Asian ancestry, 328 individuals with missing covariates or outcomes, and 70 individuals with either SBP < 80 or DBP < 50. In addition, we removed a single individual with negative PP value. We winsorized two outlying extreme values to have the value of the mean +6 standard deviations of the PP distribution, calculated on the analyzed sample set.

### Genetic analysis groups

Genetic analysis groups^23^ were constructed based on a combination of self-identified Hispanic/Latino background and genetic similarity, and are classified as Cuban, Dominican, and Puerto Rican (Caribbean groups); and Mexican, Central American, and South American (Mainland groups). The average proportions of three continental ancestries (European, African and Native American) differ among these groups, with Caribbean groups having more African and less Native American ancestry than the Mainland groups.

### Association Testing

To study the association between genotypes and any trait of interest, while controlling for population structure, we use mixed models, either linear for quantitative traits, or logistic for hypertension^36^. All models were adjusted for sex, age, age squared, study center, BMI, sampling weights to prevent potential selection bias resulting from the study design as fixed effects, the 5 first principal components estimated from the autosomal chromosome, and the 2 first principal components estimated from the X-chromosome to account for population stratification on both the autosomes and the X-chromosome. Finally, we used random effects for genetic relatedness (kinship) in the autosomes and in the X-chromosome, and random effects accounting for environmental correlations corresponding to household and community (block unit).

In all analyses, we set the threshold for follow-up at p-value < 1 × 10^−7^ and MAF ≥ 1% in the appropriate sample. SNP associations passing these thresholds were further studied in conditional analyses if they were less than 1 Mbp away from a formerly reported BP locus, and in replication testing.

#### Stratified analyses

For quantitative traits, we performed a stratified analysis in which each genetic analysis group was analyzed separately, and then the association analysis results were meta-analyzed for the Caribbean group, for the Mainland group, and for all groups. We use the MetaCor method^37^, which accounts for the correlations between the genetic analysis groups in the meta-analysis.

Since there is no appropriate method to meta-analyze analysis by subgroups for binary traits, when some of the subgroups’ individuals are correlated with each other, we performed three hypertension analyses for Mainland, Caribbean, and all individuals together.

### Allelic heterogeneity analysis

To study potential allelic heterogeneity in known BP-associated loci, we examined loci that were highly associated with at least one of the BP traits of interest, and were also within a region of 1 Mbp around a known BP locus. We conducted a conditional analysis that was performed in the same manner as the main association analysis, with the added known index SNP as a covariate in the regression model. If the detected SNP-trait association was still highly significant, it suggests allelic heterogeneity at the region.

### Replication of discovery loci in independent follow-up samples

To study the replication of detected loci in independent studies, we tested our significant and suggestive associations, when available, in the 1982 Pelotas Birth Cohort Study of admixed Southern Brazilians^38, 39^ (n = 2,764), the COGENT study of individuals of African descent^12^ (n = 22,000–32,000), and in two studies of European ancestry: the WHI study of European American women^40, 41^ (n = 14,900–17,200), and the UK Biobank^10^ (n = 140,886). The criterion for significant replication was p-value below 0.05/21 = 0.0024, where 21 was the number of follow-up tests, i.e. the combined number of tested SNPs across traits and ancestries. More information about these studies is provided in the Supplementary Information.

### Generalization of previously reported associations to the HCHS/SOL

We performed generalization analysis^25^ for BP-associated SNPs previously reported in GWASs of other populations, including those of European^6–9, 42^, African^12, 13^, Chinese^11, 43^, and multiple^14, 16, 17^ ancestries. For^17^, we also tested for generalization the reported SNP associations that were not validated and had *p*-value < 10^−5^ in their combined meta-analysis. We controlled for the directional false-discovery rate (FDR) of the generalization null hypotheses whenever direction of effect was published in the previous results, and we did not control for directionality when generalizing SNP-trait associations published in ref. 13, since directions of associations were not provided. The generalization null hypothesis states that the effect does not exist in both the discovery study and HCHS/SOL and is rejected if there is enough evidence that a SNP affects the outcome, with the same direction of effect, in both the discovery study and HCHS/SOL. We used the number of SNPs tested in the discovery study and the *p*-values for the set of tested SNPs from both the discovery study and HCHS/SOL, and we computed an *r*-value for each of the SNPs to quantify the evidence for generalization. A SNP was generalized if its *r*-value < 0.05. We also report association results for both the Caribbean and Mainland groups separately, to glean into potential effect heterogeneity between the groups in this set of SNP-trait associations.

## Electronic supplementary material

Supplementary Information
