# Supplementary material for: Genome-Wide Association Study of Blood Pressure Traits by Hispanic/Latino Background: the Hispanic Community Health Study/Study of Latinos
Source: Sci Rep. 2017 Sep 4;7:10348. doi: 10.1038/s41598-017-09019-1 (PMC5583292; doi:10.1038/s41598-017-09019-1)
Supplement: Supplementary file 1 — Supplementary Information [file 41598_2017_9019_MOESM1_ESM.pdf]

Genome-Wide Association Study of Blood Pressure Traits by Hispanic/Latino Background: the Hispanic Community Health Study/Study of Latinos - Supplementary Information

Contents

1 Manhattan and QQ plots from GWAS of blood pressure traits 2

1.1 SBP 2

1.2 DBP 4

1.3 MAP 6

1.4 PP 8

1.5 HT 10

2 Additional tables describing BP traits associations with the top SNPs 12

3 Regional association plots and forest plots 17

3.1 SBP 18

3.2 DBP 22

3.3 MAP 24

3.4 PP 27

4 Generalization of known SNP-trait associations 29

5 Replication studies 70

5.1 COGENT 70

5.2 WHI 70

5.3 UK Biobank 71

5.3.1 UK Biobank replication acknowledgements 72

5.4 1982 Pelotas Birth Cohort Study 74

6 Reproducibility **74**

1 Manhattan and QQ plots from GWAS of blood pressure traits

1.1 SBP

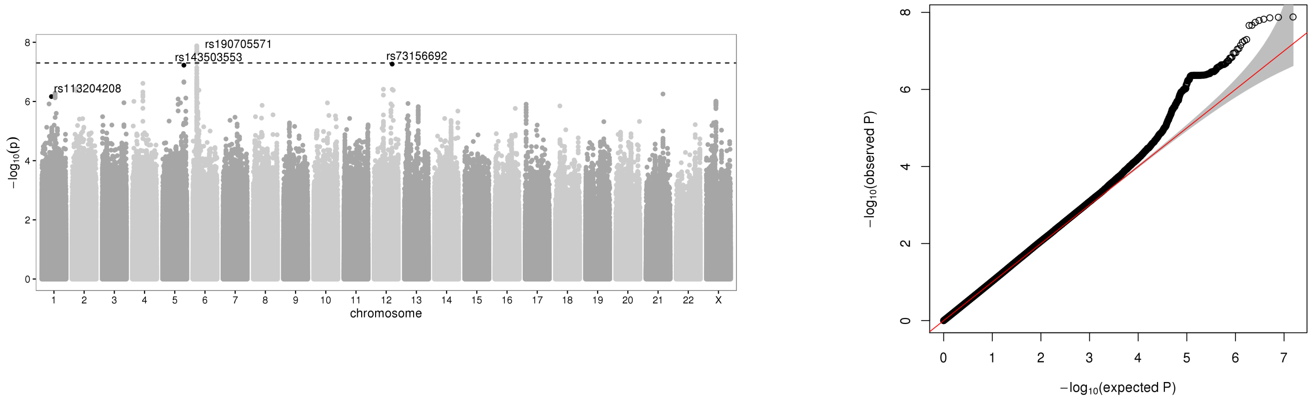

Fig. S1: Manhattan and QQ plots of SBP GWAS meta-analyzed across all genetic analysis groups. SNPs were filtered by MAF$>$ 0.1% and imputation quality oevar$\geq$ 0.3. The inflation factor is $\lambda_{gc} = 1.028$. The SNPs reported in Tables 2 and 3 of the manuscript as associated with SBP are highlighted.

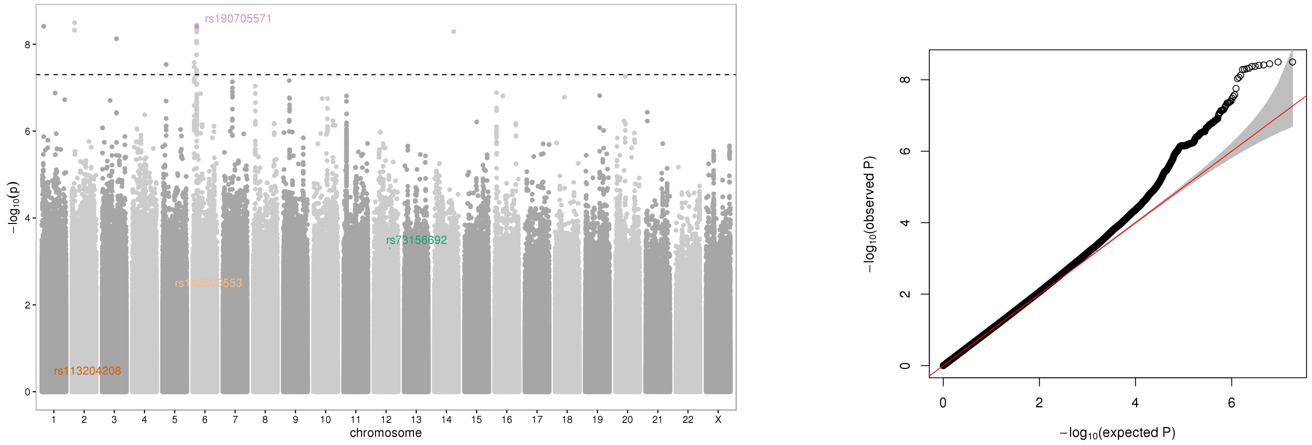

Fig. S2: Manhattan and QQ plots of SBP GWAS meta-analyzed across the Mainland genetic analysis groups. SNPs were filtered by MAF> 0.1% and imputation quality oevar≥ 0.3. The inflation factor is $\lambda_{gc} = 1.032$. The SNPs reported in Tables 2 and 3 of the manuscript as associated with SBP are highlighted.

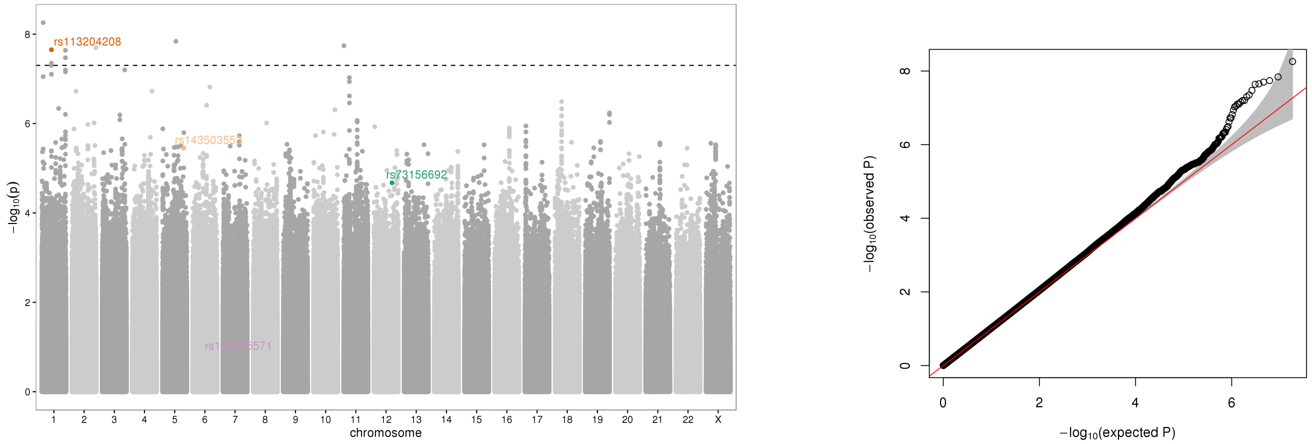

Fig. S3: Manhattan and QQ plots of SBP GWAS meta-analyzed across the Caribbean genetic analysis groups. SNPs were filtered by MAF> 0.1% and imputation quality oevar≥ 0.3. The inflation factor is $\lambda_{gc} = 1.023$. The SNPs reported in Tables 2 and 3 of the manuscript as associated with SBP are highlighted.

1.2 DBP

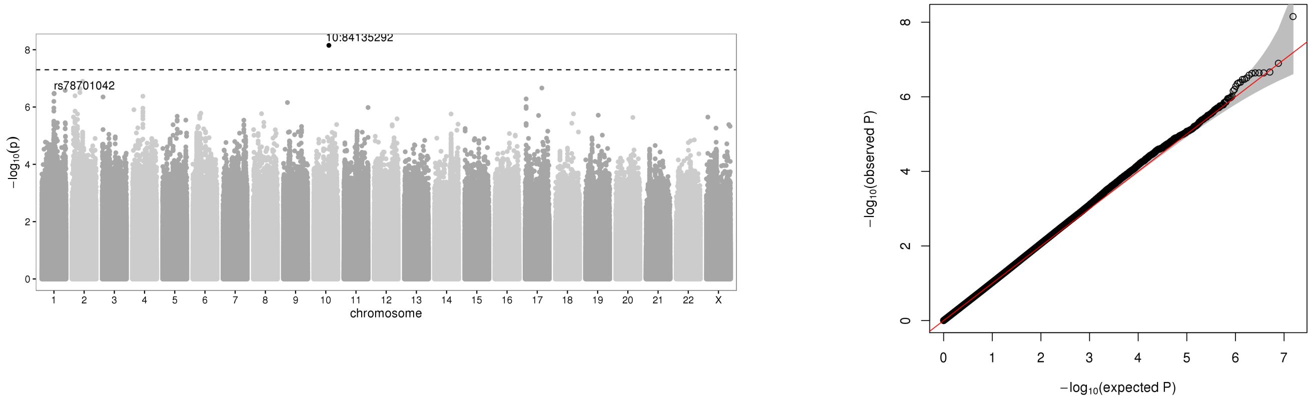

Fig. S4: Manhattan and QQ plots of DBP GWAS meta-analyzed across all genetic analysis groups. SNPs were filtered by MAF$>0.1\%$ and imputation quality oevar≥ 0.3. The inflation factor is $\lambda_{gc}=1.027$. The SNPs reported in Tables 2 and 3 of the manuscript as associated with DBP are highlighted.

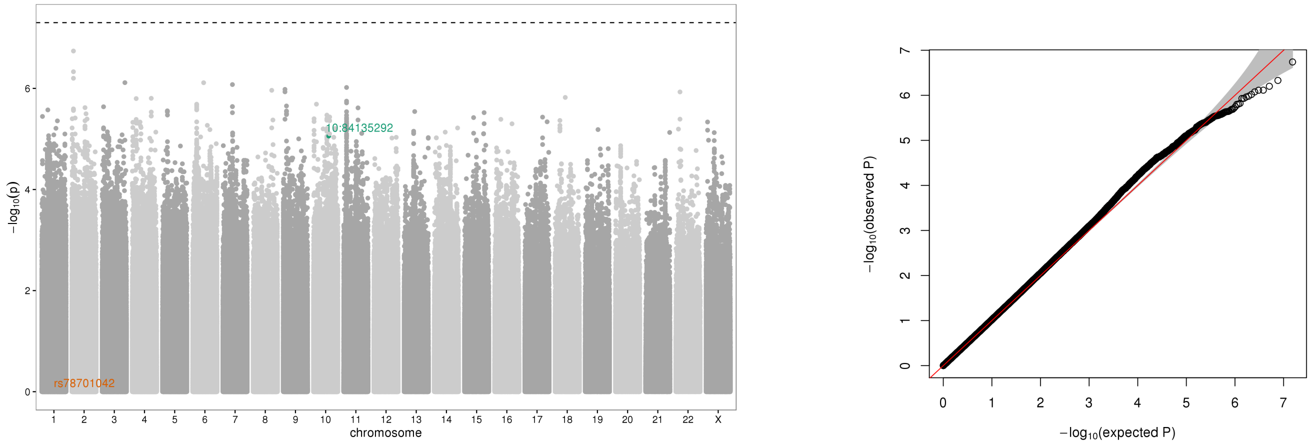

Fig. S5: Manhattan and QQ plots of DBP GWAS meta-analyzed across the Mainland genetic analysis groups. SNPs were filtered by MAF$>$ 0.1% and imputation quality oevar$\geq$ 0.3. The inflation factor is λ_{gc} = 1.023. The SNPs reported in Tables 2 and 3 of the manuscript as associated with DBP are highlighted.

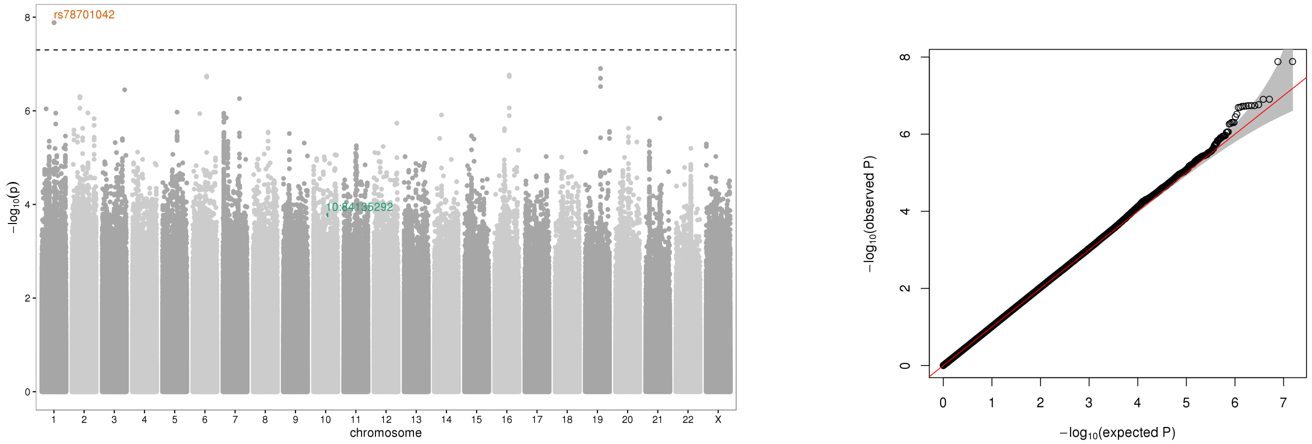

Fig. S6: Manhattan and QQ plots of DBP GWAS meta-analyzed across the Caribbean genetic analysis groups. SNPs were filtered by MAF$>$ 0.1% and imputation quality oevar$\geq$ 0.3. The inflation factor is λ_{gc} = 1.016. The SNPs reported in Tables 2 and 3 of the manuscript as associated with DBP are highlighted.

1.3 MAP

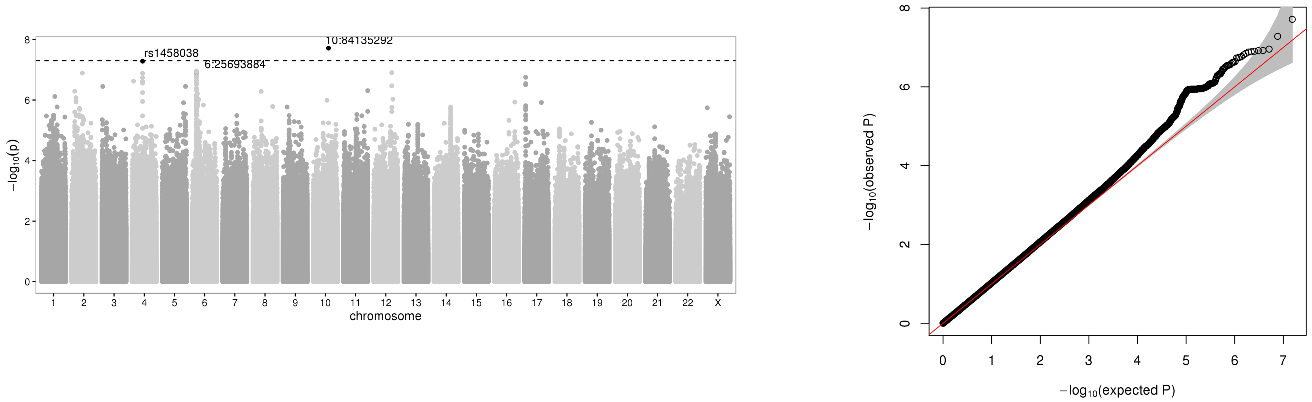

Fig. S7: Manhattan and QQ plots of MAP GWAS meta-analyzed across all genetic analysis groups. SNPs were filtered by MAF$>$ 0.1% and imputation quality oevar$\geq$ 0.3. The inflation factor is $\lambda_{gc} = 1.027$. The SNPs reported in Tables 2 and 3 of the manuscript as associated with MAP are highlighted.

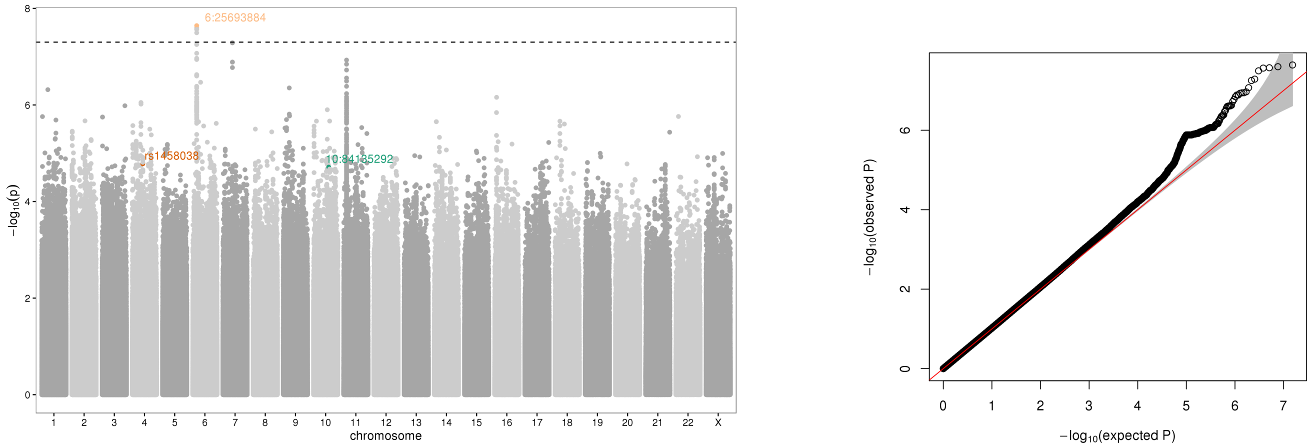

Fig. S8: Manhattan and QQ plots of MAP GWAS meta-analyzed across the Mainland genetic analysis groups. SNPs were filtered by MAF> 0.1% and imputation quality oevar≥ 0.3. The inflation factor is λ_{gc} = 1.030. The SNPs reported in Tables 2 and 3 of the manuscript as associated with MAP are highlighted.

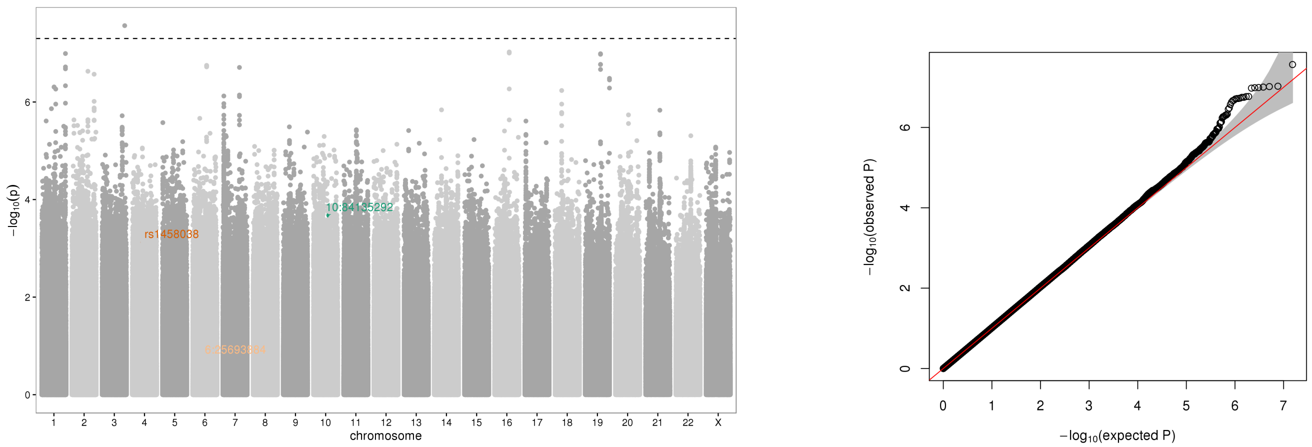

Fig. S9: Manhattan and QQ plots of MAP GWAS meta-analyzed across the Caribbean genetic analysis groups. SNPs were filtered by MAF> 0.1% and imputation quality oevar≥ 0.3. The inflation factor is λ_{gc} = 1.020. The SNPs reported in Tables 2 and 3 of the manuscript as associated with MAP are highlighted.

1.4 PP

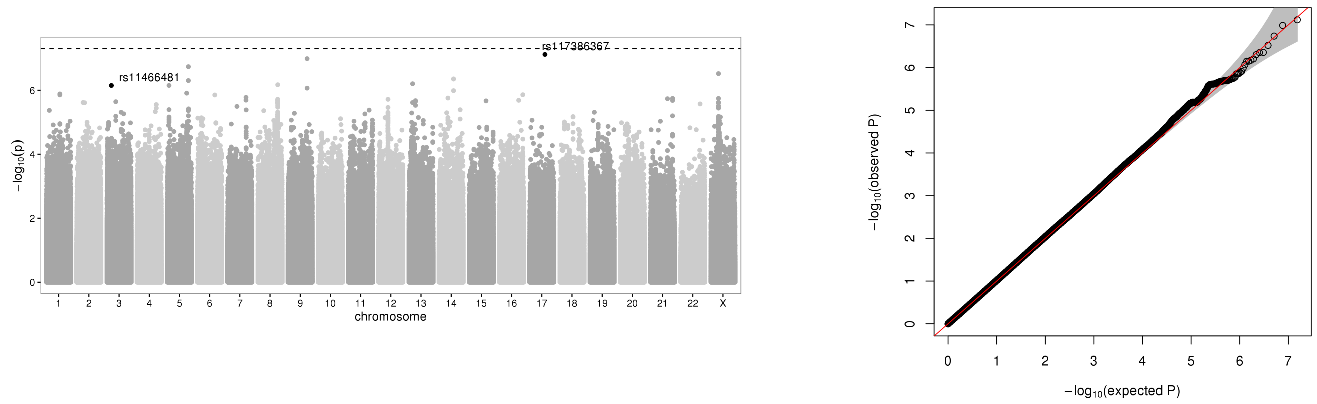

Fig. S10: Manhattan and QQ plots of PP GWAS meta-analyzed across all genetic analysis groups. SNPs were filtered by MAF$>0.1\%$ and imputation quality oevar≥ 0.3. The inflation factor is $\lambda_{gc} = 1.021$. The SNPs reported in Tables 2 and 3 of the manuscript as associated with PP are highlighted.

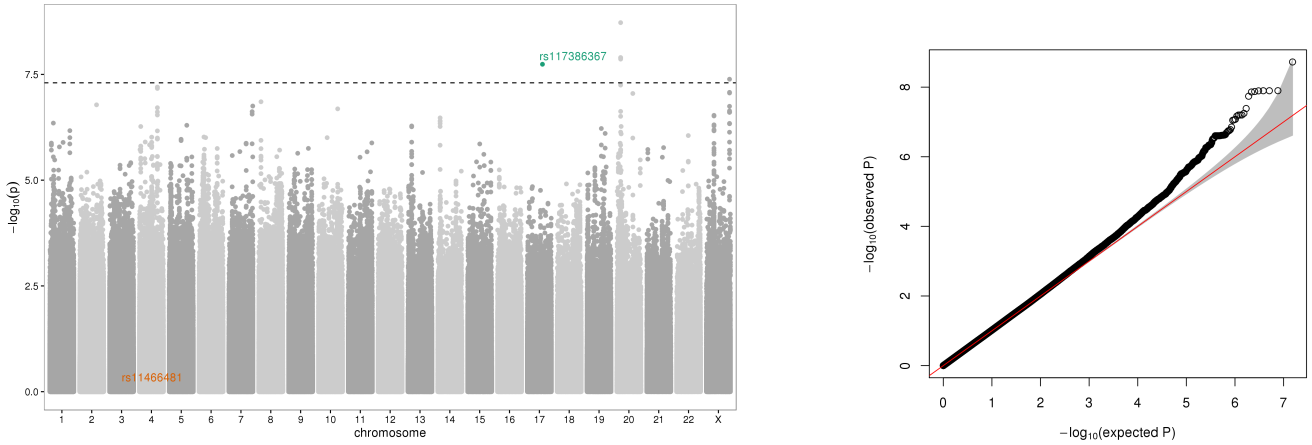

Fig. S11: Manhattan and QQ plots of PP GWAS meta-analyzed across the Mainland genetic analysis groups. SNPs were filtered by MAF> 0.1% and imputation quality oevar≥ 0.3. The inflation factor is $\lambda_{gc} = 1.023$. The SNPs reported in Tables 2 and 3 of the manuscript as associated with PP are highlighted.

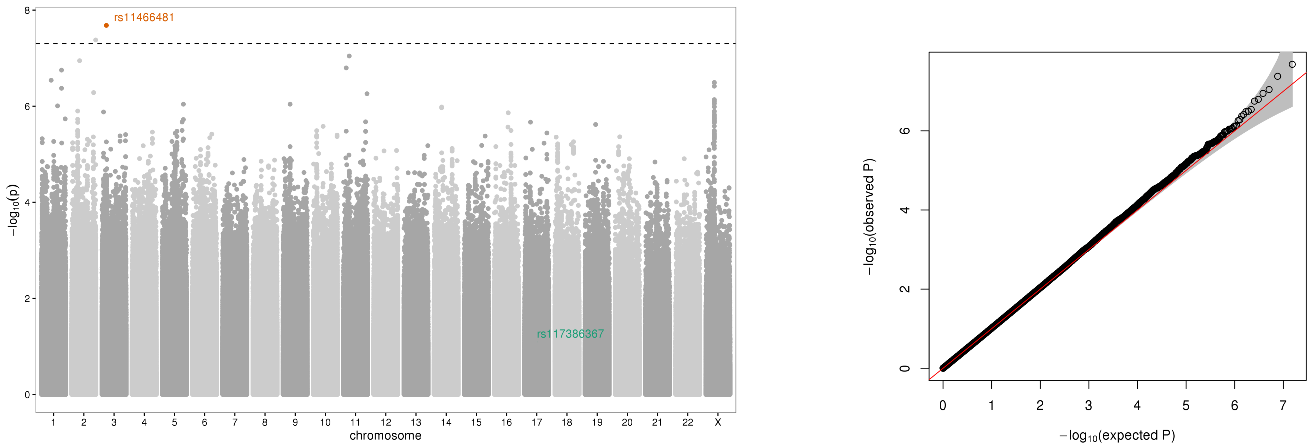

Fig. S12: Manhattan and QQ plots of PP GWAS meta-analyzed across the Caribbean genetic analysis groups. SNPs were filtered by MAF> 0.1% and imputation quality oevar≥ 0.3. The inflation factor is $\lambda_{gc} = 1.018$. The SNPs reported in Tables 2 and 3 of the manuscript as associated with PP are highlighted.

1.5 HT

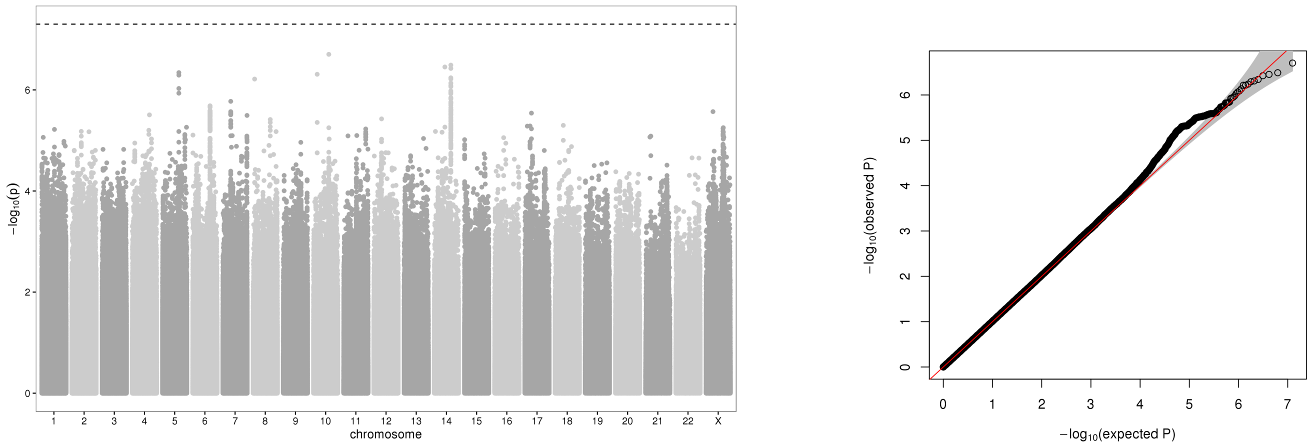

Fig. S13: Manhattan and QQ plots of HT GWAS meta-analyzed across all genetic analysis groups. SNPs were filtered by MAF$>0.1\%$ and imputation quality oevar≥ 0.3. The inflation factor is $\lambda_{gc} = 1.006$.

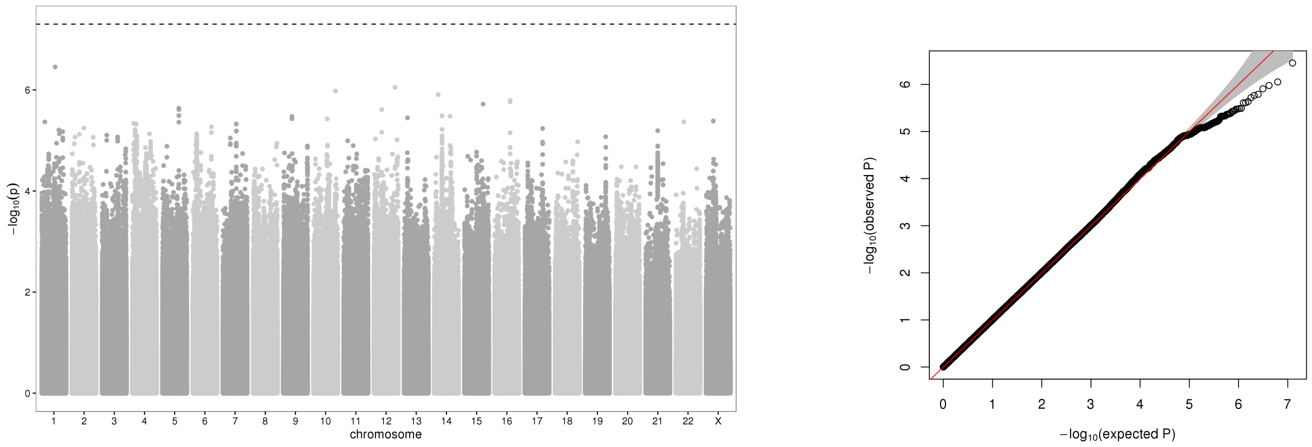

Fig. S14: Manhattan and QQ plots of HT GWAS meta-analyzed across the Mainland genetic analysis groups. SNPs were filtered by MAF$>0.1\%$ and imputation quality oevar≥ 0.3. The inflation factor is $\lambda_{gc} = 1.007$.

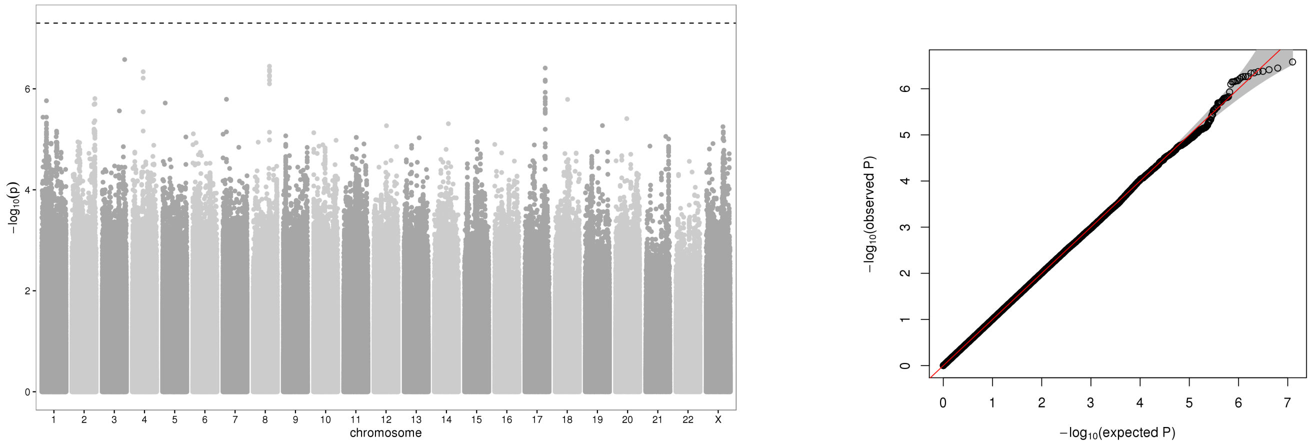

Fig. S15: Manhattan and QQ plots of HT GWAS meta-analyzed across the Caribbean genetic analysis groups. SNPs were filtered by MAF> 0.1% and imputation quality oevar≥ 0.3. The inflation factor is $\lambda_{gc} = 1.005$.

2 Additional tables describing BP traits associations with the top SNPs

Table S1 provides the estimated associations of the SNPs reported in Tables 2 and 3 in the main manuscript with all BP traits. The estimates are from the analyses that combined individuals from all the genetic analysis groups. Table S2 provides estimated associations of SNPs reported in Table 3 of the main manuscript with all BP traits. Here, the estimated associations are provided only in the subgroups (Mainland, Caribbean) in which the reported associations were discovered. Finally, Table S3 provides local ancestry estimates from all variants reported in Tables 2 and 3 of the main manuscript (except for one variant that did not have inferred local ancestry). These estimates were obtained by the ASAFE software (Zhang et al., 2016) applied on inferred local ancestry estimates from the HCHS/SOL (Browning et al., 2016).

rsID	Chr	position	DBP		SBP		MAP		PP		HT	
			beta	*p*-value	beta	*p*-value	beta	*p*-value	beta	*p*-value	OR	*p*-value
rs1458038	4	81164723	-0.80	$4.2e \times 10^{-7}$	-1.30	2.44×10^{-7}	-0.97	5.22×10^{-8}	-0.49	0.00×10^{-4}	0.85	9.90×10^{-6}
rs143503553	5	159593663	-3.64	8.00×10^{-5}	-7.99	5.94×10^{-8}	-5.07	1.23×10^{-6}	-4.40	3.82×10^{-6}	0.57	0.00×10^{-2}
rs190705571	6	25693887	-0.61	1.74×10^{-5}	-1.27	2.16×10^{-8}	-0.83	2.29×10^{-7}	-0.66	7.25×10^{-6}	0.87	7.07×10^{-5}
NA	10	84135292	-0.94	7.05×10^{-9}	-1.22	2.97×10^{-6}	-1.03	1.93×10^{-8}	-0.27	0.11×10^{-5}	0.81	6.82×10^{-8}
rs73156692	12	101608695	-0.87	4.13×10^{-6}	-1.65	5.44×10^{-8}	-1.13	1.25×10^{-7}	-0.76	0.00×10^{-2}	0.87	0.00×10^{-7}
rs117386367	17	53098512	0.50	0.57×10^{-9}	5.57	0.00×10^{-1}	2.18	0.03×10^{-2}	5.01	7.61×10^{-8}	1.60	0.02×10^{-4}
rs113204208	1	90549106	-2.64	0.00×10^{-9}	-5.78	6.83×10^{-7}	-3.69	6.86×10^{-6}	-3.23	1.78×10^{-5}	0.52	5.61×10^{-5}
rs78701042	1	115841602	-4.38	3.40×10^{-7}	-4.19	0.00×10^{-9}	-4.31	1.03×10^{-5}	0.23	0.79×10^{-7}	0.53	1.82×10^{-3}
rs11466481	3	30664148	-0.75	0.10×10^{-8}	-3.09	3.78×10^{-5}	-1.53	0.00×10^{-6}	-2.40	$7.1e \times 10^{-7}$	0.82	0.06×10^{-6}
NA	6	25693884	0.63	9.12×10^{-6}	1.28	1.39×10^{-8}	0.85	1.21×10^{-7}	0.66	8.06×10^{-6}	1.15	8.02×10^{-5}

Table S1: SNPs associations with all BP traits, for the SNPs reported in Tables 2 and 3 in the main manuscript, estimated in a meta-analysis of all genetic analysis groups.

rsID	Chr	position	group	DBP		SBP		MAP		PP		HT	
				beta	p-value	beta	p-value	beta	p-value	beta	p-value	OR	p-value
rs113204208	1	90549106	Caribbean	-3.31	0.00×10^{-3}	-7.92	2.22×10^{-8}	-4.83	1.37×10^{-6}	-4.67	2.88×10^{-7}	0.40	1.86×10^{-6}
rs78701042	1	115841602	Caribbean	-5.44	1.30×10^{-8}	-5.53	0.00×10^{-7}	-5.49	4.91×10^{-7}	-0.05	0.95×10^{-8}	0.47	0.00×10^{-4}
rs11466481	3	30664148	Caribbean	-0.54	0.32×10^{-7}	-3.68	3.24×10^{-5}	-1.59	0.01×10^{-5}	-3.18	2.08×10^{-8}	0.84	0.13×10^{-7}
NA	6	25693884	Mainland	0.83	2.05×10^{-6}	1.66	3.57×10^{-9}	1.11	2.27×10^{-8}	0.82	8.65×10^{-6}	1.24	1.16×10^{-5}

Table S2: SNPs associations with all BP traits, for the SNPs reported in Table 3 in the main manuscript, in the subgroups in which these associations had p-value1×10^{-7}.

rsID	chromosome	position	A1	A2	type	oevar	AFR	AMR	EUR
rs78701042	1	115841602	T	C	i	1.00	0.05	0.00	0.00
rs113204208	1	90549106	G	C	i	0.97	0.06	0.00	0.00
rs1458038	4	81164723	T	C	g	1.00	0.03	0.31	0.25
rs1458038	4	81164723	T	C	g	1.00	0.03	0.31	0.25
rs143503553	5	159593663	G	C	i	0.93	0.00	0.00	0.01
	6	25693884	GATT	G	i	1.06	0.85	0.29	0.75
rs190705571	6	25693887	T	G	i	1.06	0.86	0.29	0.75
rs9366626	6	25684953	G	A	i	1.00	0.75	0.25	0.62
	10	84135292	CA	C	i	0.86	0.64	0.37	0.12
	10	84135292	CA	C	i	0.86	0.64	0.37	0.12
rs73156692	12	101608695	A	G	i	0.99	0.13	0.01	0.23
rs117386367	17	53098512	A	G	i	0.78	0.00	0.00	0.01

Table S3: Local ancestry estimates for most of the loci reported in Tables 2 and 3 of the main manuscript, as estimated in the HCHS/SOL data set. The frequencies are of the allele A1. AFR is the frequency estimate in the African ancestry, AMR in Amerindian ancestry, and EUR in the European ancestry component of the HCHS/SOL. Type is the variant type: imputed (i) or genotyped (g). oevar is a measure of imputation accuracy.

					DBP		SBP		MAP		PP	
Analysis	Type	rsID	Chr	position	beta	p-value	beta	p-value	beta	p-value	beta	p-value
primary	index	rs2240736	17	59485393	0.16	2.46E-01	0.23	2.96E-01	0.18	2.43E-01	0.08	5.86E-01
primary	lead	rs117386367	17	53098512	0.5	5.79E-01	5.57	1.01E-04	2.18	3.12E-02	5.01	7.61E-08
conditional	lead	rs117386367	17	53098512	0.4	6.57E-01	5.4	1.66E-04	2.06	4.24E-02	4.95	1.12E-07
primary	index	rs11953630	5	157845402	-0.02	8.89E-01	-0.36	1.60E-01	-0.13	4.65E-01	-0.33	5.09E-02
primary	lead	rs143503553	5	159593663	-3.64	8.00E-05	-7.99	5.94E-08	-5.07	1.23E-06	-4.4	3.82E-06
conditional	lead	rs143503553	5	159593663	-3.72	5.73E-05	-7.98	6.57E-08	-5.11	1.01E-06	-4.31	6.14E-06
primary	index	rs1799945	6	26091179	-0.06	7.79E-01	-0.35	2.93E-01	-0.16	5.07E-01	-0.3	1.69E-01
primary	lead	rs190705571	6	25693887	-0.61	1.74E-05	-1.27	2.16E-08	-0.83	2.29E-07	-0.66	7.25E-06
conditional	lead	rs190705571	6	25693887	-0.62	1.50E-05	-1.26	3.97E-08	-0.83	2.58E-07	-0.64	1.72E-05

Table S4: Results from conditional analyses. For each genome-wide significant loci in the HCHS/SOL analysis in which there was a previously reported “index SNP” less than 1Mbp away, we report the association results for both the known index SNP and the detected HCHS/SOL “lead SNP” in the primary HCHS/SOL analysis, and the association testing results for the lead SNP in the conditional analysis that adjusted for the known index SNPs.

3 Regional association plots and forest plots

For each of the reported SNPs in Tables 2 and 3 of the main manuscript, we provide a forest plot comparing the SNP-trait association testing results across the genetic analysis groups, the Mainland group, the Caribbean group, and all groups combined. In addition, we provide the regional association (LocusZoom) with p-values and LDs calculated from the Mainland group, Caribbean group, and all groups combined. Each of the regional association figures is centered around a lead variant, and displays the LDs and p-values of variants in a 1Mbp region around it. Each symbol on the plot correspond to a specific variant. If this variant is imputed, the symbol is either an x, or a purple inverted triangle (if it is the lead variant). If the variant is genotyped, its symbol is a circle, or a purple diamond (if it is the lead variant). The colors of the non-lead variants correspond to their LD with the lead variant, where this LD was calculated as the squared Pearson correlation between the genotype counts/dosages of the lead variant and those of that variant, and based on the population represented in the figure. Thus, if the regional association plot corresponds to the combined analysis ("All"), we used the entire HCHS/SOL sample set to calculate LD. If it corresponds to the Caribbean group, we used only individuals classified as Caribbeans. The y-axis position of the symbols corresponds to the $-\log(p\text{-value})$ of the variants in the analysis. Finally, the blue line represents recombination rates, taken from HapMap (Gibbs et al., 2003).

3.1 SBP

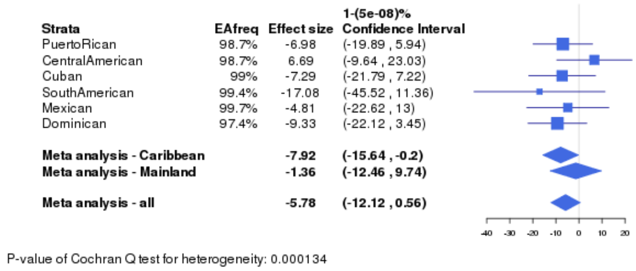

(a) Forest plot

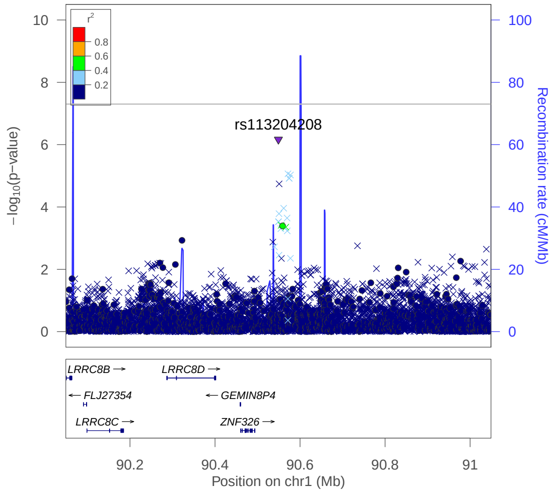

(b) Regional association: All

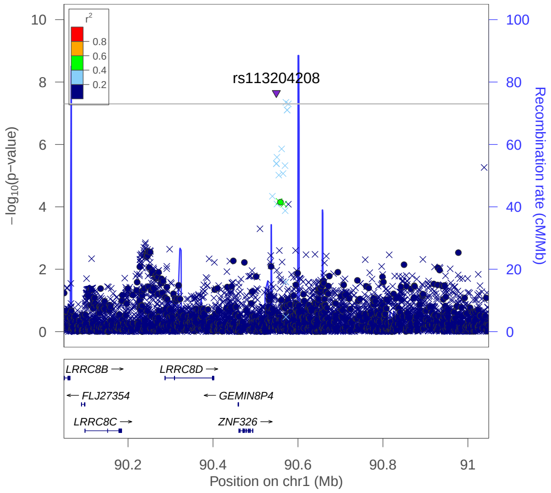

(c) Regional association: Caribbean

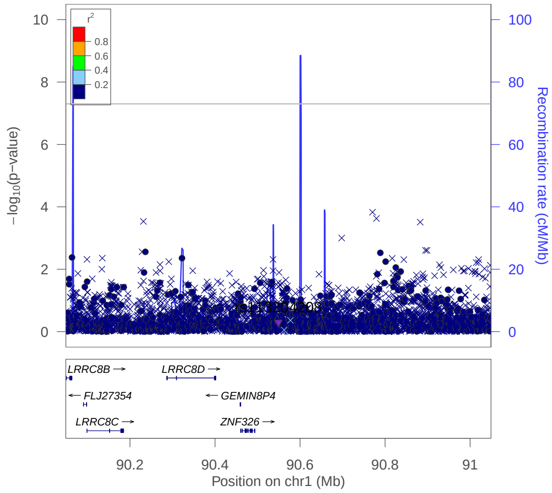

(d) Regional association: Mainland

Fig. S16: Forest plot and regional association plots for the SBP locus (lead SNP rs113204208) on chromosome 1.

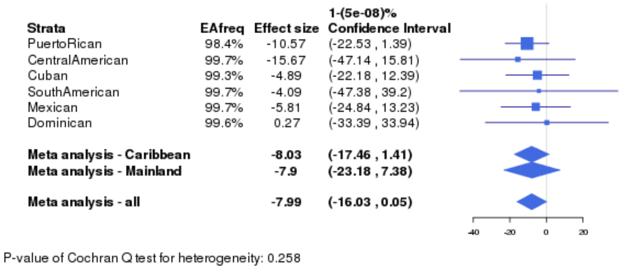

(a) Forest plot

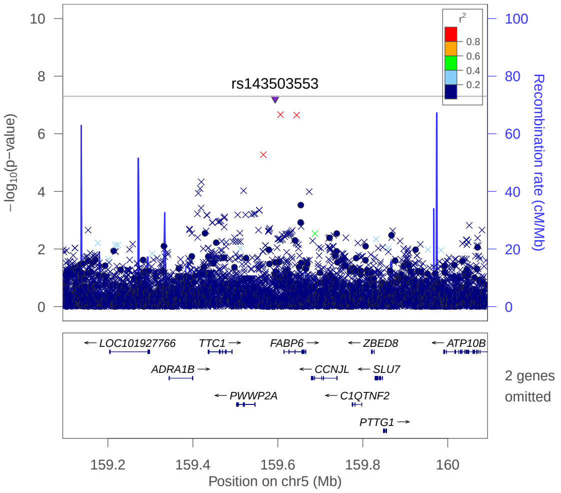

(b) Regional association: All

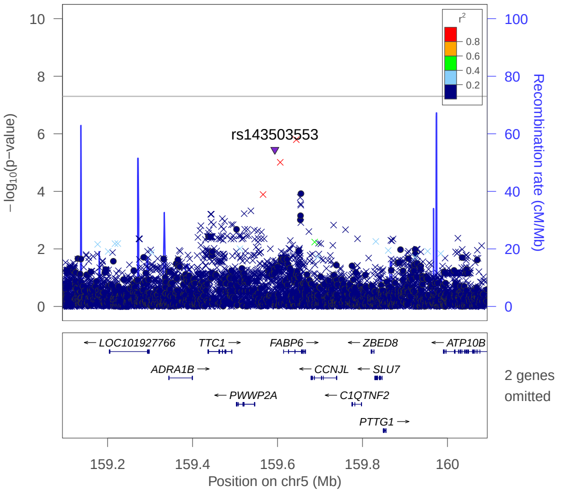

(c) Regional association: Caribbean

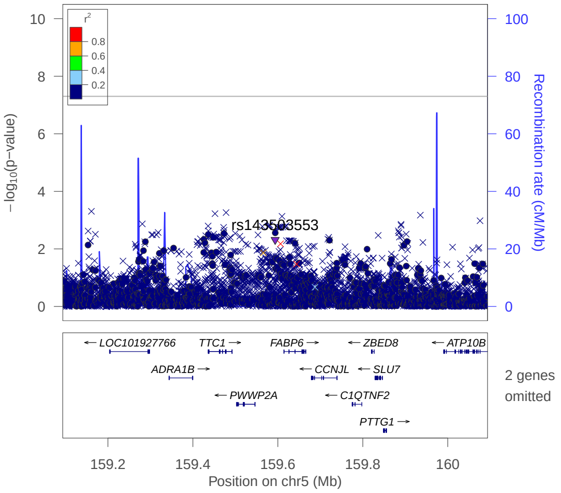

(d) Regional association: Mainland

Fig. S17: Forest plot and regional association plots for the SBP locus (lead SNP rs143503553) on chromosome 5.

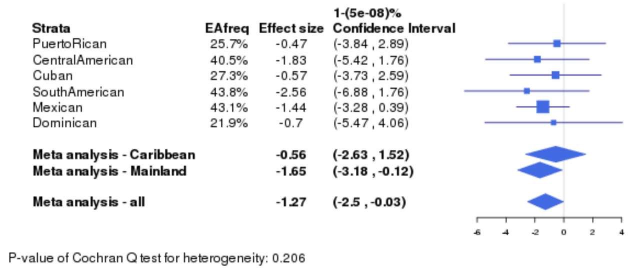

(a) Forest plot

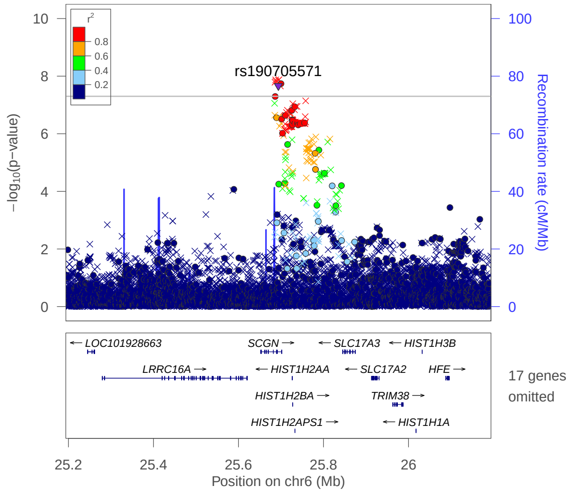

(b) Regional association: All

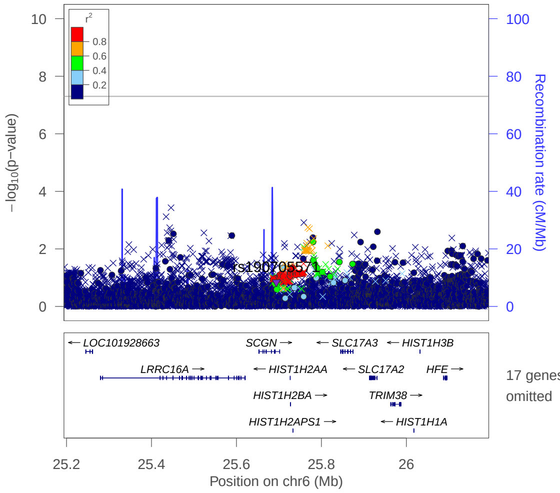

(c) Regional association: Caribbean

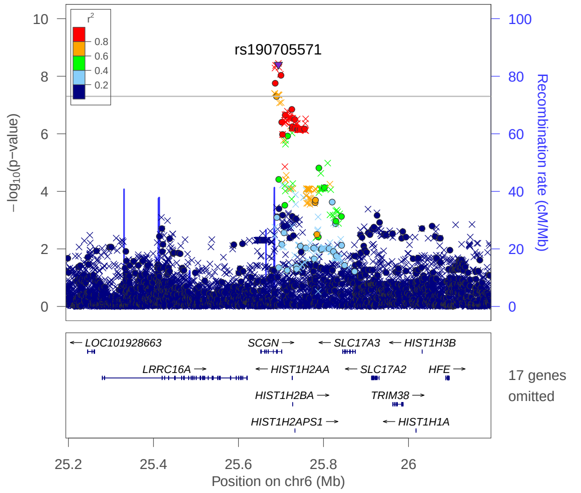

(d) Regional association: Mainland

Fig. S18: Forest plot and regional association plots for the SBP locus (lead SNP rs190705571) on chromosome 6.

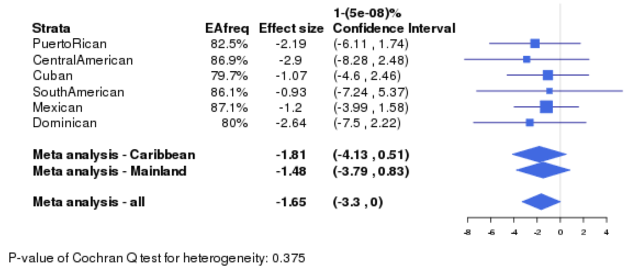

(a) Forest plot

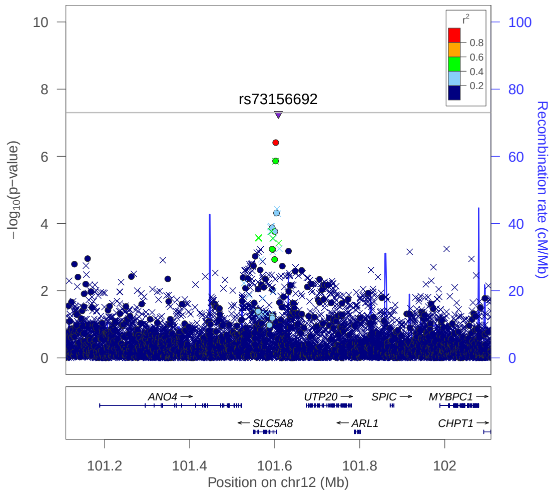

(b) Regional association: All

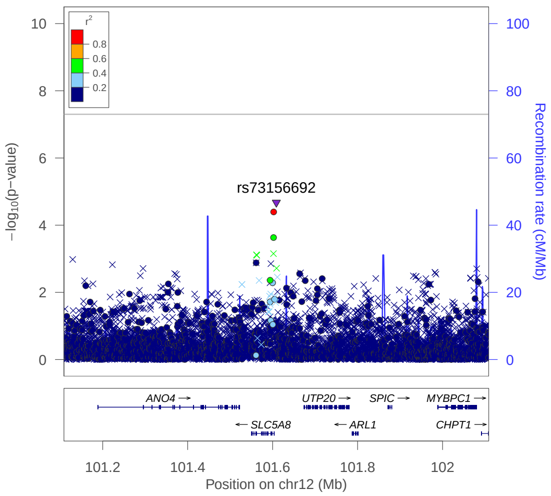

(c) Regional association: Caribbean

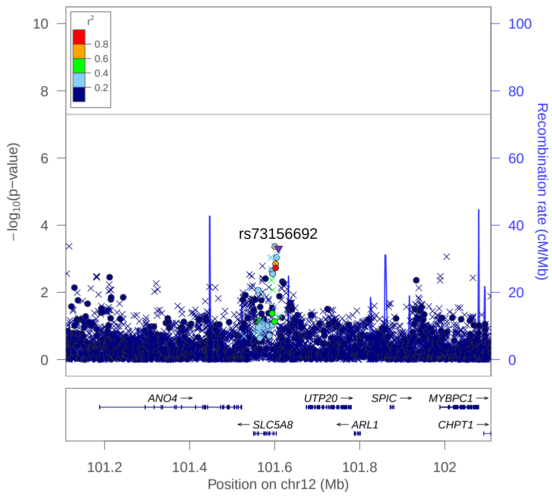

(d) Regional association: Mainland

Fig. S19: Forest plot and regional association plots for the SBP locus (lead SNP rs73156692) on chromosome 12.

3.2 DBP

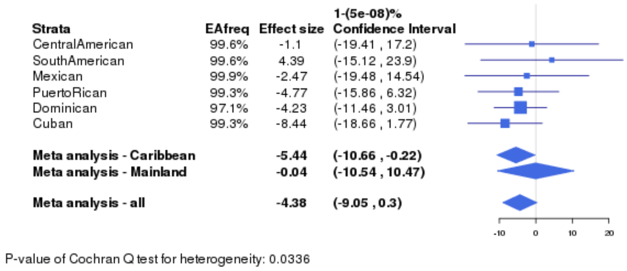

(a) Forest plot

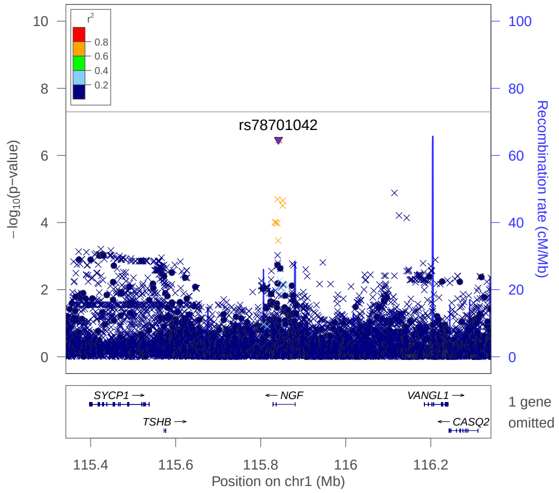

(b) Regional association: All

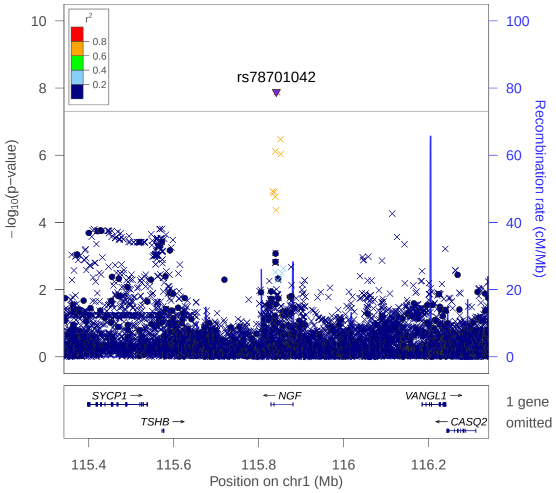

(c) Regional association: Caribbean

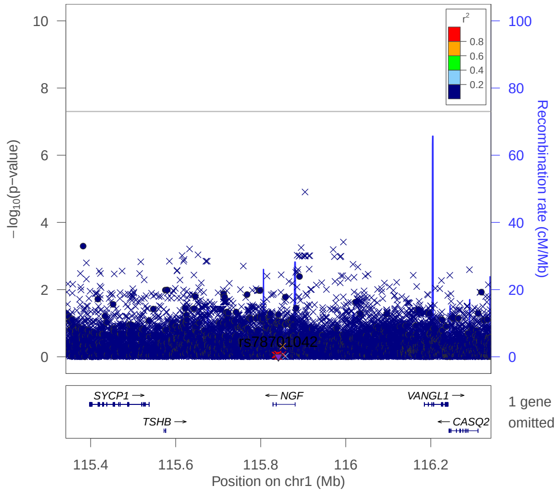

(d) Regional association: Mainland

Fig. S20: Forest plot and regional association plots for the DBP locus (lead SNP rs78701042) on chromosome 1.

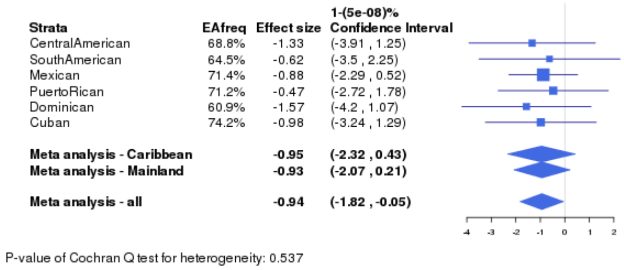

(a) Forest plot

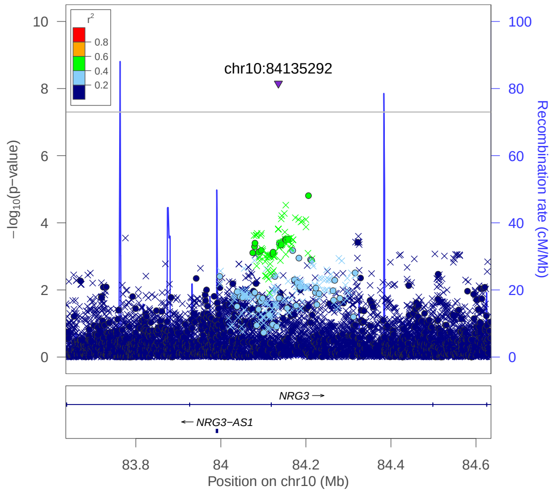

(b) Regional association: All

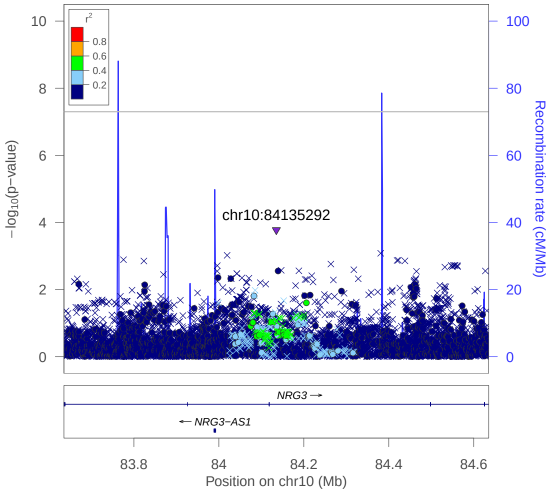

(c) Regional association: Caribbean

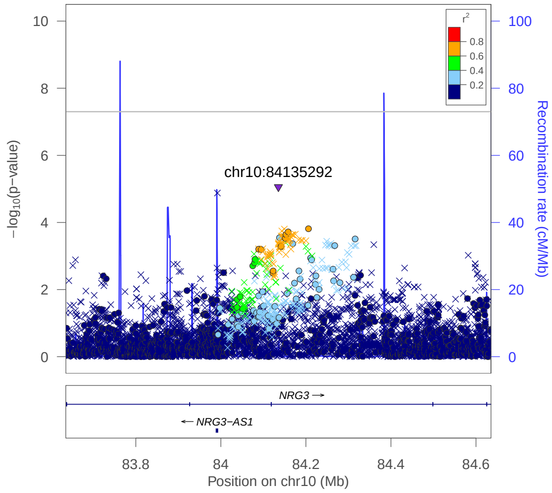

(d) Regional association: Mainland

Fig. S21: Forest plot and regional association plots for the DBP locus on chromosome 10, lead SNP position 84135292.

3.3 MAP

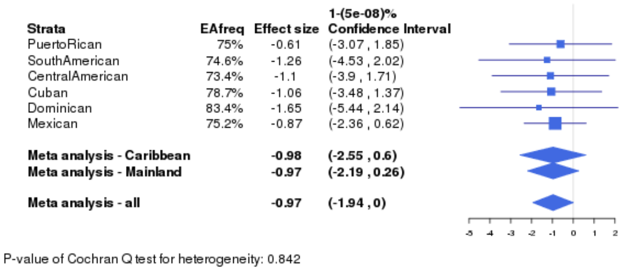

(a) Forest plot

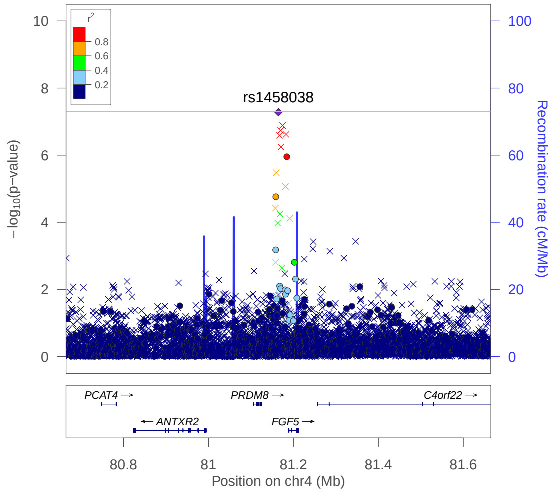

(b) Regional association: All

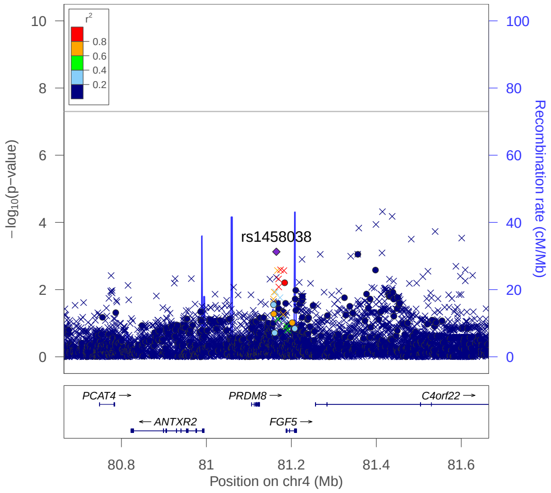

(c) Regional association: Caribbean

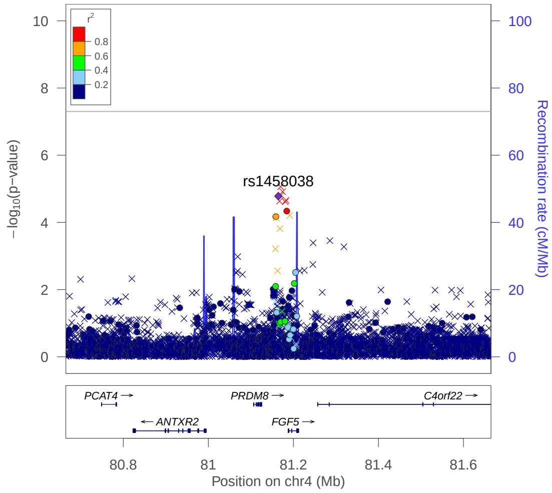

(d) Regional association: Mainland

Fig. S22: Forest plot and regional association plots for the MAP locus (lead SNP rs1458038) on chromosome 4.

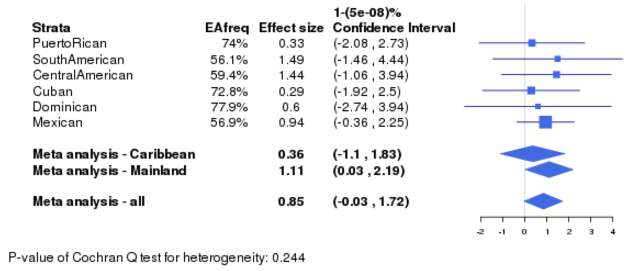

(a) Forest plot

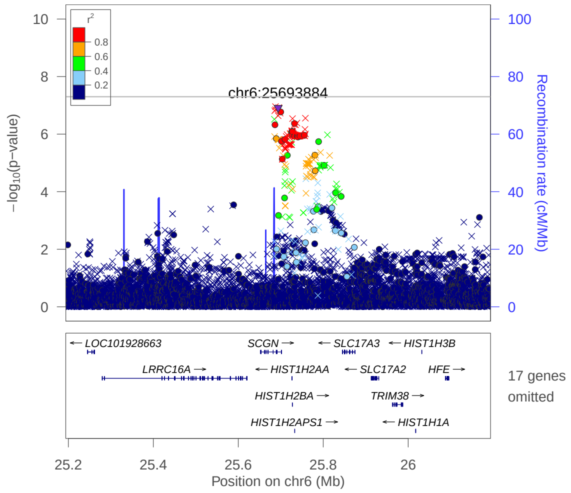

(b) Regional association: All

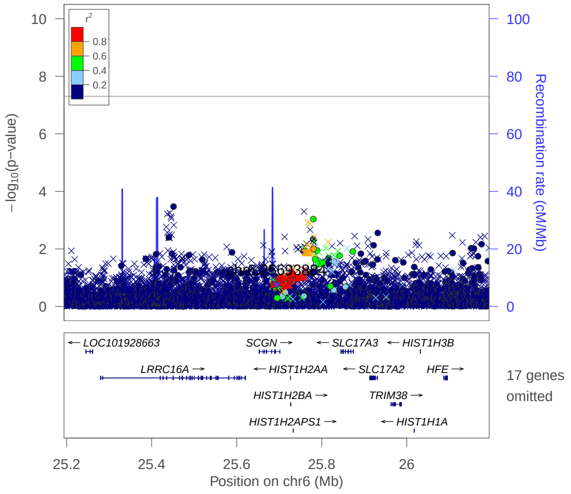

(c) Regional association: Caribbean

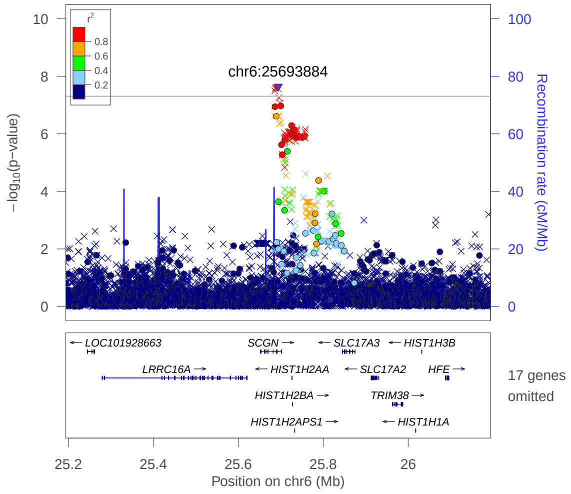

(d) Regional association: Mainland

Fig. S23: Forest plot and regional association plots for the MAP locus on chromosome 6 (lead SNP position 19858891).

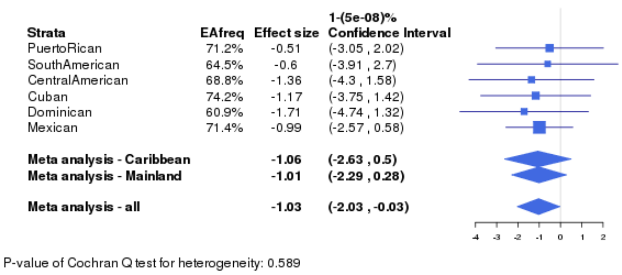

(a) Forest plot

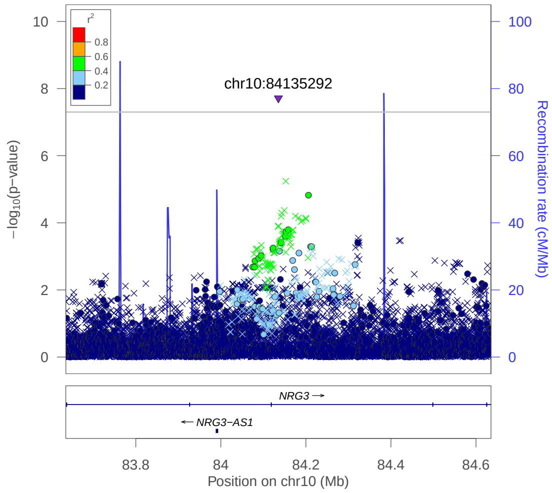

(b) Regional association: All

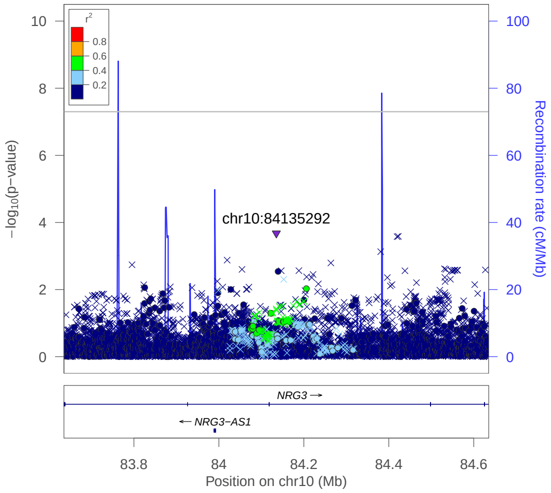

(c) Regional association: Caribbean

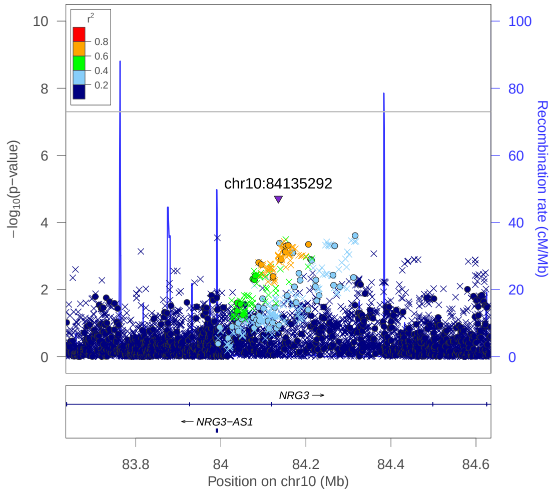

(d) Regional association: Mainland

Fig. S24: Forest plot and regional association plots for the MAP locus on chromosome 10 (lead SNP position 84135292).

3.4 PP

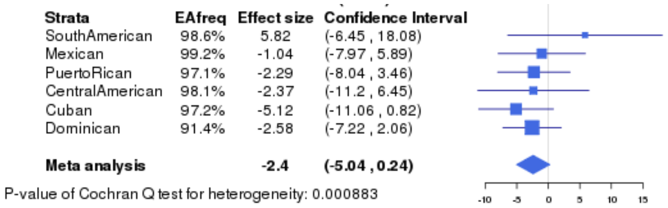

(a) Forest plot

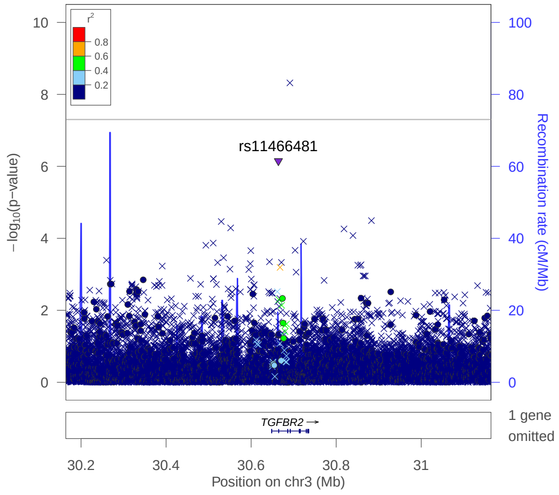

(b) Regional association: All

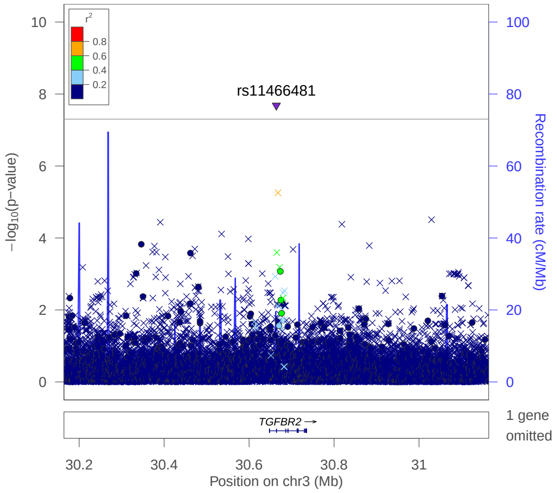

(c) Regional association: Caribbean

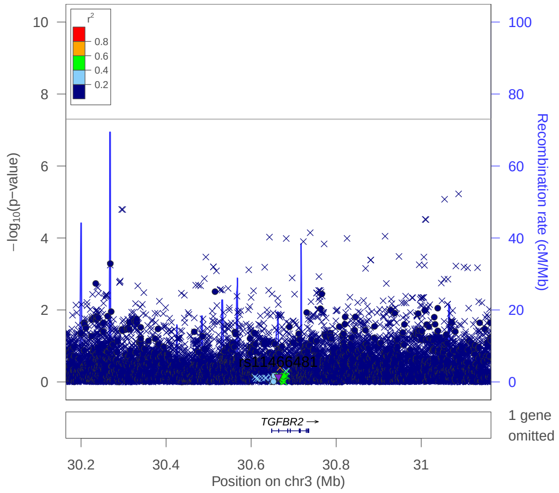

(d) Regional association: Mainland

Fig. S25: Forest plot and regional association plots for the PP locus on chromosome 3 (lead SNP rs11466481).

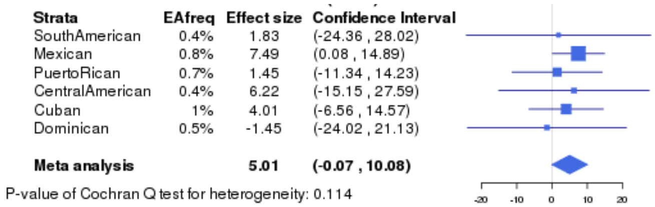

(a) Forest plot

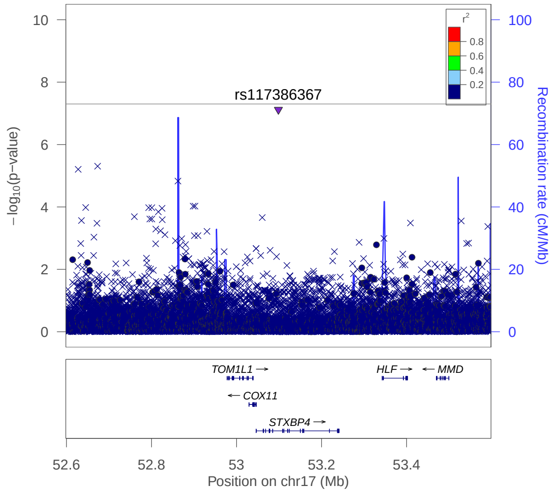

(b) Regional association: All

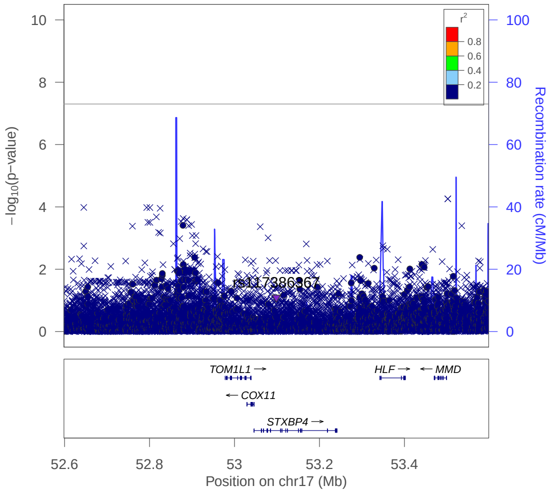

(c) Regional association: Caribbean

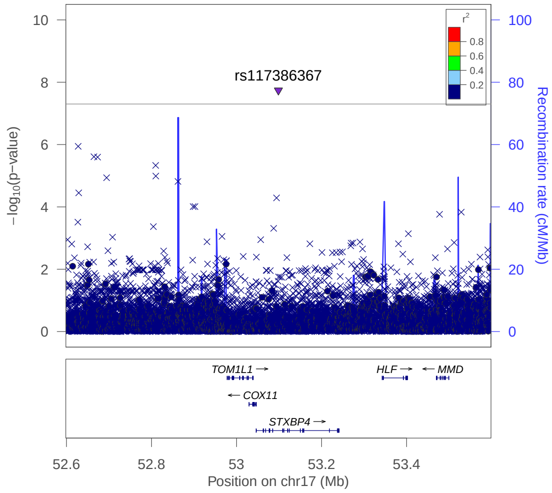

(d) Regional association: Mainland

Fig. S26: Forest plot and regional association plots for the PP locus on chromosome 17 (lead SNP rs117386367).

4 Generalization of known SNP-trait associations

For each previously reported analysis (published in a given paper, or 'discovery study', with a specific trait) we provide a table of generalization analysis results. We compare the effect sizes provided in the discovery study (if available) and the effect sizes estimated in the Caribbean group, Mainland group, and all groups combined in the HCHS/SOL, as well as corresponding p-values and FDR_g r-values. We also provide fixed-effects meta-analysis results for the discovery associations and the HCHS/SOL associations, from the analysis combining all genetic analysis groups.

rsID	chr	position	trait	A1	discovery	HCHS/SOL beta			HCHS/SOL p-value			FDR$_g$ r-value		
					p-value	Mainland	Caribbean	All	Mainland	Caribbean	All	Mainland	Caribbean	All
rs11120313	1	214581206	DBP	A	4.53E-06	0.18	0.20	0.19	0.48	0.52	0.34	1.00	1	1.00
rs16848861	1	237144914	DBP	G	4.73E-06	0.83	-0.34	-0.00	0.26	0.46	0.99	1.00	1	1.00
rs16853574	3	169079763	DBP	C	5.10E-06	-0.22	-0.17	-0.20	0.51	0.70	0.45	1.00	1	1.00
rs9590141	13	95603622	DBP	A	8.76E-07	0.72	0.63	0.67	0.12	0.13	0.03	1.00	1	0.64
rs9301196	13	107847432	DBP	T	6.66E-06	0.55	-0.45	0.11	0.01	0.05	0.45	1.00	1	1.00
rs11846013	14	46932291	DBP	A	4.99E-06	0.47	0.04	0.29	0.01	0.86	0.04	1.00	1	1.00
rs10135446	14	80409478	DBP	A	4.47E-06	0.10	-0.29	-0.04	0.60	0.27	0.81	1.00	1	1.00
rs1867226	15	91523713	DBP	C	5.80E-07	-0.15	-0.11	-0.13	0.42	0.62	0.35	1.00	1	1.00
rs8039294	15	91743859	DBP	G	6.29E-06	-0.57	-0.09	-0.29	0.07	0.72	0.15	1.00	1	1.00
rs2823756	21	17742330	DBP	T	5.73E-06	-0.09	0.32	0.09	0.63	0.14	0.55	1.00	1	1.00
rs5743185	2	190737838	SBP	T	2.09E-11	-1.08	-0.16	-0.64	0.13	0.83	0.21	0.94	1	1.00
rs13201744	6	6126845	SBP	A	1.12E-06	0.31	-0.31	0.04	0.57	0.62	0.92	1.00	1	1.00
rs16877320	6	15923026	SBP	G	3.42E-09	-0.46	0.36	0.02	0.64	0.66	0.97	1.00	1	1.00
rs17365948	8	101956877	SBP	A	1.59E-08	1.44	-0.18	0.51	0.14	0.83	0.42	0.94	1	1.00
rs2183737	9	71241633	SBP	T	1.21E-06	-0.91	-0.65	-0.74	0.25	0.23	0.10	1.00	1	1.00
rs12279202	11	9432090	SBP	A	4.80E-08	0.06	-0.32	-0.14	0.95	0.72	0.83	1.00	1	1.00
rs11160059	14	92807330	SBP	A	1.54E-08	0.74	-0.83	-0.53	0.61	0.24	0.41	1.00	1	1.00
rs3751664	16	1254369	SBP	T	6.71E-08	-0.37	1.19	0.43	0.62	0.10	0.41	1.00	1	1.00
rs4613079	16	80643957	SBP	T	5.06E-07	0.25	-0.98	0.00	0.47	0.14	0.99	1.00	1	1.00
rs11659639	18	58167612	SBP	C	2.13E-07	-3.57	-0.15	-2.01	0.03	0.93	0.09	0.52	1	1.00

Table S5: Generalization analysis results for SNP associations reported in Adeyemo et al.

Table S6: Generalization of previously reported HT loci.

Ref	rsID	chr	position	trait	A1	discovery		HCHS/SOL OR			HCHS/SOL p-value			FDR_g r-value			all meta	
						OR	p-value	Main	Carib	All	Main	Carib	All	Main	Carib	All	OR	p-value
Adeyemo	rs2146204	1	168874099	HT	C	2.49	2.97E-06	0.96	1.11	1.04	5.77E-01	1.40E-01	4.26E-01	1.00	0.70	1.00	1.11	4.62E-02
Adeyemo	rs12748299	1	198983324	HT	C	1.99	1.66E-05	1.08	0.87	0.95	2.49E-01	1.22E-02	2.20E-01	1.00	1.00	1.00	1.00	9.01E-01
Adeyemo	rs11692045	2	41950953	HT	C	0.64	1.54E-05	0.96	1.08	1.03	4.87E-01	1.29E-01	4.98E-01	1.00	1.00	1.00	0.97	3.62E-01
Adeyemo	rs11714139	3	131944637	HT	T	2.45	1.14E-05	1.00	1.06	1.03	9.96E-01	4.51E-01	5.98E-01	1.00	1.00	1.00	1.09	1.00E-01
Adeyemo	rs991316	4	100322445	HT	T	1.62	3.45E-06	0.90	1.09	0.99	2.76E-02	7.34E-02	8.34E-01	1.00	0.70	1.00	1.04	2.33E-01
Adeyemo	rs9791170	5	131569627	HT	A	0.58	5.10E-07	1.01	1.02	1.02	7.64E-01	6.22E-01	6.15E-01	1.00	1.00	1.00	0.97	3.35E-01
Adeyemo	rs11988036	8	20140207	HT	T	1.66	1.95E-05	1.01	0.97	0.99	8.57E-01	4.93E-01	7.73E-01	1.00	1.00	1.00	1.03	3.55E-01
Adeyemo	rs7902529	10	2298418	HT	A	0.50	6.14E-06	0.97	0.99	0.98	6.62E-01	9.35E-01	6.36E-01	1.00	1.00	1.00	0.92	7.14E-02
Adeyemo	rs1550576	15	58213414	HT	T	0.52	1.03E-05	1.00	1.00	1.00	9.43E-01	9.65E-01	9.49E-01	1.00	1.00	1.00	0.95	1.94E-01
Adeyemo	rs2665797	17	61922485	HT	G	0.45	1.73E-05	1.05	0.91	0.98	3.24E-01	5.14E-02	5.01E-01	1.00	1.00	1.00	0.95	1.48E-01
HMG	rs880315	1	10796866	HT	C	1.10	1.99E-09	1.06	1.02	1.04	1.90E-01	7.57E-01	2.47E-01	0.60	1.00	0.52	1.09	9.07E-09
Ehret	rs17367504	1	11862778	HT	G	0.90	2.30E-10	0.85	0.87	0.86	6.01E-02	5.68E-02	6.93E-03	0.44	0.41	0.06	0.90	7.50E-12
HMG	rs10745332	1	113189053	HT	A	1.12	2.70E-09	1.06	1.20	1.14	4.42E-01	1.56E-03	3.01E-03	1.00	0.03	0.03	1.12	1.41E-10
Ehret	rs2932538	1	113216543	HT	G	1.05	2.90E-07	1.06	1.22	1.15	4.59E-01	8.83E-04	2.36E-03	1.00	0.20	0.06	1.05	1.66E-08
HMG	rs16849225	2	164906820	HT	C	1.04	9.69E-03	1.00	1.07	1.03	9.54E-01	2.35E-01	4.48E-01	1.00	1.00	1.00	1.04	5.68E-03
Ehret	rs13082711	3	27537909	HT	T	0.97	3.60E-04	0.93	0.91	0.92	2.46E-01	1.13E-01	5.50E-02	1.00	1.00	1.00	0.96	9.33E-05
HMG	rs820430	3	27548900	HT	A	1.06	1.12E-06	1.01	1.08	1.04	8.96E-01	1.37E-01	2.41E-01	1.00	0.68	0.52	1.06	4.88E-07
Levy	rs7640747	3	37596805	HT	G	1.13	4.80E-07	1.04	0.99	1.01	5.21E-01	7.94E-01	8.30E-01	0.87	1.00	0.89	1.10	8.01E-08
Levy	rs743395	3	37598382	HT	T	1.13	7.50E-07	1.03	0.98	1.00	5.82E-01	7.24E-01	9.38E-01	0.87	1.00	0.94	1.10	1.22E-07

Table S6: Generalization of previously reported HT loci.

Ref	rsID	chr	position	trait	A1	discovery		HCHS/SOL OR			HCHS/SOL p-value			FDR$_g$ r-value			all meta	
						OR	p-value	Main	Carib	All	Main	Carib	All	Main	Carib	All	OR	p-value
Ehret	rs3774372	3	41877414	HT	T	0.98	1.80E-01	0.89	0.95	0.92	1.01E-01	3.39E-01	7.41E-02	1.00	1.00	1.00	0.98	7.55E-02
HMG	rs9815354	3	41912651	HT	A	1.01	7.94E-01	1.12	1.07	1.09	1.26E-01	2.71E-01	6.74E-02	1.00	1.00	1.00	1.02	2.94E-01
HMG	rs9810888	3	53635595	HT	G	1.06	3.60E-06	0.99	1.04	1.02	8.08E-01	3.31E-01	6.26E-01	1.00	0.80	0.92	1.05	5.46E-06
HMG	rs1902859	4	81157703	HT	C	1.14	7.61E-18	1.19	1.06	1.13	8.13E-04	2.50E-01	1.23E-03	0.02	0.79	0.02	1.14	2.34E-20
Ehret	rs1458038	4	81164723	HT	T	1.07	1.90E-07	1.24	1.13	1.18	5.80E-05	3.27E-02	9.90E-06	0.06	0.32	0.06	1.09	1.52E-10
Ehret	rs13107325	4	103188709	HT	T	0.90	4.90E-07	0.92	1.09	1.03	5.24E-01	3.77E-01	7.16E-01	1.00	1.00	1.00	0.91	1.96E-06
HMG	rs13143871	4	156619204	HT	T	1.09	1.24E-06	1.08	1.07	1.08	1.66E-01	1.71E-01	4.82E-02	0.60	0.68	0.30	1.09	3.99E-07
Ehret	rs13139571	4	156645513	HT	C	1.04	2.50E-05	1.08	1.09	1.09	1.42E-01	1.04E-01	2.64E-02	1.00	1.00	1.00	1.05	3.48E-06
Ehret	rs1173771	5	32815028	HT	G	1.06	3.20E-10	1.15	1.04	1.09	4.13E-03	3.99E-01	8.54E-03	0.06	1.00	0.06	1.07	1.24E-11
Ehret	rs11953630	5	157845402	HT	T	0.95	1.70E-07	0.87	0.98	0.93	1.85E-02	6.29E-01	6.55E-02	0.18	1.00	0.32	0.95	3.29E-08
Ehret	rs1799945	6	26091179	HT	G	1.10	1.80E-10	1.06	1.05	1.05	4.22E-01	4.78E-01	2.92E-01	1.00	1.00	0.70	1.10	1.42E-10
HMG	rs1799945	6	26091179	HT	G	1.18	3.98E-05	1.06	1.05	1.05	4.22E-01	4.78E-01	2.92E-01	1.00	1.00	0.92	1.13	8.55E-05
HMG	rs9266359	6	31332739	HT	C	1.05	1.33E-05	0.98	1.00	0.99	7.72E-01	9.96E-01	7.80E-01	1.00	1.00	1.00	1.05	9.94E-05
Ehret	rs805303	6	31616366	HT	G	1.06	1.10E-10	1.02	1.05	1.04	6.80E-01	2.58E-01	2.88E-01	1.00	0.93	0.70	1.05	7.27E-11
HMG	rs2021783	6	32044851	HT	C	1.10	3.53E-11	1.27	0.95	1.15	5.90E-02	7.73E-01	1.68E-01	0.37	1.00	0.52	1.10	7.93E-12
Ehret	rs4373814	10	18419972	HT	G	0.96	8.50E-08	0.99	1.01	1.00	8.68E-01	7.98E-01	9.44E-01	1.00	1.00	1.00	0.96	2.47E-07
Ehret	rs1813353	10	18707448	HT	T	1.08	6.20E-10	1.01	1.02	1.01	9.14E-01	7.32E-01	8.19E-01	1.00	1.00	1.00	1.07	3.12E-09
Levy	rs11014166	10	18708798	HT	T	0.90	7.80E-07	1.00	0.98	0.99	9.62E-01	6.45E-01	8.15E-01	1.00	0.75	0.89	0.92	6.44E-07
Ehret	rs4590817	10	63467553	HT	G	1.10	9.80E-09	1.03	0.99	1.00	7.15E-01	8.54E-01	9.99E-01	1.00	1.00	1.00	1.09	5.58E-08

Table S6: Generalization of previously reported HT loci.

Ref	rsID	chr	position	trait	A1	discovery		HCHS/SOL OR			HCHS/SOL p-value			FDR$_g$ r-value			all meta	
						OR	p-value	Main	Carib	All	Main	Carib	All	Main	Carib	All	OR	p-value
Ehret	rs932764	10	95895940	HT	G	1.06	9.40E-09	1.03	0.98	1.01	4.79E-01	7.03E-01	8.20E-01	1.00	1.00	1.00	1.05	2.37E-08
HMG	rs4409766	10	104616663	HT	T	1.12	7.33E-13	1.16	0.97	1.07	1.26E-02	6.29E-01	1.38E-01	0.12	1.00	0.52	1.11	5.69E-12
Ehret	rs11191548	10	104846178	HT	T	1.10	1.40E-05	1.17	0.97	1.09	8.75E-03	7.37E-01	6.07E-02	1.00	1.00	1.00	1.10	2.27E-06
Ehret	rs7129220	11	10350538	HT	G	0.96	1.10E-03	1.01	0.79	0.86	9.08E-01	1.91E-03	1.32E-02	1.00	1.00	1.00	0.95	1.92E-04
HMG	rs4757391	11	16302939	HT	C	1.08	3.33E-06	1.08	1.05	1.06	1.28E-01	3.39E-01	8.34E-02	0.60	0.80	0.40	1.07	2.94E-06
Ehret	rs381815	11	16902268	HT	T	1.06	3.40E-06	1.13	0.98	1.05	1.88E-02	7.03E-01	1.52E-01	0.67	1.00	0.59	1.06	1.20E-06
Ehret	rs633185	11	100593538	HT	G	0.93	5.40E-11	0.97	0.99	0.98	4.89E-01	8.16E-01	4.97E-01	1.00	1.00	1.00	0.94	9.88E-11
Levy	rs4842666	12	89941549	HT	C	0.86	3.40E-07	0.92	0.91	0.91	1.53E-01	1.21E-01	3.23E-02	0.87	0.19	0.22	0.88	9.78E-08
Levy	rs11105328	12	89942390	HT	G	0.86	7.10E-07	0.89	0.88	0.88	1.48E-01	5.27E-02	1.45E-02	0.87	0.19	0.22	0.87	2.86E-08
Levy	rs2681472	12	90008959	HT	G	0.85	1.70E-08	0.97	0.89	0.92	6.58E-01	8.09E-02	1.08E-01	0.87	0.19	0.22	0.87	6.82E-08
Levy	rs2681492	12	90013089	HT	C	0.87	8.40E-08	0.97	0.91	0.94	7.31E-01	1.42E-01	1.83E-01	0.87	0.20	0.26	0.89	2.96E-06
Levy	rs11105354	12	90026523	HT	G	0.85	1.80E-08	0.97	0.89	0.92	6.49E-01	7.58E-02	9.99E-02	0.87	0.19	0.22	0.87	6.21E-08
Levy	rs12579302	12	90050503	HT	G	0.85	2.20E-08	0.98	0.89	0.93	7.82E-01	7.99E-02	1.32E-01	0.87	0.19	0.22	0.87	9.31E-08
Ehret	rs17249754	12	90060586	HT	G	1.13	1.10E-14	1.02	1.09	1.06	8.09E-01	1.78E-01	2.40E-01	1.00	0.86	0.70	1.13	1.37E-14
HMG	rs17249754	12	90060586	HT	G	1.09	2.08E-08	1.02	1.09	1.06	8.09E-01	1.78E-01	2.40E-01	1.00	0.68	0.52	1.08	8.61E-08
Levy	rs17249754	12	90060586	HT	A	0.85	2.20E-08	0.98	0.92	0.94	8.09E-01	1.78E-01	2.40E-01	0.87	0.23	0.31	0.88	2.53E-07
Levy	rs11105364	12	90069276	HT	G	0.85	2.10E-08	0.98	0.90	0.93	7.38E-01	9.77E-02	1.40E-01	0.87	0.19	0.22	0.87	1.02E-07
Levy	rs11105368	12	90074441	HT	C	0.85	2.20E-08	0.97	0.90	0.93	7.31E-01	9.64E-02	1.40E-01	0.87	0.19	0.22	0.87	1.04E-07
Levy	rs11105378	12	90090741	HT	T	0.84	2.80E-08	0.96	0.90	0.93	5.76E-01	1.21E-01	1.23E-01	0.87	0.19	0.22	0.87	1.67E-08

Table S6: Generalization of previously reported HT loci.

Ref	rsID	chr	position	trait	A1	discovery		HCHS/SOL OR			HCHS/SOL p-value			FDR$_g$ r-value			all meta	
						OR	p-value	Main	Carib	All	Main	Carib	All	Main	Carib	All	OR	p-value
Levy	rs12230074	12	90090867	HT	G	0.84	2.80E-08	0.96	0.90	0.92	5.44E-01	1.04E-01	1.04E-01	0.87	0.19	0.22	0.86	1.34E-08
Ehret	rs3184504	12	111884608	HT	T	1.06	2.60E-06	1.01	1.08	1.05	7.96E-01	1.12E-01	1.63E-01	1.00	0.65	0.59	1.06	9.48E-07
HMG	rs11066280	12	112817783	HT	T	1.11	5.78E-08	0.67	0.94	0.74	5.47E-01	9.44E-01	5.57E-01	1.00	1.00	1.00	1.11	7.71E-08
HMG	rs1991391	12	115352666	HT	G	1.00	9.57E-01	1.07	1.04	1.05	2.29E-01	4.45E-01	1.70E-01	1.00	1.00	1.00	1.02	3.90E-01
Ehret	rs10850411	12	115387796	HT	T	1.05	5.20E-06	1.08	1.07	1.07	1.13E-01	1.76E-01	3.75E-02	0.95	0.93	0.68	1.05	7.16E-07
HMG	rs35444	12	115552437	HT	A	1.08	2.86E-05	1.06	1.02	1.04	2.16E-01	6.80E-01	2.45E-01	1.00	1.00	0.67	1.07	2.38E-05
HMG	rs11067763	12	116198341	HT	A	1.06	1.37E-07	0.97	1.10	1.03	6.35E-01	9.69E-02	4.24E-01	1.00	0.68	0.73	1.06	3.40E-07
Ehret	rs1378942	15	75077367	HT	C	1.08	1.00E-08	1.02	1.01	1.01	6.84E-01	9.16E-01	7.14E-01	1.00	1.00	1.00	1.07	3.59E-08
Ehret	rs2521501	15	91437388	HT	T	1.06	7.00E-07	1.07	1.13	1.11	2.63E-01	1.55E-02	8.20E-03	0.95	0.27	0.08	1.06	3.55E-08
Ehret	rs17608766	17	45013271	HT	T	0.98	8.00E-02	1.11	0.94	1.00	3.25E-01	4.42E-01	9.75E-01	1.00	1.00	1.00	0.98	8.90E-02
Ehret	rs12940887	17	47402807	HT	T	1.05	1.20E-07	0.99	0.93	0.95	8.38E-01	1.46E-01	2.23E-01	1.00	1.00	1.00	1.04	9.52E-07
HMG	rs1887320	20	10965998	HT	A	1.06	5.13E-05	1.14	1.11	1.12	1.86E-02	2.32E-02	1.69E-03	1.00	1.00	1.00	1.07	1.69E-06
Ehret	rs1327235	20	10969030	HT	G	1.03	4.60E-04	1.14	1.11	1.12	1.80E-02	2.13E-02	1.52E-03	1.00	1.00	1.00	1.04	2.46E-05
Ehret	rs6015450	20	57751117	HT	G	1.12	4.20E-14	1.16	0.89	0.97	1.39E-01	1.15E-01	6.22E-01	0.67	1.00	1.00	1.11	5.87E-13

Ref	rsID	chr	position	trait	A1	discovery		HCHS/SOL score			HCHS/SOL p-value			FDR_g r-value		
						Z-score	p-value	Main	Carib	All	Main	Carib	All	Main	Carib	All
Liu 2016	rs11537751	11	47587452	HT	T	5.40	6.90E-08	1.31E+00	-7.81E+00	-6.29E+00	8.50E-01	3.51E-01	5.63E-01	0.85	1.00	1.00

Table S7: Generalization testing of a HT locus reported by Liu et al. (2016).

Table S8: Generalization of previously reported loci associated with quantitative BP traits.

Ref	rsID	chr	position	trait	A1	discovery		HCHS/SOL beta			HCHS/SOL p-value			FDR$_g$ r-value			all meta	
						beta	p-value	Main	Carib	All	Main	Carib	All	Main	Carib	All	beta	p-value
Hoffmann	rs7546498	1	1740255	SBP	G	0.26	2.80E-11	0.64	-0.17	0.32	2.32E-02	6.33E-01	1.46E-01	3.46E-01	1.00	6.36E-01	0.32	1.46E-01
Hoffmann	rs7546498	1	1740255	DBP	G	0.15	4.50E-10	0.27	-0.01	0.16	1.34E-01	9.78E-01	2.56E-01	6.21E-01	1.00	6.52E-01	0.16	2.56E-01
Liu	rs2493292	1	3328659	SBP	T	0.37	1.40E-08	1.18	0.05	0.60	1.36E-02	9.13E-01	7.25E-02	1.09E-01	1.00	2.90E-01	0.38	3.00E-08
Hoffmann	rs9662255	1	9441949	SBP	C	0.20	2.20E-06	0.67	-0.18	0.31	2.36E-02	6.10E-01	1.62E-01	3.46E-01	1.00	6.36E-01	0.31	1.62E-01
Hoffmann	rs9662255	1	9441949	PP	C	0.18	3.50E-10	0.39	0.00	0.23	4.13E-02	9.87E-01	1.19E-01	5.44E-01	1.00	4.96E-01	0.23	1.19E-01
Levy	rs284277	1	10790797	SBP	A	0.79	9.40E-07	-0.37	0.44	-0.04	1.91E-01	1.92E-01	8.70E-01	1.00E+00	0.37	1.00E+00	0.50	1.05E-04
HMG	rs880315	1	10796866	DBP	C	0.46	7.94E-08	0.23	-0.02	0.13	1.97E-01	9.20E-01	3.44E-01	4.69E-01	1.00	5.45E-01	0.36	1.57E-06
HMG	rs880315	1	10796866	SBP	C	0.97	5.52E-10	0.28	0.06	0.20	3.17E-01	8.69E-01	3.74E-01	6.68E-01	1.00	5.47E-01	0.71	5.31E-08
Levy	rs880315	1	10796866	SBP	T	0.89	2.10E-07	-0.28	-0.06	-0.20	3.17E-01	8.69E-01	3.74E-01	1.00E+00	1.00	1.00E+00	0.49	2.94E-04
Ehret ’11	rs17367504	1	11862778	DBP	G	-0.55	3.50E-19	-0.45	-1.00	-0.71	1.46E-01	1.92E-03	1.48E-03	2.82E-01	0.01	5.37E-03	-0.56	2.72E-21
Ehret ’11	rs17367504	1	11862778	SBP	G	-0.90	8.70E-22	-0.69	-1.34	-1.00	1.56E-01	9.97E-03	5.33E-03	3.78E-01	0.10	2.21E-02	-0.91	1.78E-23
Hoffmann	rs3820068	1	15798197	SBP	A	0.26	1.80E-07	0.05	0.44	0.26	9.12E-01	2.65E-01	3.78E-01	1.00E+00	0.89	7.88E-01	0.26	3.78E-01
Hoffmann	rs3820068	1	15798197	PP	A	0.20	1.10E-09	0.05	0.54	0.31	8.60E-01	3.56E-02	9.85E-02	1.00E+00	0.44	4.65E-01	0.31	9.85E-02
Hoffmann	rs137993948	1	23369277	PP	T	-0.20	1.00E-08	-0.30	0.15	-0.06	3.45E-01	6.07E-01	7.92E-01	1.00E+00	1.00	1.00E+00	-0.06	7.92E-01
Hoffmann	rs9729719	1	38298207	PP	G	-0.19	2.00E-11	-0.09	-0.14	-0.11	6.91E-01	5.95E-01	5.18E-01	1.00E+00	1.00	9.48E-01	-0.11	5.18E-01
Liu	rs4660293	1	40028180	DBP	G	0.18	9.60E-08	-0.25	-0.05	-0.16	3.61E-01	8.52E-01	4.33E-01	1.00E+00	1.00	1.00E+00	0.17	6.09E-09
Hoffmann	rs783621	1	42368035	SBP	A	0.29	2.90E-14	0.19	0.35	0.26	4.87E-01	3.01E-01	2.34E-01	1.00E+00	0.93	6.58E-01	0.26	2.34E-01
Hoffmann	rs783621	1	42368035	PP	A	0.21	3.10E-16	-0.02	0.35	0.13	8.94E-01	1.06E-01	3.54E-01	1.00E+00	0.66	8.20E-01	0.13	3.54E-01
Ehret ’16	rs7515635	1	42408070	SBP	T	0.31	4.81E-12	0.48	-0.01	0.27	1.01E-01	9.69E-01	2.28E-01	3.36E-01	1.00	5.86E-01	0.31	2.27E-12
Ehret ’16	rs7515635	1	42408070	DBP	T	0.14	2.05E-07	0.22	-0.05	0.10	2.28E-01	8.13E-01	4.56E-01	6.58E-01	1.00	8.62E-01	0.14	1.62E-07
Hoffmann	rs147696085	1	51021867	PP	G	0.29	2.20E-11	0.56	0.98	0.76	1.90E-01	2.74E-02	1.35E-02	7.51E-01	0.44	2.67E-01	0.76	1.35E-02
Hoffmann	rs2404715	1	57008778	SBP	C	0.40	1.70E-09	0.43	-0.04	0.18	5.02E-01	9.48E-01	6.83E-01	1.00E+00	1.00	1.00E+00	0.18	6.83E-01
Hoffmann	rs2404715	1	57008778	PP	C	0.34	2.70E-14	0.09	0.07	0.08	8.23E-01	8.53E-01	7.75E-01	1.00E+00	1.00	1.00E+00	0.08	7.75E-01

Table S8: Generalization of previously reported loci associated with quantitative BP traits.

Ref	rsID	chr	position	trait	A1	discovery		HCHS/SOL beta			HCHS/SOL p-value			FDR$_g$ r-value			all meta	
						beta	p-value	Main	Carib	All	Main	Carib	All	Main	Carib	All	beta	p-value
Hoffmann	rs60199046	1	59663341	SBP	A	0.24	2.60E-08	0.22	0.41	0.30	5.60E-01	3.50E-01	2.95E-01	1.00E+00	0.95	7.07E-01	0.30	2.95E-01
Hoffmann	rs60199046	1	59663341	PP	A	0.31	1.30E-27	-0.06	0.63	0.24	8.05E-01	2.45E-02	1.93E-01	1.00E+00	0.44	5.81E-01	0.24	1.93E-01
Hoffmann	rs786919	1	89281529	SBP	A	0.26	1.30E-11	-0.08	0.56	0.19	7.81E-01	9.21E-02	3.89E-01	1.00E+00	0.66	7.88E-01	0.19	3.89E-01
Hoffmann	rs786919	1	89281529	DBP	A	0.11	4.20E-06	0.10	0.32	0.19	5.63E-01	1.21E-01	1.51E-01	1.00E+00	0.78	5.47E-01	0.19	1.51E-01
Hoffmann	rs786919	1	89281529	PP	A	0.16	1.50E-09	-0.15	0.25	0.02	4.00E-01	2.50E-01	9.13E-01	1.00E+00	0.83	1.00E+00	0.02	9.13E-01
HMG	rs10745332	1	113189053	DBP	A	0.53	7.70E-08	0.51	0.64	0.58	5.72E-02	1.24E-02	1.85E-03	1.55E-01	0.08	5.86E-03	0.54	8.15E-10
HMG	rs10745332	1	113189053	SBP	A	0.96	2.52E-09	0.74	1.23	1.00	8.79E-02	2.97E-03	9.16E-04	2.88E-01	0.06	4.35E-03	0.97	3.41E-10
Ehret '11	rs2932538	1	113216543	DBP	G	0.24	9.90E-10	0.51	0.72	0.62	5.93E-02	6.19E-03	1.07E-03	1.43E-01	0.03	4.62E-03	0.26	2.99E-11
Ehret '11	rs2932538	1	113216543	SBP	G	0.39	1.20E-09	0.69	1.33	1.02	1.12E-01	1.68E-03	8.04E-04	3.36E-01	0.04	7.77E-03	0.41	3.20E-11
Hoffmann	rs11102916	1	115836746	SBP	C	-0.73	1.10E-07	-0.05	-1.64	-0.71	9.39E-01	5.36E-02	1.93E-01	1.00E+00	0.56	6.58E-01	-0.71	1.93E-01
Hoffmann	rs11102916	1	115836746	DBP	C	-0.57	9.50E-12	-0.74	-0.60	-0.68	9.88E-02	2.59E-01	4.68E-02	5.49E-01	0.87	3.11E-01	-0.68	4.68E-02
Liu	rs35479618	1	153662423	SBP	A	1.11	5.70E-08	1.71	1.55	1.60	4.96E-01	3.58E-01	2.54E-01	7.14E-01	0.72	4.77E-01	1.12	1.55E-08
Hoffmann	rs7519279	1	169207361	SBP	G	0.19	5.00E-06	0.81	0.60	0.70	3.68E-02	1.32E-01	1.18E-02	4.17E-01	0.68	2.51E-01	0.70	1.18E-02
Hoffmann	rs7519279	1	169207361	PP	G	0.21	1.10E-13	0.64	0.42	0.53	1.18E-02	1.05E-01	3.74E-03	5.44E-01	0.66	1.91E-01	0.53	3.74E-03
Hoffmann	rs12405515	1	172357441	SBP	G	0.19	1.20E-06	0.39	-0.11	0.18	1.89E-01	7.44E-01	4.36E-01	8.13E-01	1.00	8.20E-01	0.18	4.36E-01
Hoffmann	rs12405515	1	172357441	DBP	G	0.17	9.70E-13	0.30	-0.11	0.12	1.05E-01	5.97E-01	3.82E-01	5.49E-01	1.00	8.02E-01	0.12	3.82E-01
Hoffmann	rs567058829	1	175111760	DBP	C	0.20	3.50E-08	0.15	-0.38	-0.09	4.69E-01	8.80E-02	5.36E-01	1.00E+00	1.00	1.00E+00	-0.09	5.36E-01
Hoffmann	rs61823001	1	176664440	PP	A	0.31	4.40E-08	0.78	-0.08	0.33	1.79E-01	8.83E-01	4.13E-01	7.37E-01	1.00	8.71E-01	0.33	4.13E-01
Hoffmann	rs12037669	1	201721930	PP	T	0.22	1.40E-10	-0.19	0.31	-0.01	3.41E-01	2.43E-01	9.56E-01	1.00E+00	0.83	1.00E+00	-0.01	9.56E-01
Hoffmann	rs2761436	1	207919748	SBP	C	-0.20	2.10E-07	0.11	-0.28	-0.05	6.85E-01	4.01E-01	8.27E-01	1.00E+00	1.00	1.00E+00	-0.05	8.27E-01
Hoffmann	rs2761436	1	207919748	PP	C	-0.15	2.10E-09	0.12	-0.20	-0.01	5.16E-01	3.45E-01	9.17E-01	1.00E+00	0.94	1.00E+00	-0.01	9.17E-01
Hoffmann	rs17046596	1	217722449	DBP	A	-0.15	1.50E-08	-0.66	-0.33	-0.55	1.66E-04	1.75E-01	1.31E-04	2.55E-02	0.83	2.00E-02	-0.55	1.31E-04
Hoffmann	rs4653889	1	228112121	SBP	A	0.20	1.20E-07	0.22	-0.19	0.06	4.39E-01	5.88E-01	7.94E-01	1.00E+00	1.00	1.00E+00	0.06	7.94E-01

Table S8: Generalization of previously reported loci associated with quantitative BP traits.

Ref	rsID	chr	position	trait	A1	discovery		HCHS/SOL beta			HCHS/SOL p-value			FDR$_g$ r-value			all meta	
						beta	p-value	Main	Carib	All	Main	Carib	All	Main	Carib	All	beta	p-value
Hoffmann	rs4653889	1	228112121	DBP	A	0.15	1.20E-10	0.13	0.12	0.13	4.52E-01	5.60E-01	3.44E-01	1.00E+00	1.00	7.82E-01	0.13	3.44E-01
Hoffmann	rs6428947	1	236326005	SBP	C	0.25	5.20E-07	0.35	0.75	0.49	2.29E-01	6.38E-02	4.08E-02	8.26E-01	0.58	3.67E-01	0.49	4.08E-02
Hoffmann	rs6428947	1	236326005	DBP	C	0.20	3.20E-11	0.45	0.26	0.38	1.45E-02	3.12E-01	1.04E-02	3.26E-01	0.90	2.07E-01	0.38	1.04E-02
Hoffmann	rs6429422	1	243472801	DBP	T	-0.25	1.50E-23	0.09	-0.10	0.00	6.60E-01	6.42E-01	9.95E-01	1.00E+00	1.00	1.00E+00	0.00	9.95E-01
Hoffmann	rs7586597	2	9300092	PP	G	-0.15	7.00E-09	0.18	0.03	0.11	3.91E-01	8.88E-01	4.68E-01	1.00E+00	1.00	1.00E+00	0.11	4.68E-01
Kato	rs1344653	2	19730845	PP	A	0.27	7.80E-12	-0.05	-0.20	-0.12	7.85E-01	3.54E-01	4.18E-01	1.00E+00	1.00	1.00E+00	0.24	3.21E-10
Hoffmann	rs7255	2	20878820	PP	T	-0.20	2.10E-15	-0.14	-0.75	-0.39	4.38E-01	4.82E-04	4.67E-03	1.00E+00	0.05	1.91E-01	-0.39	4.67E-03
Hoffmann	rs10198275	2	25130542	SBP	A	0.17	7.50E-06	0.36	-0.27	0.07	2.59E-01	4.27E-01	7.71E-01	8.47E-01	1.00	1.00E+00	0.07	7.71E-01
Hoffmann	rs10198275	2	25130542	DBP	A	0.14	5.90E-09	0.16	0.00	0.09	4.12E-01	9.87E-01	5.47E-01	1.00E+00	1.00	1.00E+00	0.09	5.47E-01
Kato	rs1275988	2	26914364	MAP	T	0.37	5.00E-21	-0.51	-0.47	-0.49	1.91E-02	5.41E-02	2.75E-03	1.00E+00	1.00	1.00E+00	0.32	1.25E-16
Hoffmann	rs13420463	2	37517566	SBP	A	0.28	1.40E-10	0.91	0.56	0.77	1.63E-03	1.11E-01	6.75E-04	2.78E-01	0.66	5.74E-02	0.77	6.75E-04
Hoffmann	rs13420463	2	37517566	DBP	A	0.13	1.20E-06	0.53	0.29	0.43	3.57E-03	1.78E-01	2.23E-03	1.82E-01	0.83	8.51E-02	0.43	2.23E-03
Hoffmann	rs13420463	2	37517566	PP	A	0.16	4.90E-08	0.38	0.26	0.33	4.40E-02	2.50E-01	2.37E-02	5.44E-01	0.83	3.10E-01	0.33	2.37E-02
Hoffmann	rs13403122	2	43078758	PP	C	0.19	7.20E-11	0.10	0.72	0.39	6.76E-01	6.99E-03	3.32E-02	1.00E+00	0.28	3.10E-01	0.19	1.28E-11
Hoffmann	rs13403122	2	43078758	SBP	C	0.25	8.90E-09	0.15	1.30	0.68	6.90E-01	1.57E-03	1.55E-02	1.00E+00	0.27	2.51E-01	0.68	1.55E-02
Hoffmann	rs13403122	2	43078758	DBP	C	0.20	1.90E-13	0.05	0.59	0.30	8.18E-01	2.13E-02	8.46E-02	1.00E+00	0.61	4.15E-01	0.30	8.46E-02
Hoffmann	rs2115859	2	43386092	PP	T	0.16	4.10E-09	0.04	0.42	0.19	8.26E-01	6.99E-02	1.94E-01	1.00E+00	0.53	5.81E-01	0.16	1.76E-09
Hoffmann	rs11690961	2	46363336	PP	A	0.31	1.20E-14	0.01	-0.32	-0.15	9.82E-01	4.69E-01	6.28E-01	1.00E+00	1.00	1.00E+00	-0.15	6.28E-01
Hoffmann	rs10199082	2	56040099	PP	T	0.26	1.70E-11	-0.31	0.47	0.06	4.12E-01	2.37E-01	8.32E-01	1.00E+00	0.83	1.00E+00	0.06	8.32E-01
Hoffmann	rs7575523	2	59335104	PP	T	0.14	4.80E-08	-0.16	0.12	-0.04	4.19E-01	5.95E-01	7.90E-01	1.00E+00	1.00	1.00E+00	-0.04	7.90E-01
Hoffmann	rs2540950	2	65279223	SBP	C	0.19	8.90E-07	0.51	0.13	0.35	8.24E-02	7.10E-01	1.20E-01	5.63E-01	1.00	6.36E-01	0.35	1.20E-01
Hoffmann	rs2540950	2	65279223	PP	C	0.17	6.30E-11	0.12	0.41	0.24	5.35E-01	6.43E-02	9.58E-02	1.00E+00	0.53	4.65E-01	0.24	9.58E-02
Hoffmann	rs7605066	2	71529331	PP	C	0.15	6.30E-09	-0.16	0.37	0.06	4.02E-01	9.94E-02	6.81E-01	1.00E+00	0.66	1.00E+00	0.06	6.81E-01

Table S8: Generalization of previously reported loci associated with quantitative BP traits.

Ref	rsID	chr	position	trait	A1	discovery		HCHS/SOL beta			HCHS/SOL p-value			FDR_g r-value			all meta	
						beta	p-value	Main	Carib	All	Main	Carib	All	Main	Carib	All	beta	p-value
Hoffmann	rs1876487	2	73114352	SBP	A	-0.20	3.60E-06	-0.35	-0.41	-0.37	2.33E-01	2.62E-01	1.05E-01	8.26E-01	0.89	6.36E-01	-0.37	1.05E-01
Hoffmann	rs1876487	2	73114352	DBP	A	-0.16	1.50E-09	-0.20	-0.30	-0.24	2.80E-01	1.78E-01	9.42E-02	9.30E-01	0.83	4.34E-01	-0.24	9.42E-02
Hoffmann	rs62162674	2	85502236	PP	G	0.18	1.50E-12	0.13	0.06	0.10	5.09E-01	8.03E-01	5.09E-01	1.00E+00	1.00	9.48E-01	0.10	5.09E-01
Hoffmann	rs3731818	2	86368804	SBP	G	0.24	4.10E-09	-0.08	0.46	0.10	7.68E-01	2.31E-01	6.47E-01	1.00E+00	0.87	1.00E+00	0.10	6.47E-01
Hoffmann	rs3731818	2	86368804	DBP	G	0.12	2.80E-06	-0.02	0.41	0.13	9.27E-01	8.87E-02	3.56E-01	1.00E+00	0.68	7.88E-01	0.13	3.56E-01
Hoffmann	rs3731818	2	86368804	PP	G	0.13	3.50E-06	-0.09	0.06	-0.04	6.43E-01	8.23E-01	8.08E-01	1.00E+00	1.00	1.00E+00	-0.04	8.08E-01
Hoffmann	rs6747874	2	101578489	PP	G	-0.17	1.20E-08	-0.34	-0.30	-0.32	7.43E-02	2.26E-01	3.27E-02	5.60E-01	0.83	3.10E-01	-0.32	3.27E-02
Liu	rs6722745	2	108875244	SBP	C	0.27	1.10E-07	0.06	0.10	0.08	8.26E-01	7.66E-01	7.25E-01	9.44E-01	1.00	7.25E-01	0.26	8.97E-08
Hoffmann	rs3923097	2	124020790	DBP	T	0.27	1.40E-08	-0.01	0.38	0.09	9.79E-01	3.77E-01	6.81E-01	1.00E+00	0.95	1.00E+00	0.09	6.81E-01
Hoffmann	rs58117425	2	145681570	DBP	G	-0.19	8.30E-12	-0.38	-0.86	-0.54	9.03E-02	7.86E-03	3.79E-03	5.49E-01	0.58	1.16E-01	-0.54	3.79E-03
HMG	rs16849225	2	164906820	DBP	C	0.10	2.07E-01	0.05	0.07	0.06	8.16E-01	8.02E-01	7.36E-01	1.00E+00	1.00	1.00E+00	0.09	2.02E-01
HMG	rs16849225	2	164906820	SBP	C	0.45	1.03E-03	0.81	0.05	0.51	2.02E-02	9.05E-01	6.22E-02	1.00E+00	1.00	1.00E+00	0.46	2.06E-04
Hoffmann	rs13024657	2	175472839	SBP	C	-0.30	3.60E-08	0.47	-0.10	0.20	3.24E-01	8.39E-01	5.69E-01	1.00E+00	1.00	1.00E+00	0.20	5.69E-01
Hoffmann	rs13024657	2	175472839	PP	C	-0.22	1.90E-09	0.04	-0.26	-0.10	8.99E-01	4.21E-01	6.44E-01	1.00E+00	1.00	1.00E+00	-0.10	6.44E-01
Hoffmann	rs4972805	2	177012570	SBP	C	0.18	8.90E-06	-0.09	-0.21	-0.13	7.72E-01	5.76E-01	5.74E-01	1.00E+00	1.00	1.00E+00	-0.13	5.74E-01
Hoffmann	rs4972805	2	177012570	DBP	C	0.14	1.80E-08	-0.00	-0.19	-0.08	9.93E-01	4.03E-01	6.01E-01	1.00E+00	1.00	1.00E+00	-0.08	6.01E-01
Hoffmann	rs2706110	2	178092162	PP	T	0.18	9.30E-09	-0.04	-0.01	-0.03	8.57E-01	9.74E-01	8.82E-01	1.00E+00	1.00	1.00E+00	-0.03	8.82E-01
Hoffmann	rs7591091	2	179754194	DBP	T	0.13	2.10E-07	0.15	0.21	0.18	4.17E-01	3.18E-01	2.01E-01	1.00E+00	0.90	6.02E-01	0.18	2.01E-01
Levy	rs11895934	2	190802253	SBP	C	0.96	7.30E-07	-0.17	0.30	0.09	7.04E-01	4.66E-01	7.80E-01	1.00E+00	0.54	9.10E-01	0.72	9.00E-06
Levy	rs7571613	2	190805662	SBP	G	0.96	7.20E-07	-0.19	0.42	0.14	6.79E-01	3.15E-01	6.47E-01	1.00E+00	0.41	7.99E-01	0.73	5.62E-06
Levy	rs7564968	2	190811972	SBP	C	0.96	8.00E-07	-0.30	0.24	-0.02	5.11E-01	5.81E-01	9.60E-01	1.00E+00	0.64	1.00E+00	0.70	1.70E-05
Levy	rs13401889	2	190910559	DBP	C	0.54	9.70E-07	0.42	-0.04	0.21	4.16E-02	8.43E-01	1.73E-01	1.31E-01	1.00	2.09E-01	0.43	1.70E-06
Hoffmann	rs6434404	2	191494411	SBP	A	0.31	4.90E-13	0.57	0.38	0.48	1.47E-01	3.69E-01	9.70E-02	7.15E-01	0.97	6.36E-01	0.48	9.70E-02

Table S8: Generalization of previously reported loci associated with quantitative BP traits.

Ref	rsID	chr	position	trait	A1	discovery		HCHS/SOL beta			HCHS/SOL p-value			FDR$_g$ r-value			all meta	
						beta	p-value	Main	Carib	All	Main	Carib	All	Main	Carib	All	beta	p-value
Hoffmann	rs6434404	2	191494411	DBP	A	0.12	1.80E-06	0.32	0.17	0.25	2.03E-01	5.09E-01	1.72E-01	7.98E-01	1.00	5.89E-01	0.25	1.72E-01
Hoffmann	rs6434404	2	191494411	PP	A	0.18	1.30E-10	0.28	0.23	0.25	2.87E-01	4.03E-01	1.79E-01	9.46E-01	1.00	5.78E-01	0.25	1.79E-01
Hoffmann	rs114407963	2	204154677	DBP	A	0.16	7.30E-06	0.14	0.10	0.12	6.69E-01	7.67E-01	6.08E-01	1.00E+00	1.00	1.00E+00	0.12	6.08E-01
Hoffmann	rs114407963	2	204154677	PP	A	-0.23	2.50E-09	-0.55	-0.01	-0.29	1.09E-01	9.80E-01	2.47E-01	5.84E-01	1.00	6.88E-01	-0.29	2.47E-01
Hoffmann	rs2360970	2	208409339	SBP	G	-0.21	1.80E-08	-0.07	-0.28	-0.15	8.08E-01	4.26E-01	5.04E-01	1.00E+00	1.00	8.65E-01	-0.15	5.04E-01
Hoffmann	rs2360970	2	208409339	DBP	G	-0.12	1.20E-07	0.19	-0.12	0.07	2.85E-01	5.82E-01	6.22E-01	1.00E+00	1.00	1.00E+00	0.07	6.22E-01
Hoffmann	rs1250247	2	216299629	SBP	C	0.31	1.10E-12	0.45	0.01	0.28	1.74E-01	9.85E-01	2.82E-01	8.13E-01	1.00	6.95E-01	0.28	2.82E-01
Hoffmann	rs1250247	2	216299629	PP	C	0.28	1.10E-21	0.43	-0.13	0.21	4.84E-02	6.29E-01	2.13E-01	5.44E-01	1.00	6.11E-01	0.21	2.13E-01
Hoffmann	rs72958213	2	217646523	PP	C	0.18	4.50E-08	0.09	-0.27	-0.05	6.95E-01	3.36E-01	7.78E-01	1.00E+00	1.00	1.00E+00	-0.05	7.78E-01
Hoffmann	rs1063281	2	218668732	SBP	C	0.25	2.30E-10	-0.11	-0.23	-0.16	6.92E-01	4.92E-01	4.57E-01	1.00E+00	1.00	1.00E+00	-0.16	4.57E-01
Hoffmann	rs1063281	2	218668732	DBP	C	0.17	9.50E-13	-0.00	-0.23	-0.10	9.97E-01	2.84E-01	4.87E-01	1.00E+00	1.00	1.00E+00	-0.10	4.87E-01
Hoffmann	rs7590201	2	227192443	SBP	G	-0.22	3.00E-08	0.84	-0.19	0.42	3.83E-03	5.89E-01	6.01E-02	1.00E+00	1.00	1.00E+00	0.42	6.01E-02
Hoffmann	rs7590201	2	227192443	DBP	G	-0.14	7.60E-09	0.51	-0.20	0.22	5.03E-03	3.58E-01	1.19E-01	1.00E+00	0.95	1.00E+00	0.22	1.19E-01
Hoffmann	rs11677932	2	238223955	PP	G	0.15	4.50E-08	-0.04	0.00	-0.02	8.50E-01	9.89E-01	8.94E-01	1.00E+00	1.00	1.00E+00	-0.02	8.94E-01
Hoffmann	rs74621754	3	8496371	PP	G	0.36	2.70E-08	-0.70	-1.09	-0.91	3.81E-01	1.38E-01	9.22E-02	1.00E+00	1.00	1.00E+00	-0.91	9.22E-02
Hoffmann	rs6793656	3	13823342	PP	A	-0.22	5.70E-10	-0.09	-0.04	-0.06	7.81E-01	9.10E-01	7.85E-01	1.00E+00	1.00	1.00E+00	-0.06	7.85E-01
Hoffmann	rs12630213	3	14954411	SBP	C	0.28	6.30E-12	0.76	0.12	0.50	8.45E-03	7.24E-01	2.45E-02	3.46E-01	1.00	3.02E-01	0.50	2.45E-02
Hoffmann	rs12630213	3	14954411	DBP	C	0.15	4.80E-10	0.44	0.12	0.31	1.49E-02	5.78E-01	2.63E-02	3.26E-01	1.00	2.88E-01	0.31	2.63E-02
Ehret '16	rs11128722	3	14958126	SBP	A	-0.31	3.61E-11	-0.47	0.04	-0.27	8.68E-02	9.15E-01	2.09E-01	3.36E-01	1.00	5.86E-01	-0.31	1.68E-11
Ehret '16	rs11128722	3	14958126	DBP	A	-0.17	5.16E-10	-0.34	-0.07	-0.23	4.93E-02	7.46E-01	8.66E-02	6.41E-01	1.00	4.71E-01	-0.18	1.30E-10
Ehret '11	rs13082711	3	27537909	DBP	T	-0.24	3.80E-09	-0.33	-0.29	-0.31	1.44E-01	3.14E-01	7.64E-02	2.82E-01	0.51	1.48E-01	-0.24	8.25E-10
Ehret '11	rs13082711	3	27537909	SBP	T	-0.32	1.50E-06	-0.44	-0.25	-0.37	2.17E-01	5.83E-01	1.89E-01	3.94E-01	0.99	3.43E-01	-0.32	6.24E-07
HMG	rs820430	3	27548900	DBP	A	0.27	7.57E-06	0.09	0.27	0.16	6.15E-01	2.43E-01	2.66E-01	9.74E-01	0.42	4.59E-01	0.25	4.65E-06

Table S8: Generalization of previously reported loci associated with quantitative BP traits.

Ref	rsID	chr	position	trait	A1	discovery		HCHS/SOL beta			HCHS/SOL p-value			FDR$_g$ r-value			all meta	
						beta	p-value	Main	Carib	All	Main	Carib	All	Main	Carib	All	beta	p-value
HMG	rs820430	3	27548900	SBP	A	0.76	1.36E-12	0.48	0.80	0.60	9.72E-02	3.32E-02	9.24E-03	2.88E-01	0.16	2.93E-02	0.73	1.90E-13
Levy	rs6768438	3	41865355	DBP	A	0.59	9.70E-07	0.70	0.42	0.55	4.05E-03	7.38E-02	1.16E-03	9.13E-02	0.13	2.69E-02	0.58	3.87E-09
Hoffmann	rs149240564	3	41867621	PP	A	0.60	3.90E-35	0.20	0.31	0.26	4.66E-01	2.23E-01	1.67E-01	1.00E+00	0.83	5.71E-01	0.58	7.24E-35
Hoffmann	rs9816560	3	41872527	PP	G	0.43	9.80E-20	-0.27	0.41	0.18	5.77E-01	2.34E-01	5.26E-01	1.00E+00	0.83	9.48E-01	0.42	1.16E-19
Levy	rs9816772	3	41872877	DBP	T	0.59	9.70E-07	0.49	0.34	0.41	4.91E-02	1.54E-01	1.74E-02	1.31E-01	0.23	3.04E-02	0.53	6.73E-08
Levy	rs9852991	3	41875455	DBP	A	0.59	9.70E-07	0.33	0.27	0.30	2.23E-01	3.11E-01	1.16E-01	3.00E-01	0.39	1.50E-01	0.51	5.91E-07
Ehret '11	rs3774372	3	41877414	DBP	T	-0.37	9.00E-14	-0.34	-0.29	-0.31	2.13E-01	2.72E-01	9.87E-02	3.25E-01	0.46	1.79E-01	-0.36	2.34E-14
Ehret '11	rs3774372	3	41877414	SBP	T	-0.07	3.90E-01	-0.02	0.07	0.03	9.66E-01	8.65E-01	9.30E-01	1.00E+00	1.00	1.00E+00	-0.06	4.17E-01
HMG	rs9815354	3	41912651	DBP	A	0.43	1.34E-05	0.25	0.28	0.27	3.54E-01	2.92E-01	1.62E-01	6.73E-01	0.46	3.86E-01	0.39	8.30E-06
HMG	rs9815354	3	41912651	SBP	A	0.10	5.47E-01	-0.11	-0.02	-0.06	8.07E-01	9.65E-01	8.42E-01	1.00E+00	1.00	1.00E+00	0.06	7.05E-01
Levy	rs9815354	3	41912651	DBP	A	0.60	7.80E-07	0.25	0.28	0.27	3.54E-01	2.92E-01	1.62E-01	4.59E-01	0.38	2.03E-01	0.51	6.46E-07
Franceschini	rs1717027	3	41987920	SBP	T	0.18	3.00E-01	0.34	0.02	0.18	3.79E-01	9.58E-01	5.16E-01	1.00E+00	1.00	1.00E+00	0.18	2.21E-01
Franceschini	rs1717027	3	41987920	DBP	T	0.49	4.60E-13	0.65	0.40	0.52	7.19E-03	8.42E-02	2.12E-03	5.03E-02	0.59	1.48E-02	0.49	3.82E-15
Hoffmann	rs76398786	3	48731450	DBP	C	-0.38	1.50E-08	-1.06	-1.21	-1.13	1.02E-01	6.61E-02	1.41E-02	5.49E-01	0.61	2.16E-01	-1.13	1.41E-02
Hoffmann	rs3749237	3	49770032	SBP	G	-0.21	4.00E-07	0.36	-0.41	-0.00	3.24E-01	2.85E-01	9.91E-01	1.00E+00	0.89	1.00E+00	-0.00	9.91E-01
Hoffmann	rs3749237	3	49770032	DBP	G	-0.17	3.50E-11	0.23	-0.31	-0.02	3.11E-01	2.01E-01	8.89E-01	1.00E+00	0.85	1.00E+00	-0.02	8.89E-01
Hoffmann	rs2236973	3	50474284	SBP	T	-0.26	3.10E-06	0.11	-0.46	-0.10	7.11E-01	2.24E-01	6.57E-01	1.00E+00	0.87	1.00E+00	-0.10	6.57E-01
Hoffmann	rs2236973	3	50474284	DBP	T	-0.18	4.90E-08	0.09	-0.19	-0.02	6.21E-01	4.11E-01	9.15E-01	1.00E+00	0.99	1.00E+00	-0.02	9.15E-01
Hoffmann	rs528266117	3	52729780	SBP	C	-24.45	7.40E-09	-0.46	1.95	1.05	9.40E-01	6.79E-01	7.78E-01	1.00E+00	1.00	1.00E+00	1.05	7.78E-01
Hoffmann	rs528266117	3	52729780	PP	C	-15.80	1.40E-08	-0.30	2.23	1.30	9.40E-01	4.61E-01	5.90E-01	1.00E+00	1.00	1.00E+00	1.30	5.90E-01
HMG	rs9810888	3	53635595	DBP	G	0.39	4.00E-12	0.09	0.27	0.17	5.99E-01	1.90E-01	2.13E-01	9.74E-01	0.42	4.38E-01	0.35	1.16E-10
HMG	rs9810888	3	53635595	SBP	G	0.53	5.46E-08	0.22	0.57	0.37	4.39E-01	8.62E-02	9.14E-02	7.57E-01	0.27	2.17E-01	0.50	3.41E-08
Hoffmann	rs9845655	3	56701328	SBP	T	0.21	3.30E-07	0.54	0.28	0.44	5.89E-02	4.32E-01	5.20E-02	5.44E-01	1.00	4.02E-01	0.44	5.20E-02

Table S8: Generalization of previously reported loci associated with quantitative BP traits.

Ref	rsID	chr	position	trait	A1	discovery		HCHS/SOL beta			HCHS/SOL p-value			FDR$_g$ r-value			all meta	
						beta	p-value	Main	Carib	All	Main	Carib	All	Main	Carib	All	beta	p-value
Hoffmann	rs9845655	3	56701328	DBP	T	0.16	5.00E-10	0.18	0.23	0.20	3.02E-01	2.97E-01	1.47E-01	9.30E-01	0.90	5.47E-01	0.20	1.47E-01
Hoffmann	rs1053711	3	57743246	DBP	G	0.15	2.20E-09	-0.28	0.12	-0.08	2.70E-01	6.44E-01	6.57E-01	1.00E+00	1.00	1.00E+00	-0.08	6.57E-01
Hoffmann	rs6795735	3	64705365	DBP	C	0.16	1.30E-11	0.09	-0.07	0.01	6.78E-01	7.40E-01	9.34E-01	1.00E+00	1.00	1.00E+00	0.01	9.34E-01
Hoffmann	rs17831815	3	66437086	SBP	T	0.20	3.90E-07	-0.12	-0.18	-0.14	6.78E-01	6.27E-01	5.31E-01	1.00E+00	1.00	1.00E+00	-0.14	5.31E-01
Hoffmann	rs17831815	3	66437086	PP	T	0.18	4.70E-11	-0.07	0.22	0.05	7.27E-01	3.53E-01	7.45E-01	1.00E+00	0.94	1.00E+00	0.05	7.45E-01
Hoffmann	rs12636552	3	70972466	SBP	A	0.24	4.80E-09	0.09	0.41	0.24	7.72E-01	2.25E-01	3.01E-01	1.00E+00	0.87	7.10E-01	0.24	3.01E-01
Hoffmann	rs12636552	3	70972466	PP	A	0.14	1.30E-07	0.03	0.11	0.07	8.75E-01	6.04E-01	6.40E-01	1.00E+00	1.00	1.00E+00	0.07	6.40E-01
Hoffmann	rs6803322	3	84986088	SBP	C	0.23	4.60E-08	0.42	0.22	0.33	2.69E-01	5.82E-01	2.41E-01	8.47E-01	1.00	6.59E-01	0.33	2.41E-01
Hoffmann	rs6803322	3	84986088	PP	C	0.13	3.20E-06	0.20	0.02	0.12	4.09E-01	9.43E-01	5.19E-01	1.00E+00	1.00	9.48E-01	0.12	5.19E-01
Hoffmann	rs76217164	3	88738041	SBP	T	2.21	3.90E-06	0.09	-1.38	-1.07	9.71E-01	2.62E-01	3.27E-01	1.00E+00	1.00	1.00E+00	-1.07	3.27E-01
Hoffmann	rs76217164	3	88738041	DBP	T	1.63	1.30E-07	0.67	-1.21	-0.82	6.53E-01	1.12E-01	2.29E-01	1.00E+00	1.00	1.00E+00	-0.82	2.29E-01
Hoffmann	rs9882772	3	122110149	DBP	T	-0.13	1.50E-08	-0.32	0.00	-0.18	1.11E-01	9.90E-01	2.41E-01	5.49E-01	1.00	6.36E-01	-0.18	2.41E-01
Hoffmann	rs4141663	3	124551967	SBP	C	0.26	1.70E-11	-0.59	0.62	-0.08	3.93E-02	6.55E-02	7.06E-01	1.00E+00	0.58	1.00E+00	-0.08	7.06E-01
Hoffmann	rs4141663	3	124551967	DBP	C	0.13	7.30E-08	-0.07	0.32	0.10	7.10E-01	1.22E-01	4.72E-01	1.00E+00	0.78	9.03E-01	0.10	4.72E-01
Hoffmann	rs4141663	3	124551967	PP	C	0.12	1.10E-06	-0.54	0.31	-0.18	4.30E-03	1.56E-01	2.20E-01	1.00E+00	0.75	1.00E+00	-0.18	2.20E-01
Hoffmann	rs62270945	3	128201889	SBP	C	-0.61	1.10E-06	-0.73	0.29	-0.20	6.55E-01	8.55E-01	8.59E-01	1.00E+00	1.00	1.00E+00	-0.20	8.59E-01
Hoffmann	rs62270945	3	128201889	PP	C	-0.53	1.70E-10	-0.01	0.21	0.10	9.90E-01	8.38E-01	8.91E-01	1.00E+00	1.00	1.00E+00	0.10	8.91E-01
Hoffmann	rs75305034	3	133886705	SBP	T	0.33	3.40E-10	0.26	0.03	0.16	4.55E-01	9.46E-01	5.45E-01	1.00E+00	1.00	8.99E-01	0.16	5.45E-01
Hoffmann	rs75305034	3	133886705	DBP	T	0.26	7.00E-14	0.30	0.14	0.23	1.61E-01	5.60E-01	1.54E-01	6.65E-01	1.00	5.47E-01	0.23	1.54E-01
Hoffmann	rs9864898	3	138111751	SBP	C	-0.27	3.50E-07	-0.51	0.52	-0.04	3.40E-01	3.78E-01	9.20E-01	9.49E-01	1.00	1.00E+00	-0.04	9.20E-01
Hoffmann	rs9864898	3	138111751	DBP	C	-0.19	4.60E-09	-0.50	-0.01	-0.27	1.38E-01	9.83E-01	2.72E-01	6.21E-01	1.00	6.72E-01	-0.27	2.72E-01
Hoffmann	rs6782694	3	141627860	PP	C	-0.19	2.10E-08	-0.06	0.63	0.22	7.92E-01	3.03E-02	2.39E-01	1.00E+00	1.00	1.00E+00	0.22	2.39E-01
Hoffmann	rs11708647	3	142617353	PP	G	0.18	4.40E-11	0.28	0.14	0.22	2.10E-01	5.66E-01	1.90E-01	8.01E-01	1.00	5.81E-01	0.22	1.90E-01

Table S8: Generalization of previously reported loci associated with quantitative BP traits.

Ref	rsID	chr	position	trait	A1	discovery		HCHS/SOL beta			HCHS/SOL p-value			FDR$_g$ r-value			all meta	
						beta	p-value	Main	Carib	All	Main	Carib	All	Main	Carib	All	beta	p-value
Hoffmann	rs9844972	3	150097635	SBP	G	-0.44	8.90E-09	0.28	-1.27	-0.49	7.24E-01	1.13E-01	3.86E-01	1.00E+00	0.66	7.88E-01	-0.49	3.86E-01
Hoffmann	rs9844972	3	150097635	PP	G	-0.30	7.40E-09	-0.05	-1.04	-0.55	9.26E-01	4.42E-02	1.36E-01	1.00E+00	0.44	5.06E-01	-0.55	1.36E-01
Hoffmann	rs113161639	3	154615819	SBP	G	0.45	3.90E-12	0.47	1.65	0.97	4.34E-01	1.69E-02	3.18E-02	1.00E+00	0.47	3.60E-01	0.97	3.18E-02
Hoffmann	rs113161639	3	154615819	DBP	G	0.29	3.20E-13	0.98	0.34	0.70	8.54E-03	4.33E-01	1.27E-02	3.26E-01	1.00	2.16E-01	0.70	1.27E-02
Hoffmann	rs2178452	3	160370160	SBP	G	0.26	1.10E-10	0.23	0.09	0.17	4.30E-01	8.11E-01	4.48E-01	1.00E+00	1.00	8.28E-01	0.17	4.48E-01
Hoffmann	rs2178452	3	160370160	DBP	G	0.13	5.70E-08	-0.03	-0.01	-0.02	8.84E-01	9.66E-01	8.92E-01	1.00E+00	1.00	1.00E+00	-0.02	8.92E-01
Hoffmann	rs2178452	3	160370160	PP	G	0.14	2.90E-07	0.25	0.10	0.19	1.94E-01	6.49E-01	1.98E-01	7.53E-01	1.00	5.86E-01	0.19	1.98E-01
Ehret ’11	rs419076	3	169100886	DBP	T	0.24	2.10E-12	0.39	0.25	0.33	4.24E-02	2.35E-01	2.26E-02	1.23E-01	0.45	5.57E-02	0.25	1.76E-13
Ehret ’11	rs419076	3	169100886	SBP	T	0.41	1.80E-13	0.63	0.19	0.43	4.22E-02	5.71E-01	6.24E-02	1.75E-01	0.99	1.51E-01	0.41	3.09E-14
Hoffmann	rs4686683	3	185307363	SBP	T	-0.18	2.50E-06	0.44	-0.52	0.05	1.12E-01	1.21E-01	8.06E-01	1.00E+00	0.68	1.00E+00	0.05	8.06E-01
Hoffmann	rs4686683	3	185307363	DBP	T	-0.17	3.80E-13	0.02	-0.43	-0.16	9.00E-01	4.01E-02	2.27E-01	1.00E+00	0.61	6.23E-01	-0.16	2.27E-01
Hoffmann	rs2498323	4	3451109	PP	G	-0.29	1.10E-09	-0.44	-0.85	-0.67	2.28E-01	1.22E-02	8.07E-03	8.10E-01	0.34	2.28E-01	-0.67	8.07E-03
Ehret ’16	rs2291435	4	38387395	SBP	T	-0.34	1.90E-14	-0.08	-0.62	-0.30	7.68E-01	6.66E-02	1.63E-01	9.98E-01	0.43	5.86E-01	-0.34	6.86E-15
Ehret ’16	rs2291435	4	38387395	DBP	T	-0.16	4.26E-09	0.09	-0.29	-0.06	5.95E-01	1.68E-01	6.31E-01	1.00E+00	0.45	1.00E+00	-0.15	5.00E-09
Hoffmann	rs13104866	4	38402183	SBP	G	0.28	3.80E-13	-0.02	0.67	0.26	9.38E-01	4.48E-02	2.22E-01	1.00E+00	0.56	6.58E-01	0.26	2.22E-01
Hoffmann	rs13104866	4	38402183	DBP	G	0.12	1.60E-07	0.03	0.37	0.17	8.47E-01	7.70E-02	1.99E-01	1.00E+00	0.62	6.02E-01	0.17	1.99E-01
Hoffmann	rs13104866	4	38402183	PP	G	0.15	3.20E-09	-0.07	0.31	0.09	7.00E-01	1.47E-01	5.21E-01	1.00E+00	0.74	9.48E-01	0.09	5.21E-01
Hoffmann	rs17471509	4	48301691	SBP	A	0.19	9.20E-07	-0.33	0.71	0.09	2.36E-01	3.57E-02	6.77E-01	1.00E+00	0.56	1.00E+00	0.09	6.77E-01
Hoffmann	rs17471509	4	48301691	PP	A	0.14	3.50E-08	-0.24	0.29	-0.02	1.98E-01	1.83E-01	8.87E-01	1.00E+00	0.82	1.00E+00	-0.02	8.87E-01
Hoffmann	rs12504699	4	48934298	SBP	G	0.22	2.10E-08	-0.22	0.33	0.00	4.43E-01	3.50E-01	9.92E-01	1.00E+00	0.95	1.00E+00	0.00	9.92E-01
Hoffmann	rs12504699	4	48934298	DBP	G	0.11	5.70E-06	-0.04	0.31	0.11	8.24E-01	1.53E-01	4.56E-01	1.00E+00	0.83	8.83E-01	0.11	4.56E-01
Hoffmann	rs55940751	4	77365891	SBP	C	0.22	3.60E-08	0.26	0.40	0.31	3.70E-01	2.71E-01	1.68E-01	9.55E-01	0.89	6.36E-01	0.31	1.68E-01
Hoffmann	rs55940751	4	77365891	PP	C	0.13	7.50E-07	0.15	0.42	0.26	4.16E-01	7.28E-02	8.00E-02	1.00E+00	0.53	4.40E-01	0.26	8.00E-02

Table S8: Generalization of previously reported loci associated with quantitative BP traits.

Ref	rsID	chr	position	trait	A1	discovery		HCHS/SOL beta			HCHS/SOL p-value			FDR$_g$ r-value			all meta	
						beta	p-value	Main	Carib	All	Main	Carib	All	Main	Carib	All	beta	p-value
HMG	rs1902859	4	81157703	DBP	C	0.71	3.75E-20	0.61	0.59	0.60	1.42E-03	1.71E-02	7.52E-05	1.35E-02	0.08	1.11E-03	0.69	3.11E-22
HMG	rs1902859	4	81157703	SBP	C	1.34	1.76E-22	0.99	0.77	0.91	1.15E-03	5.33E-02	1.87E-04	1.09E-02	0.20	1.78E-03	1.23	3.02E-24
Ehret '11	rs1458038	4	81164723	DBP	T	0.46	8.50E-25	0.78	0.83	0.80	9.81E-05	1.07E-03	4.20E-07	1.52E-03	0.01	1.22E-05	0.48	1.86E-29
Ehret '11	rs1458038	4	81164723	SBP	T	0.71	1.50E-23	1.34	1.24	1.30	2.37E-05	2.57E-03	2.44E-07	6.88E-04	0.04	7.08E-06	0.75	3.01E-28
Kato	rs2014912	4	86715670	SBP	T	0.62	5.40E-17	-0.21	0.34	-0.00	5.38E-01	4.36E-01	9.93E-01	1.00E+00	0.65	1.00E+00	0.57	1.11E-13
Hoffmann	rs57400569	4	89752276	SBP	G	0.24	7.10E-08	0.77	0.02	0.47	2.41E-02	9.67E-01	7.63E-02	3.46E-01	1.00	5.64E-01	0.47	7.63E-02
Hoffmann	rs57400569	4	89752276	PP	G	0.20	1.30E-11	0.50	0.05	0.32	2.69E-02	8.42E-01	6.70E-02	5.44E-01	1.00	4.02E-01	0.32	6.70E-02
Ehret '11	rs13107325	4	103188709	DBP	T	-0.68	2.30E-17	-0.54	-0.14	-0.33	2.42E-01	7.62E-01	3.05E-01	3.51E-01	1.00	4.11E-01	-0.66	2.38E-17
Ehret '11	rs13107325	4	103188709	SBP	T	-0.98	3.30E-14	-0.75	0.27	-0.22	3.15E-01	7.05E-01	6.67E-01	5.07E-01	1.00	8.54E-01	-0.94	8.33E-14
Hoffmann	rs13112725	4	106911742	SBP	G	-0.30	2.10E-11	-0.00	-0.73	-0.28	1.00E+00	4.17E-02	2.09E-01	1.00E+00	0.56	6.58E-01	-0.28	2.09E-01
Hoffmann	rs13112725	4	106911742	DBP	G	-0.15	3.20E-08	-0.15	-0.25	-0.19	4.03E-01	2.50E-01	1.73E-01	1.00E+00	0.87	5.89E-01	-0.19	1.73E-01
Hoffmann	rs13112725	4	106911742	PP	G	-0.14	4.00E-06	0.15	-0.47	-0.09	4.09E-01	4.14E-02	5.26E-01	1.00E+00	0.44	9.48E-01	-0.09	5.26E-01
Hoffmann	rs7665304	4	109025379	SBP	A	0.22	1.20E-08	-0.33	-0.11	-0.24	2.41E-01	7.33E-01	2.66E-01	1.00E+00	1.00	1.00E+00	-0.24	2.66E-01
Hoffmann	rs7665304	4	109025379	DBP	A	0.13	6.10E-08	-0.14	0.11	-0.04	4.27E-01	6.12E-01	7.87E-01	1.00E+00	1.00	1.00E+00	-0.04	7.87E-01
Hoffmann	rs66887589	4	120509279	SBP	T	-0.21	1.80E-08	-0.24	-0.72	-0.43	3.96E-01	3.34E-02	4.51E-02	9.83E-01	0.56	3.83E-01	-0.43	4.51E-02
Hoffmann	rs66887589	4	120509279	DBP	T	-0.21	9.20E-19	-0.18	-0.42	-0.28	3.06E-01	4.48E-02	3.88E-02	9.30E-01	0.61	3.09E-01	-0.28	3.88E-02
Hoffmann	rs893929	4	144187380	SBP	G	0.23	1.50E-09	-0.49	0.34	-0.15	8.02E-02	3.08E-01	4.85E-01	1.00E+00	0.93	1.00E+00	-0.15	4.85E-01
Hoffmann	rs893929	4	144187380	DBP	G	0.12	1.20E-07	-0.13	0.25	0.03	4.53E-01	2.22E-01	8.38E-01	1.00E+00	0.86	1.00E+00	0.03	8.38E-01
Hoffmann	rs4292285	4	145271954	SBP	T	0.23	3.00E-09	-0.02	0.59	0.22	9.47E-01	8.43E-02	3.12E-01	1.00E+00	0.66	7.16E-01	0.22	3.12E-01
Hoffmann	rs4292285	4	145271954	DBP	T	0.15	6.90E-11	0.13	0.21	0.16	4.40E-01	3.35E-01	2.32E-01	1.00E+00	0.92	6.23E-01	0.16	2.32E-01
Hoffmann	rs7666150	4	146814640	SBP	T	0.21	9.50E-08	0.69	0.91	0.79	2.44E-02	6.31E-03	4.91E-04	3.46E-01	0.43	5.74E-02	0.79	4.91E-04
Hoffmann	rs7666150	4	146814640	PP	T	0.15	5.60E-09	0.29	0.31	0.30	1.54E-01	1.56E-01	4.54E-02	7.07E-01	0.75	3.10E-01	0.30	4.54E-02
HMG	rs13143871	4	156619204	DBP	T	0.49	5.52E-07	0.41	0.80	0.57	3.74E-02	6.64E-04	1.62E-04	1.18E-01	0.03	8.01E-03	0.51	6.93E-10

Table S8: Generalization of previously reported loci associated with quantitative BP traits.

Ref	rsID	chr	position	trait	A1	discovery		HCHS/SOL beta			HCHS/SOL p-value			FDR$_g$ r-value			all meta	
						beta	p-value	Main	Carib	All	Main	Carib	All	Main	Carib	All	beta	p-value
HMG	rs13143871	4	156619204	SBP	T	0.96	5.16E-08	0.69	1.04	0.83	3.00E-02	6.06E-03	6.33E-04	1.43E-01	0.06	4.01E-03	0.91	2.62E-10
Ehret '11	rs13139571	4	156645513	DBP	C	0.26	2.20E-10	0.42	0.81	0.58	3.65E-02	8.11E-04	1.87E-04	1.18E-01	0.01	1.36E-03	0.28	1.34E-12
Ehret '11	rs13139571	4	156645513	SBP	C	0.32	1.20E-06	0.75	0.90	0.81	1.95E-02	2.17E-02	1.13E-03	1.25E-01	0.16	7.69E-02	0.35	3.23E-08
Hoffmann	rs184145372	4	159150358	PP	T	-5.78	1.80E-08	-7.70	-4.42	-5.08	5.58E-02	2.81E-02	4.83E-03	5.44E-01	0.44	1.91E-01	-5.08	4.83E-03
Hoffmann	rs869396	4	169688000	PP	C	0.22	5.90E-18	0.38	0.41	0.39	6.05E-02	5.32E-02	7.73E-03	5.44E-01	0.46	2.28E-01	0.39	7.73E-03
Hoffmann	rs185695143	5	10860486	SBP	C	-20.23	2.50E-08	5.60	-9.17	-3.53	8.46E-01	6.89E-01	8.44E-01	1.00E+00	1.00	1.00E+00	-3.53	8.44E-01
Hoffmann	rs185695143	5	10860486	PP	C	-13.54	1.20E-07	-9.09	-1.15	-4.03	6.35E-01	9.35E-01	7.24E-01	1.00E+00	1.00	1.00E+00	-4.03	7.24E-01
Hoffmann	rs114053299	5	12780703	PP	A	-6.49	4.80E-08	1.69	-0.14	0.29	7.36E-01	9.60E-01	9.04E-01	1.00E+00	1.00	1.00E+00	0.29	9.04E-01
Hoffmann	rs303343	5	15312553	SBP	C	-0.23	3.50E-09	-0.55	0.47	-0.05	9.94E-02	1.67E-01	8.36E-01	5.97E-01	1.00	1.00E+00	-0.05	8.36E-01
Hoffmann	rs303343	5	15312553	PP	C	-0.12	2.40E-06	-0.18	0.11	-0.03	4.23E-01	6.03E-01	8.46E-01	1.00E+00	1.00	1.00E+00	-0.03	8.46E-01
Ehret '11	rs1173771	5	32815028	DBP	G	0.26	9.10E-12	0.50	0.01	0.31	3.90E-03	9.81E-01	2.31E-02	2.83E-02	1.00	5.57E-02	0.26	7.00E-13
Ehret '11	rs1173771	5	32815028	SBP	G	0.50	1.80E-16	0.94	0.09	0.62	7.62E-04	7.97E-01	5.24E-03	7.37E-03	1.00	2.21E-02	0.51	3.91E-18
Hoffmann	rs168643	5	50935900	SBP	T	0.20	9.20E-07	-0.27	-0.50	-0.36	3.39E-01	1.60E-01	1.07E-01	1.00E+00	1.00	1.00E+00	-0.36	1.07E-01
Hoffmann	rs168643	5	50935900	DBP	T	0.14	1.50E-08	-0.02	-0.07	-0.04	9.11E-01	7.42E-01	7.69E-01	1.00E+00	1.00	1.00E+00	-0.04	7.69E-01
Hoffmann	rs1694068	5	53283630	SBP	T	-0.24	7.00E-10	0.31	0.18	0.25	3.07E-01	6.12E-01	2.72E-01	1.00E+00	1.00	1.00E+00	0.25	2.72E-01
Hoffmann	rs1694068	5	53283630	PP	T	-0.16	6.40E-10	0.19	0.07	0.14	3.45E-01	7.64E-01	3.64E-01	1.00E+00	1.00	1.00E+00	0.14	3.64E-01
Hoffmann	rs111304266	5	56589542	DBP	C	-0.37	3.30E-08	-0.34	0.42	0.06	6.00E-01	4.97E-01	8.90E-01	1.00E+00	1.00	1.00E+00	0.06	8.90E-01
Hoffmann	rs7714219	5	71654855	PP	G	-0.16	3.80E-09	-0.00	0.21	0.08	9.92E-01	3.68E-01	5.81E-01	1.00E+00	1.00	1.00E+00	0.08	5.81E-01
Hoffmann	rs258494	5	75038718	DBP	C	0.22	4.50E-19	0.05	0.06	0.05	7.79E-01	7.91E-01	7.01E-01	1.00E+00	1.00	1.00E+00	0.05	7.01E-01
Hoffmann	rs10057188	5	77837789	SBP	G	0.18	2.30E-06	0.87	-0.33	0.30	1.20E-02	3.70E-01	2.33E-01	3.46E-01	1.00	6.58E-01	0.30	2.33E-01
Hoffmann	rs10057188	5	77837789	PP	G	0.15	3.40E-09	0.39	-0.06	0.17	8.76E-02	7.84E-01	3.06E-01	5.60E-01	1.00	7.96E-01	0.17	3.06E-01
Hoffmann	rs17286052	5	87430302	SBP	A	0.36	6.50E-11	0.08	0.20	0.14	8.65E-01	6.85E-01	6.90E-01	1.00E+00	1.00	1.00E+00	0.14	6.90E-01
Hoffmann	rs17286052	5	87430302	DBP	A	0.19	2.60E-08	-0.04	0.28	0.12	9.06E-01	3.80E-01	5.99E-01	1.00E+00	0.95	1.00E+00	0.12	5.99E-01

Table S8: Generalization of previously reported loci associated with quantitative BP traits.

Ref	rsID	chr	position	trait	A1	discovery		HCHS/SOL beta			HCHS/SOL p-value			FDR$_g$ r-value			all meta	
						beta	p-value	Main	Carib	All	Main	Carib	All	Main	Carib	All	beta	p-value
Hoffmann	rs17286052	5	87430302	PP	A	0.17	5.50E-06	0.10	-0.09	0.01	7.50E-01	7.87E-01	9.65E-01	1.00E+00	1.00	1.00E+00	0.01	9.65E-01
Hoffmann	rs17082391	5	91900785	SBP	C	0.54	4.60E-07	-1.16	1.47	0.42	2.19E-01	5.61E-02	4.83E-01	1.00E+00	0.56	8.65E-01	0.42	4.83E-01
Hoffmann	rs17082391	5	91900785	DBP	C	0.38	1.30E-08	-0.64	0.53	0.06	2.81E-01	2.70E-01	8.62E-01	1.00E+00	0.88	1.00E+00	0.06	8.62E-01
Hoffmann	rs62361303	5	108102727	SBP	C	0.26	6.40E-07	0.90	0.09	0.46	6.43E-02	8.37E-01	1.61E-01	5.44E-01	1.00	6.36E-01	0.46	1.61E-01
Hoffmann	rs62361303	5	108102727	PP	C	0.23	1.90E-11	0.53	0.33	0.42	9.68E-02	2.55E-01	5.03E-02	5.60E-01	0.83	3.22E-01	0.42	5.03E-02
Hoffmann	rs4475250	5	114375552	SBP	G	0.24	2.20E-10	0.25	-0.13	0.09	3.71E-01	6.95E-01	6.63E-01	9.55E-01	1.00	1.00E+00	0.09	6.63E-01
Hoffmann	rs4475250	5	114375552	DBP	G	0.14	7.10E-09	0.12	0.15	0.13	4.86E-01	4.71E-01	3.22E-01	1.00E+00	1.00	7.79E-01	0.13	3.22E-01
Ehret '16	rs10077885	5	114390121	SBP	A	-0.28	1.64E-10	-0.30	-0.16	-0.24	2.98E-01	6.47E-01	2.77E-01	5.53E-01	1.00	5.86E-01	-0.28	8.86E-11
Ehret '16	rs10077885	5	114390121	DBP	A	-0.17	3.99E-11	-0.09	-0.34	-0.20	6.01E-01	1.09E-01	1.55E-01	1.00E+00	0.45	4.71E-01	-0.17	1.51E-11
Hoffmann	rs2914609	5	121287061	SBP	T	0.23	1.80E-06	0.31	-0.12	0.10	3.99E-01	7.43E-01	7.13E-01	9.83E-01	1.00	1.00E+00	0.10	7.13E-01
Hoffmann	rs2914609	5	121287061	PP	T	0.19	9.40E-09	0.36	0.05	0.21	1.31E-01	8.27E-01	2.24E-01	6.50E-01	1.00	6.35E-01	0.21	2.24E-01
Kato	rs13359291	5	122476457	SBP	A	0.53	8.90E-16	0.82	0.19	0.62	9.50E-03	6.80E-01	1.78E-02	2.85E-02	0.68	5.33E-02	0.54	2.26E-15
Liu	rs4530754	5	122855416	PP	G	0.17	9.90E-08	-0.06	-0.36	-0.18	7.31E-01	1.24E-01	2.22E-01	1.00E+00	1.00	1.00E+00	0.16	1.14E-07
Hoffmann	rs6595838	5	127868199	SBP	G	-0.27	1.80E-10	-0.63	-0.57	-0.60	4.92E-02	1.14E-01	1.23E-02	5.23E-01	0.66	2.51E-01	-0.60	1.23E-02
Hoffmann	rs6595838	5	127868199	DBP	G	-0.13	1.90E-07	-0.17	-0.54	-0.34	4.00E-01	1.51E-02	2.52E-02	1.00E+00	0.58	2.88E-01	-0.34	2.52E-02
Hoffmann	rs6595838	5	127868199	PP	G	-0.14	1.00E-06	-0.43	-0.00	-0.24	3.96E-02	9.87E-01	1.29E-01	5.44E-01	1.00	4.96E-01	-0.24	1.29E-01
Liu	rs2188962	5	131770805	DBP	T	-0.20	3.00E-11	-0.07	-0.23	-0.14	7.47E-01	3.24E-01	3.76E-01	1.00E+00	0.76	7.52E-01	-0.20	1.87E-11
Hoffmann	rs7734334	5	131815004	DBP	C	-0.15	8.70E-10	-0.34	-0.28	-0.31	6.33E-02	1.97E-01	2.53E-02	5.49E-01	0.85	2.88E-01	-0.31	2.53E-02
Hoffmann	rs114534	5	142533657	PP	G	0.15	2.40E-09	0.05	0.01	0.03	8.03E-01	9.78E-01	8.34E-01	1.00E+00	1.00	1.00E+00	0.03	8.34E-01
Kato	rs9687065	5	148391140	DBP	A	0.26	7.40E-11	0.05	0.29	0.15	8.19E-01	3.19E-01	4.24E-01	1.00E+00	0.64	8.47E-01	0.25	6.95E-11
Ehret '11	rs11953630	5	157845402	DBP	T	-0.28	3.80E-13	0.10	-0.15	-0.02	6.46E-01	5.04E-01	8.89E-01	1.00E+00	0.77	9.55E-01	-0.27	1.32E-12
Ehret '11	rs11953630	5	157845402	SBP	T	-0.41	3.00E-11	-0.47	-0.24	-0.36	1.86E-01	5.09E-01	1.60E-01	3.82E-01	0.98	3.32E-01	-0.41	1.12E-11
Hoffmann	rs148871069	5	159404471	SBP	A	2.37	3.90E-11	-3.82	-0.79	-2.03	4.93E-01	8.65E-01	5.69E-01	1.00E+00	1.00	1.00E+00	-2.03	5.69E-01

Table S8: Generalization of previously reported loci associated with quantitative BP traits.

Ref	rsID	chr	position	trait	A1	discovery		HCHS/SOL beta			HCHS/SOL p-value			FDR$_g$ r-value			all meta	
						beta	p-value	Main	Carib	All	Main	Carib	All	Main	Carib	All	beta	p-value
Hoffmann	rs148871069	5	159404471	DBP	A	1.12	7.50E-08	-1.65	-0.12	-0.75	6.31E-01	9.68E-01	7.35E-01	1.00E+00	1.00	1.00E+00	-0.75	7.35E-01
Hoffmann	rs148871069	5	159404471	PP	A	1.26	4.20E-07	-2.87	-0.49	-1.45	4.30E-01	8.70E-01	5.30E-01	1.00E+00	1.00	1.00E+00	-1.45	5.30E-01
Hoffmann	rs72812846	5	173377636	SBP	T	0.20	9.10E-06	0.02	-0.68	-0.29	9.54E-01	1.28E-01	3.32E-01	1.00E+00	1.00	1.00E+00	-0.29	3.32E-01
Hoffmann	rs72812846	5	173377636	DBP	T	0.20	1.00E-13	0.16	-0.09	0.05	5.23E-01	7.46E-01	7.98E-01	1.00E+00	1.00	1.00E+00	0.05	7.98E-01
Hoffmann	rs1923409	6	7728212	PP	G	-0.16	1.50E-10	0.19	0.53	0.34	3.27E-01	1.69E-02	2.12E-02	1.00E+00	1.00	1.00E+00	0.34	2.12E-02
Hoffmann	rs9349379	6	12903957	SBP	A	0.19	3.80E-06	0.29	0.47	0.36	3.22E-01	2.05E-01	1.20E-01	9.42E-01	0.87	6.36E-01	0.36	1.20E-01
Hoffmann	rs9349379	6	12903957	PP	A	0.20	6.30E-13	0.32	0.07	0.23	8.68E-02	7.78E-01	1.30E-01	5.60E-01	1.00	4.96E-01	0.23	1.30E-01
Hoffmann	rs4712656	6	22136262	SBP	G	-0.23	2.90E-09	-0.29	-0.38	-0.33	3.33E-01	2.74E-01	1.51E-01	9.43E-01	0.89	6.36E-01	-0.33	1.51E-01
Hoffmann	rs4712656	6	22136262	DBP	G	-0.12	9.60E-08	-0.09	-0.13	-0.11	6.14E-01	5.39E-01	4.37E-01	1.00E+00	1.00	8.69E-01	-0.11	4.37E-01
Ehret '11	rs1799945	6	26091179	DBP	G	0.46	1.50E-15	-0.22	0.42	0.06	4.27E-01	1.80E-01	7.79E-01	1.00E+00	0.43	8.69E-01	0.43	8.25E-15
Ehret '11	rs1799945	6	26091179	SBP	G	0.63	7.70E-12	0.06	0.74	0.35	8.85E-01	1.49E-01	2.93E-01	9.77E-01	0.48	4.69E-01	0.61	6.09E-12
HMG	rs1799945	6	26091179	DBP	G	0.88	2.20E-05	-0.22	0.42	0.06	4.27E-01	1.80E-01	7.79E-01	1.00E+00	0.42	9.86E-01	0.48	7.75E-04
HMG	rs1799945	6	26091179	SBP	G	0.95	4.51E-03	0.06	0.74	0.35	8.85E-01	1.49E-01	2.93E-01	1.00E+00	1.00	1.00E+00	0.63	1.04E-02
Hoffmann	rs169287	6	27854760	DBP	C	0.21	5.10E-12	0.15	-0.21	0.00	5.59E-01	4.95E-01	9.93E-01	1.00E+00	1.00	1.00E+00	0.00	9.93E-01
Hoffmann	rs169287	6	27854760	PP	C	-0.19	3.20E-08	-0.42	0.46	-0.05	1.13E-01	1.42E-01	7.92E-01	5.87E-01	1.00	1.00E+00	-0.05	7.92E-01
Liu	rs926552	6	29548089	DBP	T	-0.26	7.20E-08	0.21	0.31	0.26	5.91E-01	4.25E-01	3.45E-01	1.00E+00	1.00	1.00E+00	-0.24	7.59E-07
HMG	rs9266359	6	31332739	DBP	C	0.29	1.76E-07	0.02	0.29	0.13	9.33E-01	2.27E-01	4.03E-01	1.00E+00	0.42	5.89E-01	0.27	1.51E-06
HMG	rs9266359	6	31332739	SBP	C	0.44	7.07E-06	0.20	0.36	0.27	5.27E-01	3.56E-01	2.82E-01	7.70E-01	0.61	4.77E-01	0.42	7.33E-06
Ehret '11	rs805303	6	31616366	DBP	G	0.23	3.00E-11	0.07	0.29	0.16	6.79E-01	1.60E-01	2.23E-01	7.88E-01	0.42	3.40E-01	0.22	1.57E-11
Ehret '11	rs805303	6	31616366	SBP	G	0.38	1.50E-11	0.28	0.34	0.31	3.11E-01	3.14E-01	1.54E-01	5.07E-01	0.66	3.32E-01	0.37	5.60E-12
Liu	rs409558	6	31708147	PP	G	-0.26	2.70E-09	-0.39	-0.29	-0.35	4.82E-02	2.57E-01	2.41E-02	1.47E-01	0.34	6.42E-02	-0.27	7.06E-12
Hoffmann	rs147384090	6	32013850	PP	C	-0.29	7.70E-17	2.80	2.45	2.58	5.11E-01	4.59E-01	3.24E-01	1.00E+00	1.00	1.00E+00	-0.29	8.66E-17
HMG	rs2021783	6	32044851	DBP	C	0.49	2.18E-12	0.46	0.36	0.44	2.61E-01	6.64E-01	2.31E-01	5.51E-01	0.88	4.38E-01	0.49	1.24E-12

Table S8: Generalization of previously reported loci associated with quantitative BP traits.

Ref	rsID	chr	position	trait	A1	discovery		HCHS/SOL beta			HCHS/SOL p-value			FDR$_g$ r-value			all meta	
						beta	p-value	Main	Carib	All	Main	Carib	All	Main	Carib	All	beta	p-value
HMG	rs2021783	6	32044851	SBP	C	0.68	3.19E-09	0.47	1.58	0.68	4.78E-01	2.38E-01	2.49E-01	7.57E-01	0.49	4.73E-01	0.68	7.35E-09
Hoffmann	rs3129927	6	32333827	PP	C	0.29	2.60E-09	1.31	-0.15	0.60	3.33E-02	8.11E-01	1.75E-01	5.44E-01	1.00	5.77E-01	0.29	1.29E-09
Hoffmann	rs210156	6	33517362	DBP	A	-0.15	2.00E-09	0.08	-0.05	0.03	6.53E-01	8.05E-01	8.55E-01	1.00E+00	1.00	1.00E+00	0.03	8.55E-01
Hoffmann	rs1544935	6	39124448	DBP	T	0.18	8.90E-10	0.47	0.33	0.41	3.44E-02	2.10E-01	1.56E-02	4.78E-01	0.85	2.18E-01	0.41	1.56E-02
Hoffmann	rs649472	6	42673015	PP	T	0.16	1.20E-08	-0.39	0.39	-0.03	1.10E-01	1.42E-01	8.50E-01	1.00E+00	0.74	1.00E+00	-0.03	8.50E-01
Liu	rs2270860	6	43270151	SBP	T	0.32	2.90E-11	0.50	0.42	0.47	7.35E-02	2.33E-01	3.31E-02	2.94E-01	0.72	2.65E-01	0.33	1.91E-11
Kato	rs1563788	6	43308363	SBP	T	0.51	2.20E-16	0.41	0.36	0.39	1.48E-01	3.07E-01	7.91E-02	2.23E-01	0.65	1.19E-01	0.50	4.59E-18
Hoffmann	rs13205180	6	51832494	DBP	C	-0.14	1.40E-09	-0.01	-0.42	-0.18	9.39E-01	5.18E-02	1.98E-01	1.00E+00	0.61	6.02E-01	-0.18	1.98E-01
Hoffmann	rs670463	6	53977495	SBP	A	-0.19	2.30E-06	-0.36	-0.27	-0.33	2.01E-01	4.17E-01	1.34E-01	8.13E-01	1.00	6.36E-01	-0.33	1.34E-01
Hoffmann	rs670463	6	53977495	PP	A	-0.16	2.10E-09	-0.32	-0.24	-0.28	8.26E-02	2.78E-01	4.33E-02	5.60E-01	0.86	3.10E-01	-0.28	4.33E-02
Hoffmann	rs4140574	6	56099424	PP	T	0.23	4.30E-19	0.39	0.13	0.28	3.15E-02	5.66E-01	4.50E-02	5.44E-01	1.00	3.10E-01	0.28	4.50E-02
Liu	rs1925153d	6	56102780	PP	T	-0.19	4.90E-08	-0.40	-0.17	-0.29	4.82E-02	4.36E-01	5.11E-02	1.47E-01	0.50	1.02E-01	-0.20	3.55E-07
Liu	rs10943605	6	79655477	DBP	A	0.16	3.30E-09	0.03	-0.11	-0.03	8.66E-01	5.96E-01	8.36E-01	1.00E+00	1.00	1.00E+00	0.15	2.45E-07
Hoffmann	rs2050663	6	79753394	SBP	T	-0.23	3.60E-09	-0.19	-0.08	-0.14	4.98E-01	8.23E-01	5.10E-01	1.00E+00	1.00	8.67E-01	-0.14	5.10E-01
Hoffmann	rs2050663	6	79753394	DBP	T	-0.17	1.60E-13	-0.03	0.10	0.03	8.74E-01	6.26E-01	8.51E-01	1.00E+00	1.00	1.00E+00	0.03	8.51E-01
Franceschini	rs6924906	6	82220552	SBP	T	0.41	6.20E-05	0.58	0.14	0.32	3.90E-01	8.10E-01	4.65E-01	1.00E+00	1.00	1.00E+00	0.41	4.77E-05
Franceschini	rs6924906	6	82220552	DBP	T	0.51	5.50E-07	0.32	-0.00	0.13	4.54E-01	9.94E-01	6.38E-01	6.35E-01	1.00	8.94E-01	0.46	1.18E-06
Hoffmann	rs35410524	6	96885405	SBP	C	-0.30	4.70E-10	-0.71	0.13	-0.35	5.70E-02	7.67E-01	2.13E-01	5.44E-01	1.00	6.58E-01	-0.35	2.13E-01
Hoffmann	rs35410524	6	96885405	PP	C	-0.17	3.70E-07	-0.36	-0.13	-0.26	1.45E-01	6.40E-01	1.61E-01	6.83E-01	1.00	5.58E-01	-0.26	1.61E-01
Hoffmann	rs36061333	6	116311763	DBP	C	0.16	2.10E-08	0.15	-0.01	0.07	5.58E-01	9.75E-01	6.95E-01	1.00E+00	1.00	1.00E+00	0.07	6.95E-01
Hoffmann	rs1761870	6	117264985	DBP	G	0.17	3.50E-09	0.13	-0.03	0.07	5.24E-01	8.93E-01	6.82E-01	1.00E+00	1.00	1.00E+00	0.07	6.82E-01
Hoffmann	rs35189230	6	117816351	DBP	G	0.24	1.00E-08	0.45	0.12	0.30	4.96E-02	6.24E-01	7.66E-02	5.12E-01	1.00	4.02E-01	0.30	7.66E-02
Hoffmann	rs1630266	6	118612943	SBP	G	-0.33	1.50E-06	-0.03	0.31	0.10	9.44E-01	5.94E-01	7.80E-01	1.00E+00	1.00	1.00E+00	0.10	7.80E-01

Table S8: Generalization of previously reported loci associated with quantitative BP traits.

Ref	rsID	chr	position	trait	A1	discovery		HCHS/SOL beta			HCHS/SOL p-value			FDR_g r-value			all meta	
						beta	p-value	Main	Carib	All	Main	Carib	All	Main	Carib	All	beta	p-value
Hoffmann	rs1630266	6	118612943	DBP	G	-0.25	2.80E-09	-0.11	-0.14	-0.12	7.03E-01	6.97E-01	5.90E-01	1.00E+00	1.00	1.00E+00	-0.12	5.90E-01
Hoffmann	rs12206253	6	122192592	PP	C	0.29	1.10E-12	0.18	0.46	0.33	5.78E-01	1.07E-01	1.20E-01	1.00E+00	0.66	4.96E-01	0.33	1.20E-01
Franceschini	rs13209747	6	127115454	SBP	T	0.85	2.60E-10	0.12	0.40	0.24	6.99E-01	2.58E-01	3.09E-01	1.00E+00	1.00	8.10E-01	0.69	2.30E-09
Franceschini	rs13209747	6	127115454	DBP	T	0.56	2.40E-11	0.19	0.27	0.23	3.14E-01	2.12E-01	1.17E-01	6.35E-01	0.74	4.10E-01	0.48	5.38E-11
Hoffmann	rs1570350	6	143592386	PP	A	0.20	7.40E-15	0.10	-0.06	0.03	5.85E-01	7.77E-01	8.12E-01	1.00E+00	1.00	1.00E+00	0.03	8.12E-01
Franceschini	rs17080102	6	151004770	SBP	C	1.02	4.80E-08	-0.83	-0.82	-0.83	2.38E-02	1.38E-01	7.25E-03	1.00E+00	1.00	1.00E+00	0.52	1.06E-03
Franceschini	rs17080102	6	151004770	DBP	C	0.74	1.90E-11	-0.29	-0.51	-0.36	2.01E-01	1.37E-01	6.05E-02	1.00E+00	1.00	1.00E+00	0.47	1.01E-06
Hoffmann	rs13192976	6	152312415	SBP	A	-0.29	7.50E-07	-0.35	-0.10	-0.20	5.51E-01	8.29E-01	5.84E-01	1.00E+00	1.00	9.44E-01	-0.20	5.84E-01
Hoffmann	rs13192976	6	152312415	PP	A	-0.43	8.20E-28	-0.45	-0.29	-0.35	2.46E-01	3.53E-01	1.48E-01	8.25E-01	0.94	5.31E-01	-0.35	1.48E-01
Hoffmann	rs12208834	6	157287299	PP	A	-0.15	4.20E-08	0.04	-0.12	-0.03	8.60E-01	6.15E-01	8.49E-01	1.00E+00	1.00	1.00E+00	-0.03	8.49E-01
Hoffmann	rs516143	6	159696185	PP	G	0.31	1.40E-16	-1.61	-0.60	-1.09	1.73E-01	5.98E-01	1.85E-01	1.00E+00	1.00	1.00E+00	-1.09	1.85E-01
Hoffmann	rs4709746	6	164133001	DBP	C	0.19	4.10E-08	0.30	0.42	0.33	1.10E-01	1.76E-01	4.03E-02	5.49E-01	0.83	3.09E-01	0.33	4.03E-02
Hoffmann	rs903432	6	166175471	SBP	A	0.39	1.70E-07	0.71	1.32	1.07	2.24E-01	7.59E-03	4.86E-03	8.26E-01	0.43	2.51E-01	1.07	4.86E-03
Hoffmann	rs903432	6	166175471	DBP	A	0.31	5.10E-12	0.53	0.87	0.73	1.46E-01	4.51E-03	1.94E-03	6.21E-01	0.58	8.51E-02	0.73	1.94E-03
Hoffmann	rs1322639	6	169587103	PP	G	-0.30	1.20E-23	-0.33	-0.24	-0.29	7.89E-02	3.18E-01	4.66E-02	5.60E-01	0.92	3.10E-01	-0.29	4.66E-02
Hoffmann	rs12670854	7	1731866	SBP	A	0.36	1.20E-08	-0.71	-0.79	-0.75	1.69E-01	1.15E-01	3.78E-02	1.00E+00	1.00	1.00E+00	-0.75	3.78E-02
Hoffmann	rs12670854	7	1731866	DBP	A	0.17	8.70E-06	-0.15	-0.52	-0.34	6.35E-01	9.70E-02	1.30E-01	1.00E+00	1.00	1.00E+00	-0.34	1.30E-01
Hoffmann	rs11486794	7	2491918	SBP	C	0.30	2.80E-07	-0.26	0.05	-0.10	6.09E-01	9.23E-01	7.79E-01	1.00E+00	1.00	1.00E+00	-0.10	7.79E-01
Hoffmann	rs11486794	7	2491918	DBP	C	0.22	7.70E-10	0.13	0.03	0.08	6.82E-01	9.27E-01	7.28E-01	1.00E+00	1.00	1.00E+00	0.08	7.28E-01
Ehret '16	rs2969070	7	2512545	SBP	A	-0.30	1.44E-10	-0.16	0.61	0.13	5.79E-01	1.03E-01	5.81E-01	8.36E-01	1.00	1.00E+00	-0.28	6.57E-10
Ehret '16	rs2969070	7	2512545	DBP	A	-0.18	2.92E-11	0.21	0.22	0.21	2.57E-01	3.55E-01	1.45E-01	1.00E+00	1.00	1.00E+00	-0.17	3.98E-10
Kato	rs2107595	7	19049388	PP	A	0.31	3.90E-11	0.25	0.07	0.19	2.33E-01	7.96E-01	2.69E-01	4.67E-01	1.00	5.38E-01	0.30	3.92E-10
Liu	rs6969780	7	27159136	DBP	C	0.26	1.10E-08	0.41	-0.17	0.21	4.15E-02	5.30E-01	2.05E-01	2.90E-01	1.00	6.79E-01	0.26	9.11E-08

Table S8: Generalization of previously reported loci associated with quantitative BP traits.

Ref	rsID	chr	position	trait	A1	discovery		HCHS/SOL beta			HCHS/SOL p-value			FDR$_g$ r-value			all meta	
						beta	p-value	Main	Carib	All	Main	Carib	All	Main	Carib	All	beta	p-value
Franceschini	rs17428471	7	27337867	SBP	T	1.20	2.10E-12	0.37	0.42	0.40	5.59E-01	4.52E-01	3.44E-01	1.00E+00	1.00	8.10E-01	1.09	6.64E-12
Franceschini	rs17428471	7	27337867	DBP	T	0.61	1.60E-09	0.31	0.30	0.31	4.31E-01	3.84E-01	2.42E-01	6.35E-01	0.90	5.65E-01	0.57	1.45E-09
Hoffmann	rs917275	7	28658522	PP	A	-0.18	7.50E-12	-0.06	0.31	0.12	8.16E-01	2.17E-01	4.92E-01	1.00E+00	1.00	1.00E+00	0.12	4.92E-01
Hoffmann	rs12538229	7	40460129	SBP	C	0.30	1.50E-06	0.69	-0.01	0.44	1.14E-01	9.83E-01	2.12E-01	6.45E-01	1.00	6.58E-01	0.44	2.12E-01
Hoffmann	rs12538229	7	40460129	PP	C	0.29	2.40E-12	0.59	0.01	0.38	3.98E-02	9.71E-01	9.77E-02	5.44E-01	1.00	4.65E-01	0.38	9.77E-02
Hoffmann	rs2971669	7	44231778	PP	C	-0.17	3.40E-08	-0.22	0.05	-0.10	2.94E-01	8.26E-01	5.27E-01	9.55E-01	1.00	9.48E-01	-0.10	5.27E-01
Liu	rs11977526	7	46008110	PP	A	-0.36	2.90E-29	-0.40	-0.40	-0.40	5.51E-02	7.70E-02	9.70E-03	1.47E-01	0.21	3.88E-02	-0.36	1.27E-34
Kato	rs10260816	7	46010100	PP	C	0.32	1.50E-14	0.45	0.39	0.42	3.02E-02	7.60E-02	5.55E-03	1.21E-01	0.30	2.22E-02	0.33	3.10E-17
Hoffmann	rs34594435	7	72977249	DBP	C	0.18	1.30E-09	-0.26	0.18	-0.07	3.72E-01	5.79E-01	7.64E-01	1.00E+00	1.00	1.00E+00	-0.07	7.64E-01
Hoffmann	rs76627715	7	80387316	DBP	T	0.22	1.10E-09	-0.12	0.08	-0.05	5.83E-01	7.93E-01	7.66E-01	1.00E+00	1.00	1.00E+00	-0.05	7.66E-01
Hoffmann	rs560276033	7	91268940	SBP	GTA	-0.47	3.50E-06	-0.13	0.62	0.18	7.26E-01	1.60E-01	5.23E-01	1.00E+00	1.00	1.00E+00	0.18	5.23E-01
Hoffmann	rs560276033	7	91268940	PP	GTA	-0.43	1.60E-09	-0.02	0.06	0.01	9.31E-01	8.30E-01	9.46E-01	1.00E+00	1.00	1.00E+00	0.01	9.46E-01
Hoffmann	rs1015538	7	99626035	DBP	A	0.14	3.40E-08	0.13	0.38	0.24	5.10E-01	1.03E-01	1.18E-01	1.00E+00	0.72	4.89E-01	0.24	1.18E-01
Hoffmann	rs34489224	7	100525559	PP	C	0.18	3.80E-10	-0.38	-0.41	-0.39	6.93E-02	1.05E-01	1.53E-02	1.00E+00	1.00	1.00E+00	-0.39	1.53E-02
Hoffmann	rs17423264	7	108090255	SBP	C	0.37	4.90E-08	0.88	-0.01	0.40	2.20E-01	9.89E-01	4.15E-01	8.26E-01	1.00	8.11E-01	0.40	4.15E-01
Hoffmann	rs17423264	7	108090255	DBP	C	0.18	5.10E-06	0.25	0.10	0.17	5.71E-01	8.06E-01	5.75E-01	1.00E+00	1.00	1.00E+00	0.17	5.75E-01
Hoffmann	rs1966323	7	116571847	PP	T	0.17	1.60E-09	-0.26	-0.04	-0.16	1.96E-01	8.66E-01	2.82E-01	1.00E+00	1.00	1.00E+00	-0.16	2.82E-01
Ehret ’16	rs11556924a	7	129663496	SBP	T	-0.27	7.64E-09	0.25	0.26	0.25	5.02E-01	5.12E-01	3.53E-01	1.00E+00	1.00	1.00E+00	-0.26	3.08E-08
Ehret ’16	rs11556924a	7	129663496	DBP	T	-0.21	8.15E-15	0.04	-0.51	-0.23	8.79E-01	3.40E-02	1.81E-01	1.00E+00	0.34	4.71E-01	-0.21	3.51E-15
Hoffmann	rs11556924	7	129663496	SBP	C	0.19	3.80E-06	-0.25	-0.26	-0.25	5.02E-01	5.12E-01	3.53E-01	1.00E+00	1.00	1.00E+00	-0.25	3.53E-01
Hoffmann	rs11556924	7	129663496	DBP	C	0.18	7.50E-13	-0.04	0.51	0.23	8.79E-01	3.40E-02	1.81E-01	1.00E+00	0.61	6.02E-01	0.23	1.81E-01
Hoffmann	rs6957161	7	131361319	SBP	A	0.26	2.10E-09	0.30	0.00	0.18	2.95E-01	9.91E-01	4.13E-01	8.97E-01	1.00	8.11E-01	0.18	4.13E-01
Hoffmann	rs6957161	7	131361319	DBP	A	0.16	1.70E-09	0.29	0.22	0.26	1.08E-01	3.13E-01	6.17E-02	5.49E-01	0.90	3.63E-01	0.26	6.17E-02

Table S8: Generalization of previously reported loci associated with quantitative BP traits.

Ref	rsID	chr	position	trait	A1	discovery		HCHS/SOL beta			HCHS/SOL p-value			FDR$_g$ r-value			all meta	
						beta	p-value	Main	Carib	All	Main	Carib	All	Main	Carib	All	beta	p-value
Hoffmann	rs273957	7	137600690	PP	C	-0.15	2.80E-08	0.07	-0.08	0.00	7.62E-01	7.54E-01	9.86E-01	1.00E+00	1.00	1.00E+00	0.00	9.86E-01
Liu	rs891511	7	150704843	DBP	A	-0.26	2.00E-16	-0.40	-0.37	-0.39	3.48E-02	8.68E-02	7.01E-03	2.90E-01	0.51	9.81E-02	-0.27	1.63E-19
Hoffmann	rs111630016	7	158048396	DBP	C	0.32	3.20E-08	0.20	-0.41	-0.10	7.39E-01	5.08E-01	8.22E-01	1.00E+00	1.00	1.00E+00	-0.10	8.22E-01
Hoffmann	rs80073370	8	19833156	SBP	A	0.40	8.40E-09	-0.43	0.78	0.09	4.83E-01	2.72E-01	8.53E-01	1.00E+00	0.89	1.00E+00	0.09	8.53E-01
Hoffmann	rs80073370	8	19833156	PP	A	0.22	2.50E-06	-0.47	0.70	0.04	2.43E-01	1.29E-01	9.05E-01	1.00E+00	0.73	1.00E+00	0.04	9.05E-01
Hoffmann	rs2280861	8	23404785	DBP	A	-0.17	6.70E-11	-0.12	-0.48	-0.26	5.08E-01	2.94E-02	6.12E-02	1.00E+00	0.61	3.63E-01	-0.26	6.12E-02
Hoffmann	rs7008914	8	25880400	SBP	T	0.24	4.50E-08	-0.21	-0.47	-0.31	4.58E-01	2.06E-01	1.78E-01	1.00E+00	1.00	1.00E+00	-0.31	1.78E-01
Hoffmann	rs28594215	8	32395518	PP	G	-0.14	2.10E-08	-0.07	-0.46	-0.23	6.98E-01	3.35E-02	9.67E-02	1.00E+00	0.44	4.65E-01	-0.23	9.67E-02
Hoffmann	rs10958717	8	42351585	PP	G	-0.17	1.00E-10	-0.18	-0.02	-0.12	3.33E-01	9.39E-01	4.20E-01	1.00E+00	1.00	8.76E-01	-0.12	4.20E-01
Hoffmann	rs11993898	8	51936632	SBP	T	-0.33	3.50E-11	-0.04	-0.77	-0.32	9.06E-01	4.74E-02	1.85E-01	1.00E+00	0.56	6.58E-01	-0.32	1.85E-01
Hoffmann	rs11993898	8	51936632	DBP	T	-0.16	1.30E-07	-0.20	-0.47	-0.30	3.16E-01	5.29E-02	4.68E-02	9.30E-01	0.61	3.11E-01	-0.30	4.68E-02
Hoffmann	rs11993898	8	51936632	PP	T	-0.16	7.30E-07	0.13	-0.32	-0.05	5.31E-01	1.95E-01	7.40E-01	1.00E+00	0.83	1.00E+00	-0.05	7.40E-01
Hoffmann	rs1350100	8	76054904	PP	A	0.15	4.20E-09	-0.04	0.14	0.03	8.23E-01	5.62E-01	8.58E-01	1.00E+00	1.00	1.00E+00	0.03	8.58E-01
Hoffmann	rs1449544	8	76591880	SBP	A	0.17	6.60E-06	0.45	0.11	0.31	1.02E-01	7.41E-01	1.42E-01	5.97E-01	1.00	6.36E-01	0.31	1.42E-01
Hoffmann	rs1449544	8	76591880	PP	A	0.20	2.70E-15	0.18	0.05	0.13	3.24E-01	8.03E-01	3.60E-01	1.00E+00	1.00	8.20E-01	0.13	3.60E-01
Hoffmann	rs7838781	8	77588716	SBP	A	0.23	3.70E-06	0.21	0.90	0.53	5.39E-01	1.58E-02	3.82E-02	1.00E+00	0.47	3.67E-01	0.53	3.82E-02
Hoffmann	rs7838781	8	77588716	PP	A	0.18	1.90E-08	0.30	0.40	0.34	1.87E-01	9.35E-02	3.69E-02	7.51E-01	0.66	3.10E-01	0.34	3.69E-02
Hoffmann	rs10103353	8	82849452	SBP	C	0.18	3.30E-06	0.20	-0.25	0.03	4.78E-01	4.86E-01	8.94E-01	1.00E+00	1.00	1.00E+00	0.03	8.94E-01
Hoffmann	rs10103353	8	82849452	DBP	C	0.15	4.30E-10	-0.08	0.10	-0.01	6.46E-01	6.56E-01	9.35E-01	1.00E+00	1.00	1.00E+00	-0.01	9.35E-01
Hoffmann	rs112875651	8	126506694	SBP	G	0.23	2.30E-08	0.67	-0.09	0.35	3.50E-02	8.06E-01	1.52E-01	4.17E-01	1.00	6.36E-01	0.35	1.52E-01
Hoffmann	rs112875651	8	126506694	DBP	G	0.11	8.20E-06	0.47	-0.10	0.23	1.82E-02	6.72E-01	1.33E-01	3.47E-01	1.00	5.36E-01	0.23	1.33E-01
Hoffmann	rs4631439	8	141059650	SBP	C	0.22	8.70E-06	0.10	0.41	0.23	7.30E-01	2.33E-01	3.06E-01	1.00E+00	0.87	7.14E-01	0.23	3.06E-01
Hoffmann	rs4631439	8	141059650	PP	C	0.20	2.20E-10	0.26	0.32	0.29	1.61E-01	1.42E-01	4.47E-02	7.10E-01	0.74	3.10E-01	0.29	4.47E-02

Table S8: Generalization of previously reported loci associated with quantitative BP traits.

Ref	rsID	chr	position	trait	A1	discovery		HCHS/SOL beta			HCHS/SOL p-value			FDR$_g$ r-value			all meta	
						beta	p-value	Main	Carib	All	Main	Carib	All	Main	Carib	All	beta	p-value
Hoffmann	rs76735299	8	142396481	SBP	G	-0.43	3.60E-08	0.23	-0.18	-0.00	7.26E-01	7.53E-01	9.98E-01	1.00E+00	1.00	1.00E+00	-0.00	9.98E-01
Hoffmann	rs76735299	8	142396481	DBP	G	-0.25	1.30E-07	0.27	-0.05	0.09	5.12E-01	8.93E-01	7.38E-01	1.00E+00	1.00	1.00E+00	0.09	7.38E-01
Hoffmann	rs62524579	8	144060955	SBP	G	0.24	2.70E-10	0.41	0.19	0.32	1.35E-01	5.75E-01	1.33E-01	6.77E-01	1.00	6.36E-01	0.32	1.33E-01
Hoffmann	rs62524579	8	144060955	DBP	G	0.15	5.60E-10	0.15	0.30	0.21	3.84E-01	1.49E-01	1.13E-01	1.00E+00	0.83	4.79E-01	0.21	1.13E-01
Hoffmann	rs7041664	9	8010674	SBP	C	-0.25	7.10E-09	-0.35	-0.41	-0.37	2.58E-01	3.29E-01	1.38E-01	8.47E-01	0.94	6.36E-01	-0.37	1.38E-01
Hoffmann	rs7041664	9	8010674	PP	C	-0.17	5.60E-09	-0.17	-0.12	-0.15	4.08E-01	6.68E-01	3.58E-01	1.00E+00	1.00	8.20E-01	-0.15	3.58E-01
Hoffmann	rs1333047	9	22124504	PP	A	-0.16	2.00E-10	0.21	-0.54	-0.08	2.45E-01	1.85E-02	6.00E-01	1.00E+00	0.44	1.00E+00	-0.08	6.00E-01
Liu	rs76452347	9	35906471	DBP	T	-0.23	6.80E-10	0.11	0.24	0.17	6.81E-01	3.82E-01	3.67E-01	1.00E+00	1.00	1.00E+00	-0.21	5.05E-08
Hoffmann	rs7019055	9	38088244	SBP	A	0.18	3.30E-06	0.03	0.20	0.10	9.28E-01	5.55E-01	6.56E-01	1.00E+00	1.00	1.00E+00	0.10	6.56E-01
Hoffmann	rs7019055	9	38088244	PP	A	0.15	7.10E-09	-0.04	0.09	0.01	8.09E-01	6.69E-01	9.24E-01	1.00E+00	1.00	1.00E+00	0.01	9.24E-01
Liu	rs111245230	9	113169775	SBP	C	0.70	1.20E-07	-0.47	1.22	0.35	6.38E-01	2.40E-01	6.32E-01	1.00E+00	0.72	7.23E-01	0.69	7.28E-08
Hoffmann	rs28663144	9	113198891	SBP	A	-0.69	3.50E-11	0.69	-1.28	-0.45	4.42E-01	9.41E-02	4.36E-01	1.00E+00	0.66	8.20E-01	-0.45	4.36E-01
Hoffmann	rs28663144	9	113198891	DBP	A	-0.32	4.70E-07	0.62	-1.19	-0.43	2.72E-01	1.24E-02	2.31E-01	1.00E+00	0.58	6.23E-01	-0.43	2.31E-01
Hoffmann	rs28663144	9	113198891	PP	A	-0.37	2.00E-07	0.12	-0.08	-0.00	8.44E-01	8.67E-01	9.97E-01	1.00E+00	1.00	1.00E+00	-0.00	9.97E-01
Ehret '16	rs10760117	9	123586737	SBP	T	0.28	6.10E-10	0.46	0.14	0.32	1.38E-01	6.78E-01	1.69E-01	3.59E-01	1.00	5.86E-01	0.28	2.27E-10
Ehret '16	rs10760117	9	123586737	DBP	T	0.10	2.08E-04	0.05	0.22	0.13	7.90E-01	3.15E-01	3.84E-01	1.00E+00	1.00	1.00E+00	0.10	1.39E-04
Liu	rs1953126	9	123640500	PP	T	0.17	1.80E-07	0.02	0.53	0.22	9.28E-01	3.01E-02	1.54E-01	1.00E+00	0.12	2.47E-01	0.17	5.38E-09
Hoffmann	rs7856420	9	123839157	PP	G	0.16	5.50E-09	0.40	0.06	0.22	9.77E-02	7.92E-01	1.87E-01	5.60E-01	1.00	5.81E-01	0.22	1.87E-01
Hoffmann	rs10818775	9	125755571	SBP	C	0.33	3.70E-09	0.50	0.26	0.42	8.59E-02	5.32E-01	8.06E-02	5.63E-01	1.00	5.71E-01	0.42	8.06E-02
Hoffmann	rs10818775	9	125755571	PP	C	0.26	1.90E-12	0.36	0.27	0.33	6.01E-02	3.11E-01	3.59E-02	5.44E-01	0.91	3.10E-01	0.33	3.59E-02
Hoffmann	rs139703184	9	127937746	SBP	T	-0.28	4.80E-07	-0.53	-0.30	-0.42	2.76E-01	5.50E-01	2.34E-01	8.53E-01	1.00	6.58E-01	-0.42	2.34E-01
Hoffmann	rs139703184	9	127937746	PP	T	-0.27	1.10E-13	-0.49	-0.28	-0.39	1.21E-01	3.82E-01	8.76E-02	6.15E-01	0.99	4.56E-01	-0.39	8.76E-02
Hoffmann	rs507666	9	136149399	DBP	G	0.17	1.70E-08	-0.35	0.55	0.05	1.67E-01	5.29E-02	7.90E-01	1.00E+00	0.61	1.00E+00	0.05	7.90E-01

Table S8: Generalization of previously reported loci associated with quantitative BP traits.

Ref	rsID	chr	position	trait	A1	discovery		HCHS/SOL beta			HCHS/SOL p-value			FDR$_g$ r-value			all meta	
						beta	p-value	Main	Carib	All	Main	Carib	All	Main	Carib	All	beta	p-value
Hoffmann	rs507666	9	136149399	PP	G	-0.14	8.30E-06	-0.31	0.19	-0.09	2.39E-01	5.20E-01	6.58E-01	8.15E-01	1.00	1.00E+00	-0.09	6.58E-01
Ehret '16	rs6271a	9	136522274	SBP	T	-0.59	4.89E-11	1.07	-0.23	0.42	1.38E-01	7.54E-01	4.10E-01	1.00E+00	1.00	1.00E+00	-0.56	2.40E-10
Ehret '16	rs6271a	9	136522274	DBP	T	-0.46	2.42E-18	0.51	-0.61	-0.06	2.62E-01	1.71E-01	8.54E-01	1.00E+00	0.45	1.00E+00	-0.45	5.43E-18
Hoffmann	rs10751962	10	4172711	DBP	C	-0.22	4.60E-08	0.28	-0.03	0.13	3.49E-01	9.26E-01	5.43E-01	1.00E+00	1.00	1.00E+00	0.13	5.43E-01
Hoffmann	rs12248718	10	12242326	SBP	A	-0.21	6.50E-07	0.18	-0.13	0.06	5.41E-01	7.28E-01	7.95E-01	1.00E+00	1.00	1.00E+00	0.06	7.95E-01
Hoffmann	rs12248718	10	12242326	PP	A	-0.15	4.60E-08	0.07	-0.23	-0.05	7.13E-01	3.49E-01	7.62E-01	1.00E+00	0.94	1.00E+00	-0.05	7.62E-01
Ehret '11	rs4373814	10	18419972	DBP	G	-0.22	4.40E-10	-0.13	-0.01	-0.08	4.47E-01	9.53E-01	5.38E-01	5.89E-01	1.00	6.46E-01	-0.21	5.87E-10
Ehret '11	rs4373814	10	18419972	SBP	G	-0.37	4.80E-11	0.00	0.05	0.02	9.90E-01	8.83E-01	9.19E-01	1.00E+00	1.00	1.00E+00	-0.35	2.36E-10
Ehret '11	rs1813353	10	18707448	DBP	T	0.41	2.30E-15	0.29	0.14	0.22	1.67E-01	5.35E-01	1.52E-01	2.98E-01	0.78	2.46E-01	0.39	1.68E-15
Ehret '11	rs1813353	10	18707448	SBP	T	0.57	2.60E-12	0.43	0.11	0.29	1.97E-01	7.69E-01	2.49E-01	3.82E-01	1.00	4.25E-01	0.54	2.42E-12
Levy	rs11014166	10	18708798	DBP	T	-0.46	8.70E-07	-0.15	-0.22	-0.19	4.92E-01	3.39E-01	2.51E-01	5.90E-01	0.41	2.83E-01	-0.39	5.07E-07
Hoffmann	rs73605614	10	20529470	PP	A	-0.18	1.20E-08	-0.26	0.10	-0.12	2.16E-01	7.01E-01	4.77E-01	8.07E-01	1.00	9.48E-01	-0.12	4.77E-01
Hoffmann	rs1966203	10	21057545	PP	C	-0.14	1.80E-08	-0.05	0.24	0.07	7.84E-01	2.70E-01	6.20E-01	1.00E+00	1.00	1.00E+00	0.07	6.20E-01
Hoffmann	rs9337951	10	30317073	PP	G	-0.27	1.00E-19	0.25	-0.02	0.13	2.90E-01	9.33E-01	4.70E-01	1.00E+00	1.00	1.00E+00	0.13	4.70E-01
Hoffmann	rs11008355	10	31412561	PP	G	0.17	9.90E-09	-0.11	-0.06	-0.08	6.20E-01	8.10E-01	6.08E-01	1.00E+00	1.00	1.00E+00	-0.08	6.08E-01
Hoffmann	rs813412	10	32284825	SBP	C	0.20	6.50E-06	0.11	-0.15	0.01	7.28E-01	7.10E-01	9.81E-01	1.00E+00	1.00	1.00E+00	0.01	9.81E-01
Hoffmann	rs813412	10	32284825	PP	C	0.18	2.50E-09	0.26	-0.12	0.11	2.20E-01	6.50E-01	5.23E-01	8.07E-01	1.00	9.48E-01	0.11	5.23E-01
Hoffmann	rs2246438	10	45273079	DBP	G	0.16	1.40E-09	-0.28	0.28	0.02	2.47E-01	2.09E-01	9.09E-01	1.00E+00	0.85	1.00E+00	0.02	9.09E-01
Hoffmann	rs2393455	10	60374898	PP	C	-0.15	4.90E-09	-0.39	-0.27	-0.34	5.73E-02	2.09E-01	2.60E-02	5.44E-01	0.83	3.10E-01	-0.34	2.60E-02
Hoffmann	rs2440907	10	61638804	SBP	G	-0.20	2.30E-07	-0.21	0.29	-0.01	4.44E-01	3.94E-01	9.64E-01	1.00E+00	1.00	1.00E+00	-0.01	9.64E-01
Hoffmann	rs2440907	10	61638804	PP	G	-0.14	4.40E-08	-0.22	0.27	-0.02	2.29E-01	2.16E-01	8.98E-01	8.10E-01	1.00	1.00E+00	-0.02	8.98E-01
Ehret '11	rs4590817	10	63467553	DBP	G	0.42	1.30E-12	0.38	-0.04	0.16	2.09E-01	8.96E-01	4.40E-01	3.25E-01	1.00	5.54E-01	0.40	1.94E-12
Ehret '11	rs4590817	10	63467553	SBP	G	0.65	4.00E-12	0.46	-0.34	0.05	3.38E-01	4.73E-01	8.75E-01	5.15E-01	1.00	9.76E-01	0.60	1.70E-11

Table S8: Generalization of previously reported loci associated with quantitative BP traits.

Ref	rsID	chr	position	trait	A1	discovery		HCHS/SOL beta			HCHS/SOL p-value			FDR$_g$ r-value			all meta	
						beta	p-value	Main	Carib	All	Main	Carib	All	Main	Carib	All	beta	p-value
Newton-Chen	rs1530440	10	63524591	DBP	T	-0.39	1.00E-09	-0.06	0.26	0.04	7.64E-01	3.77E-01	8.09E-01	7.64E-01	1.00	1.00E+00	-0.34	1.62E-09
Hoffmann	rs1848797	10	64552934	SBP	A	0.27	1.40E-11	0.17	-0.12	0.04	6.19E-01	7.54E-01	8.62E-01	1.00E+00	1.00	1.00E+00	0.04	8.62E-01
Hoffmann	rs1848797	10	64552934	DBP	A	0.19	1.40E-15	0.23	0.04	0.15	2.81E-01	8.78E-01	3.64E-01	9.30E-01	1.00	7.96E-01	0.15	3.64E-01
Liu	rs10995311	10	64564934	DBP	G	-0.20	2.10E-11	-0.10	-0.06	-0.08	6.39E-01	7.90E-01	5.99E-01	1.00E+00	1.00	1.00E+00	-0.20	3.02E-11
Hoffmann	rs6479908	10	65333648	DBP	C	-0.14	1.40E-09	-0.18	-0.39	-0.27	3.15E-01	6.55E-02	5.02E-02	9.30E-01	0.61	3.20E-01	-0.27	5.02E-02
Hoffmann	rs7914287	10	69350563	SBP	T	-0.20	8.20E-06	0.19	-0.80	-0.20	5.26E-01	3.04E-02	3.94E-01	1.00E+00	0.56	7.89E-01	-0.20	3.94E-01
Hoffmann	rs7914287	10	69350563	PP	T	-0.19	5.70E-10	0.02	-0.61	-0.23	9.27E-01	9.98E-03	1.25E-01	1.00E+00	0.33	4.96E-01	-0.23	1.25E-01
Hoffmann	rs2049814	10	89787275	PP	A	-0.15	1.80E-09	0.05	-0.04	0.01	7.77E-01	8.40E-01	9.31E-01	1.00E+00	1.00	1.00E+00	0.01	9.31E-01
Ehret '11	rs932764	10	95895940	DBP	G	0.18	8.10E-07	0.24	0.02	0.15	1.75E-01	9.38E-01	2.79E-01	2.98E-01	1.00	4.05E-01	0.18	4.54E-07
Ehret '11	rs932764	10	95895940	SBP	G	0.48	7.10E-16	0.15	0.01	0.09	6.01E-01	9.75E-01	6.77E-01	7.92E-01	1.00	8.54E-01	0.46	2.76E-15
Liu	rs4494250	10	96563757	DBP	A	0.15	3.40E-07	0.05	0.35	0.16	7.62E-01	1.32E-01	2.52E-01	1.00E+00	0.51	6.79E-01	0.15	2.92E-07
Hoffmann	rs4110517	10	96650328	SBP	A	-0.28	1.10E-09	-0.81	-0.51	-0.68	1.34E-02	1.83E-01	6.19E-03	3.46E-01	0.83	2.51E-01	-0.68	6.19E-03
Hoffmann	rs4110517	10	96650328	DBP	A	-0.14	1.90E-06	-0.62	-0.43	-0.54	2.32E-03	6.79E-02	4.81E-04	1.81E-01	0.61	8.51E-02	-0.54	4.81E-04
Hoffmann	rs4110517	10	96650328	PP	A	-0.15	1.70E-06	-0.19	-0.08	-0.14	3.83E-01	7.33E-01	3.79E-01	1.00E+00	1.00	8.53E-01	-0.14	3.79E-01
Hoffmann	rs4551692	10	102556453	SBP	G	-0.42	1.60E-11	-0.81	0.02	-0.44	1.19E-01	9.68E-01	2.57E-01	6.45E-01	1.00	6.61E-01	-0.44	2.57E-01
Hoffmann	rs4551692	10	102556453	DBP	G	-0.26	1.30E-11	-0.59	-0.06	-0.35	6.94E-02	8.72E-01	1.46E-01	5.49E-01	1.00	5.47E-01	-0.35	1.46E-01
HMG	rs4409766	10	104616663	DBP	T	0.59	5.69E-13	0.81	0.36	0.65	1.64E-04	2.13E-01	1.75E-04	3.11E-03	0.42	1.11E-03	0.60	4.41E-14
HMG	rs4409766	10	104616663	SBP	T	1.24	6.08E-17	1.03	0.39	0.80	2.63E-03	4.03E-01	3.70E-03	1.66E-02	0.64	1.41E-02	1.14	5.22E-18
Ehret '11	rs11191548	10	104846178	DBP	T	0.46	9.40E-13	0.84	0.46	0.73	1.05E-04	1.91E-01	7.09E-05	1.52E-03	0.43	6.85E-04	0.49	8.07E-16
Ehret '11	rs11191548	10	104846178	SBP	T	1.09	6.90E-26	1.09	0.57	0.95	1.64E-03	3.08E-01	1.35E-03	1.19E-02	0.66	9.79E-03	1.08	4.34E-28
Newton-Chen	rs11191548	10	104846178	SBP	T	1.16	7.00E-24	1.09	0.57	0.95	1.64E-03	3.08E-01	1.35E-03	3.28E-03	0.31	2.70E-03	1.13	2.92E-24

Table S8: Generalization of previously reported loci associated with quantitative BP traits.

Ref	rsID	chr	position	trait	A1	discovery		HCHS/SOL beta			HCHS/SOL p-value			FDR$_g$ r-value			all meta	
						beta	p-value	Main	Carib	All	Main	Carib	All	Main	Carib	All	beta	p-value
Hoffmann		10	104957628	PP	AT	0.62	4.50E-18	2.91	0.63	1.09	4.60E-01	7.52E-01	5.38E-01	1.00E+00	1.00	9.50E-01	0.62	3.85E-18
Hoffmann	rs34872471	10	114754071	SBP	T	-0.25	3.70E-09	0.04	-0.35	-0.14	8.97E-01	3.28E-01	5.64E-01	1.00E+00	0.94	9.23E-01	-0.14	5.64E-01
Hoffmann	rs34872471	10	114754071	PP	T	-0.20	5.40E-13	-0.07	-0.26	-0.16	7.43E-01	2.57E-01	3.09E-01	1.00E+00	0.83	7.96E-01	-0.16	3.09E-01
Hoffmann	rs17617337	10	121426884	DBP	C	0.17	3.10E-09	-0.16	0.08	-0.05	5.75E-01	8.07E-01	8.06E-01	1.00E+00	1.00	1.00E+00	-0.05	8.06E-01
Franceschini	rs11041530	11	7701503	SBP	C	1.35	5.60E-06	-0.12	0.43	0.06	8.10E-01	5.48E-01	8.88E-01	1.00E+00	1.00	1.00E+00	0.90	1.73E-04
Franceschini	rs11041530	11	7701503	DBP	C	0.54	7.60E-04	0.01	0.33	0.11	9.79E-01	4.58E-01	6.59E-01	1.00E+00	1.00	1.00E+00	0.42	2.02E-03
Hoffmann	rs360158	11	9753601	SBP	G	-0.30	1.40E-14	-0.27	-0.43	-0.34	3.64E-01	2.19E-01	1.40E-01	9.55E-01	0.87	6.36E-01	-0.34	1.40E-01
Hoffmann	rs360158	11	9753601	DBP	G	-0.17	4.70E-12	-0.11	-0.27	-0.17	5.53E-01	2.25E-01	2.19E-01	1.00E+00	0.86	6.23E-01	-0.17	2.19E-01
Hoffmann	rs360158	11	9753601	PP	G	-0.13	6.80E-07	-0.12	-0.18	-0.15	5.21E-01	4.31E-01	3.19E-01	1.00E+00	1.00	8.04E-01	-0.15	3.19E-01
Ehret '11	rs7129220	11	10350538	DBP	G	-0.30	6.40E-08	0.21	-0.67	-0.26	5.73E-01	5.38E-02	3.12E-01	1.00E+00	0.19	4.11E-01	-0.30	3.83E-08
Ehret '11	rs7129220	11	10350538	SBP	G	-0.62	3.00E-12	-0.07	-0.97	-0.55	9.09E-01	8.16E-02	1.81E-01	9.77E-01	0.34	3.43E-01	-0.62	1.23E-12
Liu	rs900145	11	13293905	DBP	G	-0.20	1.80E-08	0.03	-0.39	-0.15	8.86E-01	7.02E-02	2.91E-01	1.00E+00	0.51	6.79E-01	-0.20	1.59E-11
Hoffmann	rs7928655	11	13300252	DBP	C	-0.14	1.60E-08	0.01	-0.41	-0.17	9.57E-01	5.49E-02	2.32E-01	1.00E+00	0.61	6.23E-01	-0.17	2.32E-01
Hoffmann	rs12787709	11	14639257	SBP	G	-0.22	1.20E-07	-2.68	-2.81	-2.73	3.23E-01	4.45E-01	2.12E-01	9.42E-01	1.00	6.58E-01	-2.73	2.12E-01
Hoffmann	rs12787709	11	14639257	DBP	G	-0.14	3.40E-08	-0.20	-0.99	-0.49	9.04E-01	6.62E-01	7.20E-01	1.00E+00	1.00	1.00E+00	-0.49	7.20E-01
Franceschini	rs1401454	11	16250183	SBP	T	0.55	9.50E-07	0.34	-0.00	0.20	2.19E-01	9.94E-01	3.47E-01	1.00E+00	1.00	8.10E-01	0.48	1.76E-06
Franceschini	rs1401454	11	16250183	DBP	T	0.45	5.10E-10	0.13	0.03	0.09	4.43E-01	8.96E-01	5.03E-01	6.35E-01	1.00	8.80E-01	0.37	7.24E-09
HMG	rs4757391	11	16302939	DBP	C	0.49	4.95E-09	0.02	0.20	0.08	9.28E-01	4.14E-01	5.81E-01	1.00E+00	0.61	7.89E-01	0.38	8.25E-07
HMG	rs4757391	11	16302939	SBP	C	0.88	5.20E-09	0.14	0.45	0.24	6.40E-01	2.60E-01	3.01E-01	8.68E-01	0.49	4.77E-01	0.70	3.73E-08
Ehret '11	rs381815	11	16902268	DBP	T	0.35	5.30E-10	0.35	0.27	0.32	7.45E-02	2.63E-01	3.74E-02	1.66E-01	0.46	7.75E-02	0.34	5.89E-11
Ehret '11	rs381815	11	16902268	SBP	T	0.57	5.30E-11	0.52	0.38	0.47	9.64E-02	3.21E-01	5.63E-02	3.36E-01	0.66	1.50E-01	0.56	9.02E-12
Levy	rs381815	11	16902268	DBP	T	0.51	4.30E-07	0.35	0.27	0.32	7.45E-02	2.63E-01	3.74E-02	1.37E-01	0.36	5.46E-02	0.45	6.45E-08
Levy	rs381815	11	16902268	SBP	T	0.84	5.80E-07	0.52	0.38	0.47	9.64E-02	3.21E-01	5.63E-02	2.16E-01	0.41	9.19E-02	0.72	2.66E-07

Table S8: Generalization of previously reported loci associated with quantitative BP traits.

Ref	rsID	chr	position	trait	A1	discovery		HCHS/SOL beta			HCHS/SOL p-value			FDR$_g$ r-value			all meta	
						beta	p-value	Main	Carib	All	Main	Carib	All	Main	Carib	All	beta	p-value
Levy	rs11024074	11	16917219	DBP	C	0.50	2.80E-07	0.35	0.26	0.31	6.93E-02	2.64E-01	3.56E-02	1.35E-01	0.36	5.42E-02	0.44	1.05E-07
Levy	rs7926335	11	16917869	DBP	T	0.51	4.80E-07	0.37	0.27	0.33	5.97E-02	2.51E-01	2.98E-02	1.31E-01	0.36	4.73E-02	0.45	5.01E-08
Levy	rs7926335	11	16917869	SBP	T	0.85	5.80E-07	0.54	0.36	0.47	8.25E-02	3.34E-01	5.16E-02	2.16E-01	0.41	9.19E-02	0.72	1.90E-07
Liu	rs5219	11	17409572	SBP	T	0.32	4.90E-12	0.44	-0.18	0.23	1.19E-01	6.36E-01	3.32E-01	3.18E-01	1.00	4.77E-01	0.32	1.04E-10
Hoffmann	rs11030119	11	27728102	SBP	G	0.20	3.20E-06	0.81	0.17	0.52	1.75E-02	6.56E-01	4.08E-02	3.46E-01	1.00	3.67E-01	0.52	4.08E-02
Hoffmann	rs11030119	11	27728102	DBP	G	0.18	2.30E-12	0.48	0.16	0.33	2.33E-02	4.88E-01	3.34E-02	3.57E-01	1.00	3.09E-01	0.33	3.34E-02
Hoffmann	rs2585810	11	28483787	SBP	G	-0.22	2.70E-08	-0.20	-0.25	-0.23	5.58E-01	4.66E-01	3.55E-01	1.00E+00	1.00	7.72E-01	-0.23	3.55E-01
Hoffmann	rs2585810	11	28483787	DBP	G	-0.12	1.40E-06	-0.26	-0.28	-0.27	2.30E-01	1.84E-01	7.34E-02	8.39E-01	0.83	4.01E-01	-0.27	7.34E-02
Hoffmann	rs61879810	11	31821467	SBP	A	0.28	7.40E-08	0.02	1.06	0.33	9.45E-01	4.37E-02	2.51E-01	1.00E+00	0.56	6.61E-01	0.33	2.51E-01
Hoffmann	rs61879810	11	31821467	DBP	A	0.20	1.20E-09	-0.09	0.23	0.00	6.69E-01	4.93E-01	9.88E-01	1.00E+00	1.00	1.00E+00	0.00	9.88E-01
Hoffmann	rs74482535	11	44030783	PP	C	0.27	1.00E-09	0.51	0.40	0.46	1.36E-01	2.87E-01	6.97E-02	6.56E-01	0.86	4.06E-01	0.46	6.97E-02
Hoffmann	rs10838433	11	45233473	PP	G	-0.20	5.30E-12	0.13	-0.45	-0.11	4.85E-01	4.10E-02	4.43E-01	1.00E+00	0.44	9.04E-01	-0.11	4.43E-01
Ehret '16	rs7103648	11	47461783	SBP	A	-0.33	4.43E-13	-0.23	-0.02	-0.14	4.81E-01	9.47E-01	5.71E-01	7.81E-01	1.00	8.25E-01	-0.33	4.90E-13
Ehret '16	rs7103648	11	47461783	DBP	A	-0.24	9.03E-19	-0.12	-0.10	-0.11	5.49E-01	6.67E-01	4.64E-01	1.00E+00	1.00	8.62E-01	-0.24	8.97E-19
Hoffmann	rs7107356	11	47676170	SBP	A	-0.31	4.00E-16	-0.27	-0.32	-0.29	3.82E-01	3.42E-01	2.01E-01	9.70E-01	0.95	6.58E-01	-0.29	2.01E-01
Hoffmann	rs7107356	11	47676170	DBP	A	-0.15	1.70E-10	-0.03	-0.25	-0.13	8.95E-01	2.35E-01	3.72E-01	1.00E+00	0.87	8.01E-01	-0.13	3.72E-01
Hoffmann	rs7107356	11	47676170	PP	A	-0.16	3.30E-10	-0.24	-0.03	-0.14	2.36E-01	8.77E-01	3.31E-01	8.15E-01	1.00	8.09E-01	-0.14	3.31E-01
Hoffmann	rs61448762	11	48923756	SBP	G	0.36	3.50E-09	0.57	0.20	0.45	1.96E-01	7.52E-01	2.18E-01	8.13E-01	1.00	6.58E-01	0.45	2.18E-01
Hoffmann	rs61448762	11	48923756	DBP	G	0.17	3.20E-06	0.04	0.24	0.11	8.80E-01	5.28E-01	6.22E-01	1.00E+00	1.00	1.00E+00	0.11	6.22E-01
Hoffmann	rs74237369	11	55355182	SBP	G	0.32	4.50E-08	0.48	0.53	0.50	2.67E-01	3.95E-01	1.65E-01	8.47E-01	1.00	6.36E-01	0.50	1.65E-01
Hoffmann	rs74237369	11	55355182	DBP	G	0.16	4.30E-06	0.21	0.45	0.29	4.40E-01	2.43E-01	1.95E-01	1.00E+00	0.87	6.02E-01	0.29	1.95E-01
Hoffmann	rs685149	11	57657413	SBP	A	-0.31	1.50E-13	-0.26	-0.53	-0.39	4.95E-01	1.76E-01	1.57E-01	1.00E+00	0.82	6.36E-01	-0.39	1.57E-01
Hoffmann	rs685149	11	57657413	PP	A	-0.19	9.70E-12	-0.23	-0.54	-0.38	3.62E-01	3.14E-02	3.27E-02	1.00E+00	0.44	3.10E-01	-0.38	3.27E-02

Table S8: Generalization of previously reported loci associated with quantitative BP traits.

Ref	rsID	chr	position	trait	A1	discovery		HCHS/SOL beta			HCHS/SOL p-value			FDR$_g$ r-value			all meta	
						beta	p-value	Main	Carib	All	Main	Carib	All	Main	Carib	All	beta	p-value
Hoffmann	rs1938598	11	58413910	SBP	T	0.33	1.10E-13	0.18	0.26	0.21	6.27E-01	5.30E-01	4.39E-01	1.00E+00	1.00	8.20E-01	0.21	4.39E-01
Hoffmann	rs1938598	11	58413910	DBP	T	0.13	3.20E-06	0.23	0.08	0.17	3.06E-01	7.50E-01	3.29E-01	9.30E-01	1.00	7.79E-01	0.17	3.29E-01
Hoffmann	rs1938598	11	58413910	PP	T	0.21	1.20E-12	-0.06	0.18	0.04	8.13E-01	5.12E-01	8.01E-01	1.00E+00	1.00	1.00E+00	0.04	8.01E-01
Kato	rs751984	11	61278246	MAP	T	0.33	7.70E-12	0.31	0.67	0.42	1.45E-01	3.63E-02	1.87E-02	4.35E-01	0.11	5.60E-02	0.34	2.78E-12
Ehret '16	rs751984	11	61278246	SBP	T	0.41	3.80E-09	0.50	0.91	0.62	1.03E-01	4.47E-02	1.45E-02	3.36E-01	0.43	1.89E-01	0.42	2.47E-10
Ehret '16	rs751984	11	61278246	DBP	T	0.38	4.20E-20	0.22	0.54	0.32	2.42E-01	5.30E-02	4.21E-02	6.58E-01	0.34	4.71E-01	0.37	5.55E-21
Hoffmann	rs72930293	11	69073420	DBP	C	0.23	1.70E-09	-0.08	-0.62	-0.33	8.21E-01	8.83E-02	1.84E-01	1.00E+00	1.00	1.00E+00	-0.33	1.84E-01
Hoffmann	rs7927515	11	76125330	SBP	C	-0.23	4.40E-09	0.04	-0.37	-0.17	9.08E-01	2.80E-01	4.98E-01	1.00E+00	0.89	8.65E-01	-0.17	4.98E-01
Hoffmann	rs7927515	11	76125330	PP	C	-0.15	2.60E-08	0.20	-0.16	0.02	3.84E-01	4.69E-01	9.23E-01	1.00E+00	1.00	1.00E+00	0.02	9.23E-01
Hoffmann	rs2289125	11	89224453	SBP	A	-0.27	1.60E-09	-0.00	-0.54	-0.21	9.90E-01	1.40E-01	3.53E-01	1.00E+00	0.70	7.72E-01	-0.21	3.53E-01
Hoffmann	rs2289125	11	89224453	PP	A	-0.35	1.50E-30	-0.20	-0.27	-0.23	3.02E-01	2.50E-01	1.29E-01	9.66E-01	0.83	4.96E-01	-0.23	1.29E-01
Hoffmann	rs11021221	11	95308854	DBP	T	0.21	2.70E-11	0.05	0.15	0.09	8.70E-01	6.87E-01	7.02E-01	1.00E+00	1.00	1.00E+00	0.09	7.02E-01
Ehret '11	rs633185	11	100593538	DBP	G	-0.33	2.00E-15	-0.14	-0.38	-0.23	4.41E-01	1.02E-01	1.10E-01	5.89E-01	0.33	1.87E-01	-0.32	6.87E-16
Ehret '11	rs633185	11	100593538	SBP	G	-0.56	1.20E-17	-0.17	-0.02	-0.11	5.61E-01	9.49E-01	6.15E-01	7.75E-01	1.00	8.49E-01	-0.53	6.49E-17
Hoffmann	rs7951348	11	107081841	SBP	C	-0.25	3.60E-11	-0.25	-0.23	-0.24	3.70E-01	5.01E-01	2.69E-01	9.55E-01	1.00	6.83E-01	-0.24	2.69E-01
Hoffmann	rs7951348	11	107081841	DBP	C	-0.12	2.10E-07	-0.16	0.18	-0.02	3.76E-01	3.99E-01	8.85E-01	1.00E+00	1.00	1.00E+00	-0.02	8.85E-01
Hoffmann	rs7951348	11	107081841	PP	C	-0.12	1.40E-06	-0.10	-0.44	-0.24	6.00E-01	5.00E-02	1.02E-01	1.00E+00	0.46	4.70E-01	-0.24	1.02E-01
Hoffmann	rs115381894	11	109019018	SBP	G	-5.21	3.70E-08	-1.61	1.25	0.42	6.74E-01	6.10E-01	8.40E-01	1.00E+00	1.00	1.00E+00	0.42	8.40E-01
Hoffmann	rs5794844	11	112960099	SBP	GT	-0.35	3.50E-08	-0.31	-0.26	-0.29	2.63E-01	4.42E-01	1.78E-01	8.47E-01	1.00	6.58E-01	-0.29	1.78E-01
Hoffmann	rs5794844	11	112960099	PP	GT	-0.27	3.10E-09	0.01	-0.31	-0.12	9.48E-01	1.59E-01	4.00E-01	1.00E+00	0.75	8.71E-01	-0.12	4.00E-01
Hoffmann	rs7116797	11	116707338	SBP	A	0.31	6.20E-08	-0.14	0.66	0.17	6.65E-01	1.06E-01	4.99E-01	1.00E+00	0.66	8.65E-01	0.17	4.99E-01
Hoffmann	rs7116797	11	116707338	DBP	A	0.21	1.60E-09	-0.13	0.46	0.11	5.27E-01	6.48E-02	5.00E-01	1.00E+00	0.61	9.44E-01	0.11	5.00E-01
Hoffmann	rs1261744	11	117218460	PP	T	-0.26	7.30E-17	0.07	-0.04	0.03	7.16E-01	8.84E-01	8.46E-01	1.00E+00	1.00	1.00E+00	0.03	8.46E-01

Table S8: Generalization of previously reported loci associated with quantitative BP traits.

Ref	rsID	chr	position	trait	A1	discovery		HCHS/SOL beta			HCHS/SOL p-value			FDR$_g$ r-value			all meta	
						beta	p-value	Main	Carib	All	Main	Carib	All	Main	Carib	All	beta	p-value
Hoffmann	rs117204111	11	118199425	PP	G	0.49	3.00E-08	3.22	0.70	2.10	3.53E-03	5.73E-01	1.07E-02	3.49E-01	1.00	2.36E-01	2.10	1.07E-02
Hoffmann	rs11222386	11	130779068	PP	G	-0.18	4.40E-08	-0.55	0.26	-0.14	7.23E-02	3.90E-01	5.05E-01	5.60E-01	1.00	9.48E-01	-0.14	5.05E-01
Hoffmann	rs4980877	12	418916	PP	C	-0.16	2.70E-08	0.11	-0.12	0.02	5.50E-01	6.23E-01	8.74E-01	1.00E+00	1.00	1.00E+00	0.02	8.74E-01
Hoffmann	rs143750586	12	4358078	PP	A	1.04	1.40E-08	-2.30	-3.87	-3.24	2.82E-01	2.61E-02	1.62E-02	1.00E+00	1.00	1.00E+00	-3.24	1.62E-02
Kato	rs12579720	12	20173764	DBP	C	0.32	2.20E-16	-0.38	-0.34	-0.36	4.69E-02	1.21E-01	1.22E-02	1.00E+00	1.00	1.00E+00	0.27	1.91E-12
Hoffmann	rs11168244	12	48202941	SBP	C	0.32	2.10E-10	0.25	-0.14	0.06	5.80E-01	7.63E-01	8.54E-01	1.00E+00	1.00	1.00E+00	0.06	8.54E-01
Hoffmann	rs11168244	12	48202941	DBP	C	0.17	4.70E-08	0.18	-0.18	-0.00	5.41E-01	5.26E-01	9.88E-01	1.00E+00	1.00	1.00E+00	-0.00	9.88E-01
Hoffmann	rs7977389	12	49981722	SBP	T	0.38	3.30E-10	0.67	0.31	0.50	1.21E-01	4.82E-01	1.10E-01	6.45E-01	1.00	6.36E-01	0.50	1.10E-01
Hoffmann	rs7977389	12	49981722	PP	T	0.27	1.10E-11	0.49	0.05	0.27	8.70E-02	8.57E-01	1.81E-01	5.60E-01	1.00	5.78E-01	0.27	1.81E-01
Hoffmann	rs10747570	12	50509937	SBP	A	0.26	1.40E-10	0.13	0.00	0.08	6.70E-01	9.89E-01	7.45E-01	1.00E+00	1.00	1.00E+00	0.08	7.45E-01
Hoffmann	rs10747570	12	50509937	DBP	A	0.18	4.30E-14	0.13	0.23	0.17	4.94E-01	3.07E-01	2.46E-01	1.00E+00	0.90	6.39E-01	0.17	2.46E-01
Liu	rs7302981	12	50537815	DBP	A	0.25	9.40E-19	0.22	0.27	0.24	2.72E-01	2.36E-01	1.14E-01	9.51E-01	0.66	5.30E-01	0.25	2.21E-17
Hoffmann	rs17210898	12	51056511	SBP	G	0.56	3.50E-08	-0.15	0.28	0.09	9.02E-01	7.95E-01	9.10E-01	1.00E+00	1.00	1.00E+00	0.09	9.10E-01
Hoffmann	rs17210898	12	51056511	DBP	G	0.35	1.10E-08	-0.05	-0.10	-0.08	9.44E-01	8.86E-01	8.77E-01	1.00E+00	1.00	1.00E+00	-0.08	8.77E-01
Hoffmann	rs10784502	12	66343810	SBP	C	-0.23	7.40E-10	-0.13	-0.25	-0.18	6.70E-01	4.69E-01	4.26E-01	1.00E+00	1.00	8.20E-01	-0.18	4.26E-01
Hoffmann	rs10784502	12	66343810	PP	C	-0.19	1.30E-13	-0.15	-0.07	-0.12	4.20E-01	7.47E-01	4.13E-01	1.00E+00	1.00	8.71E-01	-0.12	4.13E-01
Hoffmann	rs1152958	12	70325669	DBP	G	0.14	4.00E-08	-0.03	0.01	-0.01	8.99E-01	9.52E-01	9.57E-01	1.00E+00	1.00	1.00E+00	-0.01	9.57E-01
Levy	rs4842666	12	89941549	DBP	C	-0.62	4.50E-07	-0.16	-0.80	-0.41	4.78E-01	4.49E-03	2.01E-02	5.90E-01	0.02	3.35E-02	-0.56	2.37E-08
Levy	rs4842666	12	89941549	SBP	C	-1.20	6.50E-09	-0.67	-0.80	-0.72	6.55E-02	7.99E-02	1.17E-02	2.16E-01	0.30	7.11E-02	-1.03	1.08E-09
Levy	rs11105328	12	89942390	DBP	G	-0.61	5.10E-07	-0.48	-0.87	-0.67	1.13E-01	3.64E-03	1.58E-03	1.73E-01	0.02	1.46E-02	-0.63	2.24E-09
Levy	rs11105328	12	89942390	SBP	G	-1.11	4.20E-08	-0.82	-0.91	-0.86	8.86E-02	6.02E-02	1.19E-02	2.16E-01	0.30	7.11E-02	-1.05	1.34E-09
Levy	rs2681472	12	90008959	DBP	G	-0.64	3.70E-08	-0.66	-0.89	-0.76	1.76E-02	3.21E-03	2.01E-04	9.13E-02	0.02	4.66E-03	-0.67	9.17E-11
Levy	rs2681472	12	90008959	SBP	G	-1.29	3.50E-11	-0.72	-0.93	-0.81	1.06E-01	5.74E-02	1.41E-02	2.16E-01	0.30	7.11E-02	-1.17	1.16E-12

Table S8: Generalization of previously reported loci associated with quantitative BP traits.

Ref	rsID	chr	position	trait	A1	discovery		HCHS/SOL beta			HCHS/SOL p-value			FDR$_g$ r-value			all meta	
						beta	p-value	Main	Carib	All	Main	Carib	All	Main	Carib	All	beta	p-value
Levy	rs2681492	12	90013089	DBP	C	-0.62	4.60E-08	-0.64	-0.75	-0.69	2.09E-02	1.16E-02	7.02E-04	9.13E-02	0.02	4.66E-03	-0.64	5.03E-11
Levy	rs2681492	12	90013089	SBP	C	-1.26	3.00E-11	-0.69	-0.69	-0.69	1.16E-01	1.47E-01	3.38E-02	2.16E-01	0.31	7.11E-02	-1.12	1.06E-11
Levy	rs11105354	12	90026523	DBP	G	-0.63	5.80E-08	-0.68	-0.87	-0.76	1.47E-02	3.91E-03	1.97E-04	9.13E-02	0.02	4.66E-03	-0.66	1.44E-10
Levy	rs11105354	12	90026523	SBP	G	-1.30	3.70E-11	-0.72	-0.89	-0.80	1.02E-01	6.65E-02	1.54E-02	2.16E-01	0.30	7.11E-02	-1.16	9.52E-12
Levy	rs12579302	12	90050503	DBP	G	-0.62	1.20E-07	-0.66	-0.87	-0.76	1.66E-02	3.97E-03	2.26E-04	9.13E-02	0.02	4.66E-03	-0.65	2.58E-10
Levy	rs12579302	12	90050503	SBP	G	-1.29	6.20E-11	-0.70	-0.87	-0.78	1.15E-01	7.25E-02	1.85E-02	2.16E-01	0.30	7.11E-02	-1.15	1.63E-11
Ehret '11	rs17249754	12	90060586	DBP	G	0.52	1.20E-14	0.64	0.69	0.66	2.06E-02	1.98E-02	1.12E-03	7.47E-02	0.08	4.62E-03	0.54	6.74E-17
Ehret '11	rs17249754	12	90060586	SBP	G	0.93	1.80E-18	0.68	0.55	0.62	1.23E-01	2.46E-01	5.69E-02	3.36E-01	0.66	1.50E-01	0.90	4.34E-19
HMG	rs17249754	12	90060586	DBP	G	0.52	2.13E-10	0.64	0.69	0.66	2.06E-02	1.98E-02	1.12E-03	7.83E-02	0.08	5.30E-03	0.54	4.38E-13
HMG	rs17249754	12	90060586	SBP	G	1.03	3.66E-12	0.68	0.55	0.62	1.23E-01	2.46E-01	5.69E-02	2.93E-01	0.49	1.54E-01	0.96	2.02E-12
Levy	rs17249754	12	90060586	DBP	A	-0.63	1.00E-07	-0.64	-0.69	-0.66	2.06E-02	1.98E-02	1.12E-03	9.13E-02	0.04	4.66E-03	-0.64	6.48E-10
Levy	rs17249754	12	90060586	SBP	A	-1.30	5.20E-11	-0.68	-0.55	-0.62	1.23E-01	2.46E-01	5.69E-02	2.16E-01	0.41	9.19E-02	-1.11	6.39E-11
Levy	rs11105364	12	90069276	DBP	G	-0.63	1.20E-07	-0.66	-0.83	-0.74	1.77E-02	5.44E-03	3.15E-04	9.13E-02	0.02	4.66E-03	-0.66	2.13E-10
Levy	rs11105364	12	90069276	SBP	G	-1.30	4.80E-11	-0.69	-0.80	-0.74	1.16E-01	9.90E-02	2.43E-02	2.16E-01	0.30	7.11E-02	-1.15	1.77E-11
Levy	rs11105368	12	90074441	DBP	C	-0.63	1.20E-07	-0.65	-0.78	-0.71	1.90E-02	8.83E-03	5.09E-04	9.13E-02	0.02	4.66E-03	-0.65	3.22E-10
Levy	rs11105368	12	90074441	SBP	C	-1.30	5.30E-11	-0.70	-0.71	-0.70	1.12E-01	1.42E-01	3.17E-02	2.16E-01	0.31	7.11E-02	-1.14	2.64E-11
Levy	rs11105378	12	90090741	DBP	T	-0.62	3.10E-07	-0.62	-0.84	-0.72	2.42E-02	5.30E-03	4.29E-04	9.42E-02	0.02	1.06E-02	-0.65	4.42E-10
Levy	rs11105378	12	90090741	SBP	T	-1.31	9.10E-11	-0.65	-0.80	-0.71	1.44E-01	1.01E-01	3.02E-02	2.16E-01	0.30	7.11E-02	-1.15	1.79E-11
Levy	rs12230074	12	90090867	DBP	G	-0.62	3.40E-07	-0.61	-0.81	-0.70	2.76E-02	6.30E-03	5.63E-04	9.66E-02	0.02	1.09E-02	-0.64	5.55E-10
Levy	rs12230074	12	90090867	SBP	G	-1.31	9.10E-11	-0.64	-0.75	-0.69	1.44E-01	1.16E-01	3.38E-02	2.16E-01	0.30	7.11E-02	-1.14	2.17E-11
Hoffmann	rs10859580	12	94180616	PP	A	-0.14	3.70E-08	-0.31	-0.11	-0.23	1.02E-01	6.41E-01	1.20E-01	5.60E-01	1.00	4.96E-01	-0.23	1.20E-01
Hoffmann	rs76785029	12	94882905	PP	C	0.38	5.40E-14	-0.24	-0.25	-0.25	6.33E-01	6.48E-01	5.11E-01	1.00E+00	1.00	1.00E+00	-0.25	5.11E-01
Hoffmann	rs7312132	12	110352509	SBP	G	0.35	1.40E-06	0.22	0.42	0.34	7.01E-01	4.11E-01	3.84E-01	1.00E+00	1.00	7.88E-01	0.34	3.84E-01

Table S8: Generalization of previously reported loci associated with quantitative BP traits.

Ref	rsID	chr	position	trait	A1	discovery		HCHS/SOL beta			HCHS/SOL p-value			FDR$_g$ r-value			all meta	
						beta	p-value	Main	Carib	All	Main	Carib	All	Main	Carib	All	beta	p-value
Hoffmann	rs7312132	12	110352509	PP	G	0.28	6.80E-09	0.24	0.00	0.11	5.21E-01	9.95E-01	6.74E-01	1.00E+00	1.00	1.00E+00	0.11	6.74E-01
Ehret '11	rs3184504	12	111884608	DBP	T	0.45	3.60E-25	0.41	0.67	0.53	5.77E-02	4.06E-03	9.38E-04	1.43E-01	0.02	4.62E-03	0.45	1.63E-27
Ehret '11	rs3184504	12	111884608	SBP	T	0.60	3.80E-18	0.15	0.39	0.26	6.56E-01	2.99E-01	3.07E-01	7.95E-01	0.66	4.69E-01	0.58	4.99E-18
Levy	rs3184504	12	111884608	DBP	T	0.50	1.70E-08	0.41	0.67	0.53	5.77E-02	4.06E-03	9.38E-04	1.31E-01	0.02	4.66E-03	0.51	1.02E-10
Levy	rs3184504	12	111884608	SBP	T	0.75	5.70E-07	0.15	0.39	0.26	6.56E-01	2.99E-01	3.07E-01	9.18E-01	0.41	4.30E-01	0.63	1.34E-06
Levy	rs4766578	12	111904371	DBP	T	0.49	4.20E-08	0.42	0.65	0.53	5.05E-02	4.93E-03	9.33E-04	1.31E-01	0.02	4.66E-03	0.50	1.91E-10
Levy	rs10774625	12	111910219	DBP	A	0.49	4.20E-08	0.41	0.65	0.52	5.86E-02	5.06E-03	1.12E-03	1.31E-01	0.02	4.66E-03	0.50	2.26E-10
Levy	rs653178	12	112007756	DBP	C	0.50	2.00E-08	0.39	0.64	0.51	6.78E-02	5.61E-03	1.43E-03	1.35E-01	0.02	4.66E-03	0.50	1.50E-10
Levy	rs653178	12	112007756	SBP	C	0.74	8.50E-07	0.13	0.39	0.25	7.15E-01	3.01E-01	3.37E-01	9.39E-01	0.41	4.42E-01	0.62	2.07E-06
Levy	rs11065987	12	112072424	DBP	G	0.48	2.20E-07	0.38	0.73	0.55	8.31E-02	2.01E-03	8.55E-04	1.45E-01	0.02	7.98E-03	0.50	3.38E-10
Levy	rs17696736	12	112486818	DBP	G	0.46	5.10E-07	0.31	0.72	0.50	1.62E-01	2.41E-03	2.25E-03	2.37E-01	0.02	1.46E-02	0.47	2.67E-09
Levy	rs17630235	12	112591686	DBP	A	0.50	1.00E-07	0.35	0.77	0.54	1.14E-01	1.26E-03	9.20E-04	1.73E-01	0.02	4.66E-03	0.51	1.02E-10
Levy	rs11066188	12	112610714	DBP	A	0.50	1.10E-07	0.36	0.74	0.54	1.01E-01	2.01E-03	1.10E-03	1.69E-01	0.02	4.66E-03	0.51	1.19E-10
HMG	rs11066280	12	112817783	DBP	T	0.62	3.19E-10	-2.89	1.61	-1.46	3.00E-01	6.92E-01	5.28E-01	1.00E+00	0.88	1.00E+00	0.62	6.96E-10
HMG	rs11066280	12	112817783	SBP	T	0.96	9.80E-08	-1.65	10.42	2.09	7.13E-01	1.17E-01	5.75E-01	1.00E+00	0.32	7.45E-01	0.96	1.46E-08
Levy	rs7963771	12	115343492	DBP	T	-0.53	4.30E-07	-0.23	-0.20	-0.22	1.81E-01	3.57E-01	1.07E-01	2.54E-01	0.42	1.49E-01	-0.42	1.73E-07
HMG	rs1991391	12	115352666	DBP	G	0.21	4.52E-02	0.07	0.12	0.09	7.39E-01	5.72E-01	5.33E-01	1.00E+00	1.00	1.00E+00	0.17	5.65E-02
HMG	rs1991391	12	115352666	SBP	G	0.60	1.81E-03	-0.00	0.28	0.12	9.90E-01	4.35E-01	6.09E-01	1.00E+00	1.00	1.00E+00	0.40	8.46E-03
Levy	rs1991391	12	115352666	DBP	A	-0.48	1.40E-07	-0.07	-0.12	-0.09	7.39E-01	5.72E-01	5.33E-01	7.83E-01	0.61	5.49E-01	-0.38	1.00E-06
Levy	rs2384550	12	115352731	DBP	A	-0.48	1.30E-07	-0.09	-0.16	-0.13	6.42E-01	4.56E-01	4.01E-01	7.02E-01	0.50	4.25E-01	-0.39	5.42E-07
Levy	rs6489992	12	115352769	DBP	A	-0.48	2.00E-07	-0.10	-0.18	-0.14	5.89E-01	3.91E-01	3.36E-01	6.65E-01	0.44	3.68E-01	-0.38	5.15E-07
Levy	rs10744835	12	115353849	DBP	A	-0.49	7.10E-07	-0.14	-0.37	-0.25	5.06E-01	1.02E-01	1.12E-01	5.90E-01	0.17	1.50E-01	-0.42	6.17E-07
Levy	rs7977406	12	115359424	DBP	A	-0.49	7.60E-07	-0.06	-0.36	-0.20	7.67E-01	1.07E-01	1.90E-01	7.90E-01	0.17	2.22E-01	-0.41	1.40E-06

Table S8: Generalization of previously reported loci associated with quantitative BP traits.

Ref	rsID	chr	position	trait	A1	discovery		HCHS/SOL beta			HCHS/SOL p-value			FDR$_g$ r-value			all meta	
						beta	p-value	Main	Carib	All	Main	Carib	All	Main	Carib	All	beta	p-value
Ehret '11	rs10850411	12	115387796	DBP	T	0.25	5.40E-10	0.42	0.27	0.36	1.85E-02	2.29E-01	1.00E-02	7.47E-02	0.45	2.91E-02	0.26	2.39E-11
Ehret '11	rs10850411	12	115387796	SBP	T	0.35	5.40E-08	0.76	0.58	0.69	7.70E-03	1.05E-01	2.11E-03	4.46E-02	0.38	1.22E-02	0.38	1.20E-09
HMG	rs35444	12	115552437	DBP	A	0.36	6.73E-05	0.13	0.16	0.14	4.92E-01	4.43E-01	3.09E-01	1.00E+00	1.00	1.00E+00	0.30	9.03E-05
HMG	rs35444	12	115552437	SBP	A	0.83	2.17E-07	0.26	-0.07	0.12	3.79E-01	8.40E-01	5.88E-01	7.20E-01	1.00	7.45E-01	0.59	5.59E-06
HMG	rs11067763	12	116198341	DBP	A	0.51	2.00E-18	0.63	0.33	0.52	2.55E-03	2.15E-01	1.72E-03	1.61E-02	0.42	5.86E-03	0.51	1.31E-19
HMG	rs11067763	12	116198341	SBP	A	0.81	5.68E-16	0.54	0.23	0.42	1.06E-01	5.96E-01	1.09E-01	2.88E-01	0.87	2.30E-01	0.76	3.83E-16
Hoffmann	rs7980687	12	123822711	DBP	G	0.20	1.70E-11	0.21	0.20	0.21	2.92E-01	4.39E-01	1.93E-01	9.30E-01	1.00	6.02E-01	0.21	1.93E-01
Hoffmann	rs530280439	12	127031062	PP	C	-17.47	4.80E-09	-0.40	3.59	2.75	9.58E-01	3.64E-01	4.34E-01	1.00E+00	1.00	1.00E+00	2.75	4.34E-01
Hoffmann	rs63418562	13	30146201	SBP	C	0.31	8.90E-09	-0.27	0.54	0.04	3.29E-01	1.30E-01	8.64E-01	1.00E+00	0.68	1.00E+00	0.04	8.64E-01
Hoffmann	rs63418562	13	30146201	DBP	C	0.24	1.10E-11	-0.13	0.32	0.04	4.54E-01	1.45E-01	7.46E-01	1.00E+00	0.83	1.00E+00	0.04	7.46E-01
Hoffmann	rs9565436	13	36213631	SBP	A	-0.30	2.60E-08	-0.47	0.10	-0.21	2.31E-01	8.12E-01	4.72E-01	8.26E-01	1.00	8.62E-01	-0.21	4.72E-01
Hoffmann	rs9565436	13	36213631	PP	A	-0.20	6.20E-08	-0.35	0.06	-0.16	1.71E-01	8.22E-01	3.98E-01	7.22E-01	1.00	8.71E-01	-0.16	3.98E-01
Hoffmann	rs7989823	13	110959643	DBP	A	-0.15	5.60E-09	-0.03	-0.05	-0.04	8.63E-01	8.19E-01	7.80E-01	1.00E+00	1.00	1.00E+00	-0.04	7.80E-01
Hoffmann	rs3011549	13	113634937	SBP	A	0.33	1.20E-11	0.16	-0.19	0.04	5.65E-01	6.17E-01	8.76E-01	1.00E+00	1.00	1.00E+00	0.04	8.76E-01
Hoffmann	rs3011549	13	113634937	DBP	A	0.14	2.50E-06	0.11	0.03	0.08	5.15E-01	9.00E-01	5.58E-01	1.00E+00	1.00	1.00E+00	0.08	5.58E-01
Hoffmann	rs3011549	13	113634937	PP	A	0.17	5.10E-08	0.08	-0.22	-0.03	6.69E-01	3.54E-01	8.27E-01	1.00E+00	1.00	1.00E+00	-0.03	8.27E-01
Hoffmann	rs3934939	13	114503990	DBP	A	0.16	8.40E-09	0.00	-0.26	-0.10	9.95E-01	2.19E-01	4.47E-01	1.00E+00	1.00	1.00E+00	-0.10	4.47E-01
Hoffmann	rs9314907	13	115015163	SBP	C	-0.29	1.30E-10	-0.09	-0.46	-0.28	8.13E-01	2.12E-01	2.90E-01	1.00E+00	0.87	7.05E-01	-0.28	2.90E-01
Hoffmann	rs9314907	13	115015163	DBP	C	-0.12	9.90E-06	-0.13	-0.19	-0.16	5.87E-01	4.06E-01	3.31E-01	1.00E+00	0.99	7.79E-01	-0.16	3.31E-01
Hoffmann	rs9314907	13	115015163	PP	C	-0.16	1.40E-07	0.05	-0.25	-0.11	8.56E-01	2.87E-01	5.17E-01	1.00E+00	0.86	9.48E-01	-0.11	5.17E-01
Hoffmann	rs12050260	14	23761094	SBP	T	0.21	3.20E-07	0.08	-0.41	-0.12	7.66E-01	2.22E-01	5.84E-01	1.00E+00	1.00	1.00E+00	-0.12	5.84E-01
Hoffmann	rs12050260	14	23761094	PP	T	0.19	1.60E-12	0.13	-0.12	0.03	4.83E-01	5.81E-01	8.55E-01	1.00E+00	1.00	1.00E+00	0.03	8.55E-01
Liu	rs452036	14	23865885	PP	A	-0.27	2.40E-16	-0.32	-0.57	-0.45	1.34E-01	9.02E-03	3.93E-03	2.68E-01	0.07	3.14E-02	-0.28	6.31E-21

Table S8: Generalization of previously reported loci associated with quantitative BP traits.

Ref	rsID	chr	position	trait	A1	discovery		HCHS/SOL beta			HCHS/SOL p-value			FDR$_g$ r-value			all meta	
						beta	p-value	Main	Carib	All	Main	Carib	All	Main	Carib	All	beta	p-value
Hoffmann	rs36226649	14	24835500	DBP	T	-0.30	2.60E-09	0.75	-0.23	0.29	1.23E-01	6.55E-01	4.17E-01	1.00E+00	1.00	1.00E+00	0.29	4.17E-01
Hoffmann	rs8904	14	35871217	SBP	G	-0.26	4.20E-11	0.20	0.23	0.22	5.09E-01	4.91E-01	3.44E-01	1.00E+00	1.00	1.00E+00	0.22	3.44E-01
Hoffmann	rs8904	14	35871217	PP	G	-0.17	1.50E-10	-0.04	0.11	0.03	8.56E-01	6.05E-01	8.29E-01	1.00E+00	1.00	1.00E+00	0.03	8.29E-01
Hoffmann	rs7161323	14	53366149	SBP	C	-0.28	5.70E-11	0.02	-0.53	-0.21	9.36E-01	1.32E-01	3.59E-01	1.00E+00	0.68	7.72E-01	-0.21	3.59E-01
Hoffmann	rs7161323	14	53366149	DBP	C	-0.15	1.20E-08	0.11	-0.32	-0.07	5.53E-01	1.41E-01	6.07E-01	1.00E+00	0.83	1.00E+00	-0.07	6.07E-01
Hoffmann	rs7161323	14	53366149	PP	C	-0.13	2.20E-06	-0.06	-0.21	-0.12	7.78E-01	3.51E-01	4.09E-01	1.00E+00	0.94	8.71E-01	-0.12	4.09E-01
Hoffmann	rs2215590	14	73297741	PP	C	-0.16	1.80E-08	-0.03	0.01	-0.01	8.78E-01	9.68E-01	9.21E-01	1.00E+00	1.00	1.00E+00	-0.01	9.21E-01
Hoffmann	rs2244643	14	92359022	PP	A	-0.22	4.00E-14	-0.19	-0.11	-0.15	3.23E-01	6.22E-01	2.86E-01	1.00E+00	1.00	7.77E-01	-0.15	2.86E-01
Hoffmann	rs367700296	14	98597422	PP	G	-0.27	9.80E-09	-0.16	-0.17	-0.16	4.95E-01	4.65E-01	3.21E-01	1.00E+00	1.00	8.04E-01	-0.16	3.21E-01
Hoffmann	rs1475130	14	100225144	PP	T	-0.17	6.90E-10	0.51	0.10	0.31	2.98E-02	6.60E-01	6.45E-02	1.00E+00	1.00	1.00E+00	0.31	6.45E-02
Hoffmann	rs937213	15	40322124	SBP	T	0.26	1.60E-10	0.36	-0.22	0.09	3.27E-01	5.79E-01	7.38E-01	9.42E-01	1.00	1.00E+00	0.09	7.38E-01
Hoffmann	rs937213	15	40322124	DBP	T	0.13	7.50E-08	0.26	-0.24	0.03	2.56E-01	3.26E-01	8.79E-01	8.98E-01	1.00	1.00E+00	0.03	8.79E-01
Hoffmann	rs937213	15	40322124	PP	T	0.12	4.70E-06	0.09	0.00	0.05	7.12E-01	9.90E-01	7.80E-01	1.00E+00	1.00	1.00E+00	0.05	7.80E-01
Hoffmann	rs112925537	15	41334213	SBP	A	-0.41	2.40E-10	-0.69	-0.40	-0.57	2.19E-02	2.48E-01	1.34E-02	3.46E-01	0.89	2.51E-01	-0.57	1.34E-02
Hoffmann	rs112925537	15	41334213	DBP	A	-0.20	2.10E-08	-0.37	-0.18	-0.29	5.18E-02	4.13E-01	4.61E-02	5.12E-01	0.99	3.11E-01	-0.29	4.61E-02
Hoffmann	rs112925537	15	41334213	PP	A	-0.20	8.40E-06	-0.36	-0.21	-0.30	6.43E-02	3.60E-01	4.67E-02	5.53E-01	0.95	3.10E-01	-0.30	4.67E-02
Hoffmann	rs4923910	15	42086340	DBP	G	-0.18	3.50E-13	0.10	-0.05	0.04	5.62E-01	8.32E-01	7.54E-01	1.00E+00	1.00	1.00E+00	0.04	7.54E-01
Hoffmann	rs35654783	15	44018656	DBP	T	0.17	1.30E-10	0.31	-0.12	0.13	1.01E-01	5.88E-01	3.77E-01	5.49E-01	1.00	8.01E-01	0.13	3.77E-01
Hoffmann	rs2899463	15	50938978	SBP	T	0.19	5.80E-07	-0.03	0.38	0.14	9.25E-01	2.64E-01	5.27E-01	1.00E+00	0.89	8.78E-01	0.14	5.27E-01
Hoffmann	rs2899463	15	50938978	PP	T	0.15	1.30E-09	-0.03	0.34	0.12	8.50E-01	1.18E-01	3.96E-01	1.00E+00	0.71	8.71E-01	0.12	3.96E-01
Hoffmann	rs956006	15	62808539	PP	C	0.21	2.90E-15	0.17	0.68	0.37	3.67E-01	5.04E-03	1.51E-02	1.00E+00	0.25	2.72E-01	0.37	1.51E-02
Hoffmann	rs1027647	15	63374825	PP	C	0.15	3.30E-09	0.07	0.42	0.22	7.34E-01	6.96E-02	1.50E-01	1.00E+00	0.53	5.31E-01	0.22	1.50E-01
Hoffmann	rs11638064	15	67460009	PP	G	-0.17	1.70E-08	-0.62	-0.19	-0.45	1.36E-03	4.20E-01	2.74E-03	2.69E-01	1.00	1.91E-01	-0.45	2.74E-03

Table S8: Generalization of previously reported loci associated with quantitative BP traits.

Ref	rsID	chr	position	trait	A1	discovery		HCHS/SOL beta			HCHS/SOL p-value			FDR$_g$ r-value			all meta	
						beta	p-value	Main	Carib	All	Main	Carib	All	Main	Carib	All	beta	p-value
Hoffmann	rs117638970	15	69675605	DBP	C	0.48	2.20E-08	-0.49	-0.51	-0.50	6.03E-01	5.73E-01	4.43E-01	1.00E+00	1.00	1.00E+00	-0.50	4.43E-01
Hoffmann	rs11631778	15	71606380	DBP	G	-0.14	1.10E-08	-0.45	-0.19	-0.32	4.03E-02	3.80E-01	4.04E-02	5.12E-01	0.95	3.09E-01	-0.32	4.04E-02
Ehret '11	rs1378942	15	75077367	DBP	C	0.42	2.70E-26	0.62	0.00	0.34	2.14E-03	9.98E-01	2.52E-02	2.07E-02	1.00	5.63E-02	0.41	2.48E-27
Ehret '11	rs1378942	15	75077367	SBP	C	0.61	5.70E-23	0.29	0.15	0.23	3.61E-01	6.60E-01	3.40E-01	5.23E-01	1.00	4.93E-01	0.59	1.19E-22
Newton-Chen	rs1378942	15	75077367	DBP	C	0.43	1.00E-23	0.62	0.00	0.34	2.14E-03	9.98E-01	2.52E-02	6.41E-03	1.00	7.57E-02	0.42	5.73E-28
Levy	rs6495122	15	75125645	DBP	C	0.45	8.00E-07	-0.13	-0.27	-0.19	4.57E-01	2.02E-01	1.66E-01	1.00E+00	1.00	1.00E+00	0.26	6.37E-04
Hoffmann	rs62011052	15	79156983	PP	T	-0.28	3.10E-15	-0.06	-0.06	-0.06	8.36E-01	8.56E-01	7.84E-01	1.00E+00	1.00	1.00E+00	-0.06	7.84E-01
Hoffmann	rs2759308	15	81016227	SBP	G	-0.28	1.30E-12	0.16	-0.70	-0.24	6.29E-01	5.50E-02	3.50E-01	1.00E+00	0.56	7.72E-01	-0.24	3.50E-01
Hoffmann	rs2759308	15	81016227	DBP	G	-0.14	9.00E-09	0.03	-0.24	-0.10	9.05E-01	2.96E-01	5.41E-01	1.00E+00	0.90	1.00E+00	-0.10	5.41E-01
Hoffmann	rs2759308	15	81016227	PP	G	-0.14	2.60E-08	0.12	-0.46	-0.15	5.96E-01	5.20E-02	3.48E-01	1.00E+00	0.46	8.20E-01	-0.15	3.48E-01
Hoffmann	rs2034618	15	83799632	DBP	C	0.19	2.00E-11	0.32	0.16	0.26	1.06E-01	5.02E-01	9.73E-02	5.49E-01	1.00	4.34E-01	0.26	9.73E-02
Hoffmann	rs734780	15	89564958	PP	T	0.24	1.60E-08	0.06	0.15	0.09	7.55E-01	5.50E-01	5.42E-01	1.00E+00	1.00	9.50E-01	0.09	5.42E-01
Hoffmann	rs9708177	15	90649072	PP	C	-0.27	4.30E-08	-0.05	-0.31	-0.16	8.85E-01	4.51E-01	5.52E-01	1.00E+00	1.00	9.58E-01	-0.16	5.52E-01
Ehret '11	rs2521501	15	91437388	DBP	T	0.36	1.90E-15	0.60	0.35	0.47	1.30E-02	1.33E-01	5.19E-03	6.28E-02	0.39	1.67E-02	0.37	4.47E-17
Ehret '11	rs2521501	15	91437388	SBP	T	0.65	5.20E-19	0.59	0.67	0.63	1.28E-01	7.59E-02	2.00E-02	3.36E-01	0.34	7.26E-02	0.65	3.37E-20
Hoffmann	rs12906962	15	95312071	DBP	T	-0.16	1.30E-10	-0.13	-0.09	-0.11	4.99E-01	6.93E-01	4.43E-01	1.00E+00	1.00	8.69E-01	-0.11	4.43E-01
Hoffmann	rs4984497	15	96635899	DBP	T	0.15	5.40E-09	0.31	-0.16	0.12	1.17E-01	5.16E-01	4.19E-01	5.60E-01	1.00	8.55E-01	0.12	4.19E-01
Hoffmann	rs139491786	16	2086421	SBP	C	1.69	2.60E-07	2.07	2.23	2.12	4.77E-01	5.77E-01	3.66E-01	1.00E+00	1.00	7.77E-01	2.12	3.66E-01
Hoffmann	rs139491786	16	2086421	DBP	C	1.16	1.20E-09	2.18	-0.05	1.40	2.29E-01	9.84E-01	3.37E-01	8.39E-01	1.00	7.81E-01	1.40	3.37E-01
Hoffmann	rs12596053	16	4946794	SBP	A	-0.28	1.50E-12	-0.07	0.44	0.13	7.99E-01	2.04E-01	5.56E-01	1.00E+00	1.00	1.00E+00	0.13	5.56E-01
Hoffmann	rs12596053	16	4946794	DBP	A	-0.16	9.60E-12	-0.12	0.24	0.03	5.12E-01	2.59E-01	8.40E-01	1.00E+00	1.00	1.00E+00	0.03	8.40E-01
Hoffmann	rs3915499	16	15910743	PP	G	0.15	2.30E-08	0.36	0.19	0.28	1.00E-01	4.03E-01	7.88E-02	5.60E-01	1.00	4.40E-01	0.28	7.88E-02

Table S8: Generalization of previously reported loci associated with quantitative BP traits.

Ref	rsID	chr	position	trait	A1	discovery		HCHS/SOL beta			HCHS/SOL p-value			FDR$_g$ r-value			all meta	
						beta	p-value	Main	Carib	All	Main	Carib	All	Main	Carib	All	beta	p-value
Hoffmann	rs9935770	16	21091291	SBP	C	0.22	5.20E-09	0.50	0.54	0.52	7.25E-02	1.08E-01	1.63E-02	5.58E-01	0.66	2.51E-01	0.52	1.63E-02
Hoffmann	rs9935770	16	21091291	DBP	C	0.11	1.90E-06	0.23	0.35	0.28	1.84E-01	9.76E-02	3.79E-02	7.43E-01	0.71	3.09E-01	0.28	3.79E-02
Hoffmann	rs200541	16	24733141	SBP	A	-0.28	8.50E-09	-0.17	-0.61	-0.34	6.56E-01	1.91E-01	2.44E-01	1.00E+00	0.85	6.59E-01	-0.34	2.44E-01
Hoffmann	rs200541	16	24733141	PP	A	-0.17	7.60E-08	-0.06	-0.36	-0.18	8.15E-01	2.26E-01	3.46E-01	1.00E+00	0.83	8.20E-01	-0.18	3.46E-01
Liu	rs11639856	16	24788645	SBP	A	-0.34	1.30E-08	-0.23	-0.44	-0.32	5.35E-01	2.91E-01	2.47E-01	7.14E-01	0.72	4.77E-01	-0.34	7.32E-09
Hoffmann	rs72799341	16	30936743	DBP	G	-0.16	3.70E-09	-0.43	0.00	-0.28	2.15E-02	9.98E-01	6.51E-02	3.57E-01	1.00	3.69E-01	-0.28	6.51E-02
Hoffmann	rs72799341	16	30936743	PP	G	0.13	9.30E-06	0.17	-0.12	0.07	3.74E-01	6.51E-01	6.53E-01	1.00E+00	1.00	1.00E+00	0.07	6.53E-01
Hoffmann	rs56143613	16	56328811	PP	A	0.28	1.50E-09	0.11	0.20	0.15	5.62E-01	4.26E-01	3.47E-01	1.00E+00	1.00	8.20E-01	0.15	3.47E-01
Hoffmann	rs56249585	16	65265702	SBP	C	-0.37	5.20E-09	0.14	-0.20	-0.02	6.60E-01	5.51E-01	9.45E-01	1.00E+00	1.00	1.00E+00	-0.02	9.45E-01
Hoffmann	rs56249585	16	65265702	PP	C	-0.34	6.10E-14	-0.05	0.10	0.02	8.23E-01	6.53E-01	8.84E-01	1.00E+00	1.00	1.00E+00	0.02	8.84E-01
Hoffmann	rs35261357	16	75444572	SBP	C	-0.27	7.90E-12	-0.56	-0.66	-0.60	6.26E-02	5.53E-02	8.04E-03	5.44E-01	0.56	2.51E-01	-0.60	8.04E-03
Hoffmann	rs35261357	16	75444572	PP	C	-0.22	6.40E-17	-0.19	-0.26	-0.22	3.45E-01	2.38E-01	1.38E-01	1.00E+00	0.83	5.06E-01	-0.22	1.38E-01
Hoffmann	rs12928482	16	81513871	SBP	G	0.20	4.10E-06	0.08	0.09	0.08	7.87E-01	8.15E-01	7.20E-01	1.00E+00	1.00	1.00E+00	0.08	7.20E-01
Hoffmann	rs12928482	16	81513871	DBP	G	0.15	1.90E-08	0.22	0.27	0.23	2.17E-01	2.61E-01	9.92E-02	8.32E-01	0.87	4.34E-01	0.23	9.92E-02
Hoffmann	rs7500448	16	83045790	DBP	A	-0.14	6.50E-07	-0.38	-0.18	-0.29	9.62E-02	4.77E-01	8.67E-02	5.49E-01	1.00	4.15E-01	-0.29	8.67E-02
Hoffmann	rs7500448	16	83045790	PP	A	0.30	3.30E-24	0.40	0.18	0.30	9.19E-02	4.92E-01	8.71E-02	5.60E-01	1.00	4.56E-01	0.30	8.71E-02
Hoffmann	rs460105	16	89682006	DBP	T	0.18	3.60E-12	0.32	0.17	0.26	9.62E-02	4.35E-01	7.89E-02	5.49E-01	1.00	4.02E-01	0.26	7.89E-02
Liu	rs1126464	16	89704365	DBP	C	0.24	2.40E-13	0.33	0.39	0.35	6.79E-02	1.45E-01	2.04E-02	3.17E-01	0.51	1.43E-01	0.24	1.04E-16
Hoffmann	rs34457140	17	1353920	SBP	T	-0.18	2.30E-06	-0.37	-0.56	-0.45	1.87E-01	1.04E-01	4.10E-02	8.13E-01	0.66	3.67E-01	-0.45	4.10E-02
Hoffmann	rs34457140	17	1353920	PP	T	-0.16	3.00E-10	-0.07	-0.26	-0.15	7.00E-01	2.45E-01	3.00E-01	1.00E+00	0.83	7.93E-01	-0.15	3.00E-01
Hoffmann	rs9303241	17	1978963	SBP	T	0.20	1.90E-07	0.40	-0.03	0.22	1.85E-01	9.34E-01	3.43E-01	8.13E-01	1.00	7.72E-01	0.22	3.43E-01
Hoffmann	rs9303241	17	1978963	PP	T	0.16	1.00E-09	0.04	0.02	0.03	8.32E-01	9.34E-01	8.32E-01	1.00E+00	1.00	1.00E+00	0.03	8.32E-01
Hoffmann	rs67833703	17	3888437	DBP	C	-0.15	1.10E-08	-0.15	-0.06	-0.11	4.36E-01	7.65E-01	4.37E-01	1.00E+00	1.00	8.69E-01	-0.11	4.37E-01

Table S8: Generalization of previously reported loci associated with quantitative BP traits.

Ref	rsID	chr	position	trait	A1	discovery		HCHS/SOL beta			HCHS/SOL p-value			FDR$_g$ r-value			all meta	
						beta	p-value	Main	Carib	All	Main	Carib	All	Main	Carib	All	beta	p-value
Hoffmann	rs7226020	17	6473828	SBP	T	-0.22	1.10E-07	-0.09	-0.16	-0.12	7.55E-01	6.42E-01	5.95E-01	1.00E+00	1.00	9.46E-01	-0.12	5.95E-01
Hoffmann	rs7226020	17	6473828	PP	T	-0.23	2.10E-17	-0.05	-0.46	-0.22	7.91E-01	3.53E-02	1.24E-01	1.00E+00	0.44	4.96E-01	-0.22	1.24E-01
Hoffmann	rs78378222	17	7571752	DBP	T	-0.64	1.00E-08	-2.96	2.65	-0.56	7.80E-02	1.73E-01	6.59E-01	5.49E-01	1.00	1.00E+00	-0.56	6.59E-01
Hoffmann	rs78378222	17	7571752	PP	T	0.88	4.30E-13	1.43	1.99	1.66	4.13E-01	3.31E-01	2.10E-01	1.00E+00	0.94	6.11E-01	1.66	2.10E-01
Hoffmann	rs35565381	17	16175025	DBP	T	-0.14	3.30E-09	0.37	0.06	0.25	3.39E-02	7.75E-01	6.90E-02	1.00E+00	1.00	1.00E+00	0.25	6.90E-02
Newton-Chen	rs12946454	17	43208121	SBP	T	0.57	1.00E-08	-0.14	0.74	0.26	7.09E-01	6.79E-02	3.43E-01	1.00E+00	0.14	3.43E-01	0.53	1.35E-08
Hoffmann	rs115231027	17	44199290	SBP	T	-0.33	7.60E-07	-0.13	0.13	0.01	8.44E-01	8.32E-01	9.88E-01	1.00E+00	1.00	1.00E+00	0.01	9.88E-01
Hoffmann	rs115231027	17	44199290	PP	T	-0.24	2.90E-08	-0.38	-0.02	-0.19	3.74E-01	9.59E-01	5.18E-01	1.00E+00	1.00	9.48E-01	-0.19	5.18E-01
Ehret ’11	rs17608766	17	45013271	DBP	T	-0.13	1.70E-02	-0.06	0.52	0.24	8.71E-01	1.62E-01	3.78E-01	1.00E+00	1.00	1.00E+00	-0.11	3.04E-02
Ehret ’11	rs17608766	17	45013271	SBP	T	-0.56	1.10E-10	-0.27	0.09	-0.09	6.58E-01	8.83E-01	8.37E-01	7.95E-01	1.00	9.76E-01	-0.54	1.92E-10
Ehret ’11	rs12940887	17	47402807	DBP	T	0.27	2.30E-14	0.17	0.03	0.10	4.71E-01	9.11E-01	5.57E-01	5.89E-01	1.00	6.46E-01	0.26	3.27E-14
Ehret ’11	rs12940887	17	47402807	SBP	T	0.36	1.80E-10	0.13	-0.03	0.05	7.32E-01	9.47E-01	8.46E-01	8.49E-01	1.00	9.76E-01	0.35	3.43E-10
Newton-Chen	rs16948048	17	47440466	DBP	G	0.31	5.00E-09	0.08	0.14	0.11	7.26E-01	5.09E-01	4.76E-01	7.64E-01	1.00	7.14E-01	0.29	9.47E-10
Hoffmann	rs2645466	17	57853214	PP	A	-0.15	3.00E-08	0.03	0.25	0.12	8.95E-01	2.61E-01	3.99E-01	1.00E+00	1.00	1.00E+00	0.12	3.99E-01
Liu	rs8068318	17	59483766	DBP	C	-0.26	3.00E-18	0.02	-0.18	-0.06	9.09E-01	4.02E-01	6.48E-01	1.00E+00	0.80	1.00E+00	-0.25	1.04E-17
Kato	rs2240736	17	59485393	MAP	T	0.35	2.20E-16	0.06	0.40	0.20	7.73E-01	1.06E-01	2.11E-01	1.00E+00	0.16	3.17E-01	0.34	1.52E-18
Hoffmann	rs4295	17	61556298	SBP	C	0.24	1.20E-08	0.08	0.25	0.16	7.82E-01	4.66E-01	4.98E-01	1.00E+00	1.00	8.65E-01	0.16	4.98E-01
Hoffmann	rs4295	17	61556298	DBP	C	0.14	4.20E-08	0.38	0.38	0.38	4.53E-02	7.58E-02	7.83E-03	5.12E-01	0.62	2.00E-01	0.38	7.83E-03
Hoffmann	rs7225219	17	62407559	SBP	T	-0.25	2.50E-09	-0.55	-0.41	-0.50	8.61E-02	3.23E-01	5.02E-02	5.63E-01	0.94	4.02E-01	-0.50	5.02E-02
Hoffmann	rs7225219	17	62407559	PP	T	-0.14	7.80E-07	-0.36	-0.23	-0.31	8.92E-02	3.95E-01	6.35E-02	5.60E-01	1.00	3.93E-01	-0.31	6.35E-02
Hoffmann	rs4788913	17	73950216	SBP	G	-0.28	1.80E-12	-0.73	0.28	-0.30	1.62E-02	4.29E-01	1.92E-01	3.46E-01	1.00	6.58E-01	-0.30	1.92E-01

Table S8: Generalization of previously reported loci associated with quantitative BP traits.

Ref	rsID	chr	position	trait	A1	discovery		HCHS/SOL beta			HCHS/SOL p-value			FDR$_g$ r-value			all meta	
						beta	p-value	Main	Carib	All	Main	Carib	All	Main	Carib	All	beta	p-value
Hoffmann	rs4788913	17	73950216	DBP	G	-0.12	7.50E-07	-0.33	0.42	-0.01	8.33E-02	5.44E-02	9.65E-01	5.49E-01	1.00	1.00E+00	-0.01	9.65E-01
Hoffmann	rs4788913	17	73950216	PP	G	-0.15	8.40E-09	-0.41	-0.15	-0.30	3.78E-02	5.10E-01	4.70E-02	5.44E-01	1.00	3.10E-01	-0.30	4.70E-02
Hoffmann	rs8073626	17	76790279	SBP	C	0.22	1.40E-08	0.25	-0.03	0.14	3.65E-01	9.26E-01	5.22E-01	9.55E-01	1.00	8.78E-01	0.14	5.22E-01
Hoffmann	rs8073626	17	76790279	PP	C	0.12	1.80E-06	0.04	-0.22	-0.07	8.34E-01	3.21E-01	6.35E-01	1.00E+00	1.00	1.00E+00	-0.07	6.35E-01
Levy	rs8096897	18	13438905	SBP	G	-12.87	3.20E-08	-3.34	0.04	-0.87	2.69E-02	9.69E-01	2.67E-01	2.16E-01	1.00	4.01E-01	-2.10	4.81E-03
Hoffmann	rs61735998	18	34289285	PP	G	-0.52	2.40E-08	0.23	1.01	0.61	8.88E-01	5.37E-01	5.94E-01	1.00E+00	1.00	1.00E+00	0.61	5.94E-01
Hoffmann	rs12606620	18	42008097	SBP	G	0.29	2.50E-12	-0.15	0.13	-0.04	6.04E-01	7.18E-01	8.59E-01	1.00E+00	1.00	1.00E+00	-0.04	8.59E-01
Hoffmann	rs12606620	18	42008097	PP	G	0.23	1.50E-16	0.13	0.03	0.09	4.86E-01	9.10E-01	5.41E-01	1.00E+00	1.00	9.50E-01	0.09	5.41E-01
Ehret ’16	rs12958173	18	42141977	SBP	A	0.36	1.43E-13	0.68	-0.24	0.25	4.83E-02	5.12E-01	3.16E-01	3.36E-01	1.00	5.86E-01	0.36	9.59E-14
Ehret ’16	rs12958173	18	42141977	DBP	A	0.18	5.87E-10	0.27	-0.18	0.06	2.15E-01	4.23E-01	7.17E-01	6.58E-01	1.00	1.00E+00	0.17	7.56E-10
Hoffmann	rs2193635	18	43096236	SBP	C	-0.27	1.60E-08	-0.50	-0.19	-0.38	9.14E-02	6.16E-01	1.03E-01	5.76E-01	1.00	6.36E-01	-0.38	1.03E-01
Hoffmann	rs2193635	18	43096236	PP	C	-0.32	9.30E-24	-0.48	-0.27	-0.40	1.41E-02	2.78E-01	9.64E-03	5.44E-01	0.86	2.36E-01	-0.40	9.64E-03
Hoffmann	rs36010659	18	48283949	SBP	T	0.28	3.20E-07	0.29	-0.01	0.17	4.67E-01	9.86E-01	5.89E-01	1.00E+00	1.00	9.44E-01	0.17	5.89E-01
Hoffmann	rs36010659	18	48283949	PP	T	0.25	4.00E-12	0.37	0.14	0.27	1.61E-01	6.45E-01	1.74E-01	7.10E-01	1.00	5.77E-01	0.27	1.74E-01
Hoffmann	rs183335240	18	59096824	DBP	A	2.31	1.30E-08	-5.69	-9.90	-8.42	3.23E-01	1.90E-02	1.34E-02	1.00E+00	1.00	1.00E+00	-8.42	1.34E-02
Liu	rs2302061	19	2226772	PP	C	0.29	2.20E-10	-0.15	0.31	0.06	5.41E-01	2.53E-01	7.43E-01	1.00E+00	0.34	9.90E-01	0.27	1.30E-08
Kato	rs740406	19	2232221	PP	A	0.55	3.10E-15	-0.00	-0.28	-0.13	9.87E-01	3.64E-01	5.36E-01	1.00E+00	1.00	1.00E+00	0.48	4.69E-13
Liu	rs7248104	19	7224431	PP	A	-0.20	2.60E-10	0.18	-0.30	-0.02	3.33E-01	1.71E-01	8.87E-01	1.00E+00	0.34	1.00E+00	-0.19	5.55E-11
Ehret ’16	rs4247374	19	7252756	SBP	T	-0.59	1.23E-18	-0.69	-0.08	-0.38	2.47E-01	8.90E-01	3.63E-01	5.35E-01	1.00	5.91E-01	-0.59	8.90E-19
Ehret ’16	rs4247374	19	7252756	DBP	T	-0.39	2.08E-22	-0.42	-0.36	-0.39	2.53E-01	3.20E-01	1.33E-01	6.58E-01	0.69	4.71E-01	-0.39	7.36E-23
Hoffmann	rs10427021	19	7259346	SBP	T	0.51	9.50E-19	1.11	-0.15	0.41	3.24E-02	7.43E-01	2.36E-01	4.17E-01	1.00	6.58E-01	0.41	2.36E-01
Hoffmann	rs10427021	19	7259346	DBP	T	0.34	4.50E-22	0.63	0.31	0.45	5.36E-02	2.88E-01	3.83E-02	5.12E-01	0.90	3.09E-01	0.45	3.83E-02
Hoffmann	rs200688233	19	10372360	PP	G	-0.31	3.90E-08	-0.06	0.24	0.09	8.62E-01	4.85E-01	7.01E-01	1.00E+00	1.00	1.00E+00	0.09	7.01E-01

Table S8: Generalization of previously reported loci associated with quantitative BP traits.

Ref	rsID	chr	position	trait	A1	discovery		HCHS/SOL beta			HCHS/SOL p-value			FDR$_g$ r-value			all meta	
						beta	p-value	Main	Carib	All	Main	Carib	All	Main	Carib	All	beta	p-value
Liu	rs167479	19	11526765	DBP	T	-0.30	4.20E-28	0.26	-0.18	0.07	2.51E-01	4.89E-01	6.83E-01	1.00E+00	0.86	1.00E+00	-0.29	1.31E-22
Hoffmann	rs167479	19	11526765	SBP	G	0.41	1.60E-21	-0.60	0.75	-0.02	1.05E-01	7.64E-02	9.50E-01	1.00E+00	0.65	1.00E+00	-0.02	9.50E-01
Hoffmann	rs167479	19	11526765	DBP	G	0.25	4.30E-22	-0.26	0.18	-0.07	2.51E-01	4.89E-01	6.83E-01	1.00E+00	1.00	1.00E+00	-0.07	6.83E-01
Hoffmann	rs167479	19	11526765	PP	G	0.18	3.20E-08	-0.32	0.57	0.07	1.87E-01	3.56E-02	6.90E-01	1.00E+00	0.44	1.00E+00	0.07	6.90E-01
Hoffmann	rs10418305	19	15278808	PP	C	-0.33	3.50E-16	-0.40	-0.47	-0.42	5.91E-02	1.40E-01	1.73E-02	5.44E-01	0.74	2.86E-01	-0.42	1.73E-02
Hoffmann	rs4808569	19	17218970	SBP	C	0.23	1.30E-06	0.51	0.40	0.46	2.18E-01	4.12E-01	1.45E-01	8.26E-01	1.00	6.36E-01	0.46	1.45E-01
Hoffmann	rs4808569	19	17218970	DBP	C	0.16	4.50E-08	0.24	0.19	0.22	3.48E-01	5.31E-01	2.66E-01	9.87E-01	1.00	6.68E-01	0.22	2.66E-01
Hoffmann	rs8103992	19	19665643	PP	A	0.20	9.50E-10	-0.35	0.29	-0.02	2.00E-01	2.67E-01	9.15E-01	1.00E+00	0.85	1.00E+00	-0.02	9.15E-01
Hoffmann	rs34331990	19	30321561	DBP	T	-0.15	2.80E-10	-0.22	0.01	-0.12	2.58E-01	9.69E-01	4.13E-01	8.98E-01	1.00	8.55E-01	-0.12	4.13E-01
Hoffmann	rs8105753	19	31927547	SBP	A	0.25	3.60E-10	-0.05	-0.04	-0.05	8.56E-01	9.12E-01	8.32E-01	1.00E+00	1.00	1.00E+00	-0.05	8.32E-01
Hoffmann	rs8105753	19	31927547	DBP	A	0.14	1.90E-08	-0.19	0.11	-0.08	2.69E-01	6.32E-01	5.65E-01	1.00E+00	1.00	1.00E+00	-0.08	5.65E-01
Hoffmann	rs4803457	19	41861359	PP	T	0.15	1.50E-08	-0.13	0.03	-0.06	4.76E-01	8.81E-01	6.50E-01	1.00E+00	1.00	1.00E+00	-0.06	6.50E-01
Hoffmann		20	10573001	PP	C	0.37	1.10E-09	0.12	-0.82	-0.48	8.08E-01	2.88E-02	1.13E-01	1.00E+00	1.00	1.00E+00	0.34	1.52E-08
HMG	rs1887320	20	10965998	DBP	A	0.43	2.13E-08	0.52	0.64	0.57	1.31E-02	2.62E-03	1.16E-04	6.23E-02	0.03	1.11E-03	0.46	5.44E-11
HMG	rs1887320	20	10965998	SBP	A	0.78	1.48E-08	1.26	0.78	1.02	1.53E-04	2.27E-02	1.94E-05	2.90E-03	0.14	1.48E-03	0.84	3.25E-12
Hoffmann	rs2104574	20	10968891	PP	C	-0.13	2.20E-05	-0.72	-0.16	-0.43	7.02E-03	5.38E-01	2.01E-02	5.44E-01	1.00	3.10E-01	-0.14	5.04E-06
Hoffmann	rs2104574	20	10968891	DBP	C	-0.23	4.00E-16	-0.62	-0.30	-0.46	1.42E-02	2.38E-01	1.08E-02	3.26E-01	0.87	2.07E-01	-0.24	3.29E-17
Ehret ’11	rs1327235	20	10969030	DBP	G	0.30	1.40E-15	0.54	0.66	0.60	9.72E-03	1.77E-03	6.07E-05	5.63E-02	0.01	6.85E-04	0.32	2.62E-18
Ehret ’11	rs1327235	20	10969030	SBP	G	0.34	1.90E-08	1.26	0.80	1.03	1.42E-04	1.92E-02	1.50E-05	4.80E-03	0.14	4.80E-03	0.38	7.47E-11
Hoffmann	rs3790227	20	19469002	PP	C	-0.22	3.00E-15	-0.40	-0.66	-0.50	3.09E-02	3.47E-03	4.52E-04	5.44E-01	0.23	8.95E-02	-0.50	4.52E-04
Hoffmann	rs369386096	20	23502129	DBP	C	-9.79	3.60E-08	-42.97	-11.94	-12.61	6.38E-01	3.79E-01	3.48E-01	1.00E+00	0.95	7.82E-01	-12.61	3.48E-01
Hoffmann	rs6060114	20	30169673	SBP	T	0.26	1.70E-06	0.25	0.35	0.30	5.33E-01	3.56E-01	2.76E-01	1.00E+00	0.95	6.91E-01	0.30	2.76E-01
Hoffmann	rs6060114	20	30169673	DBP	T	0.24	6.50E-13	0.17	0.32	0.25	4.99E-01	1.73E-01	1.51E-01	1.00E+00	0.83	5.47E-01	0.25	1.51E-01

Table S8: Generalization of previously reported loci associated with quantitative BP traits.

Ref	rsID	chr	position	trait	A1	discovery		HCHS/SOL beta			HCHS/SOL p-value			FDR$_g$ r-value			all meta	
						beta	p-value	Main	Carib	All	Main	Carib	All	Main	Carib	All	beta	p-value
Hoffmann	rs2424908	20	31360383	PP	C	-0.19	4.50E-10	0.04	-0.48	-0.16	8.24E-01	4.36E-02	2.91E-01	1.00E+00	0.44	7.77E-01	-0.16	2.91E-01
Hoffmann	rs6129880	20	40251829	SBP	T	0.26	2.10E-08	0.44	0.27	0.38	1.26E-01	4.65E-01	1.01E-01	6.50E-01	1.00	6.36E-01	0.38	1.01E-01
Hoffmann	rs6129880	20	40251829	DBP	T	0.17	2.10E-09	0.08	0.04	0.07	6.38E-01	8.48E-01	6.32E-01	1.00E+00	1.00	1.00E+00	0.07	6.32E-01
Hoffmann	rs6031435	20	42797358	SBP	A	-0.21	2.40E-08	-0.88	-0.14	-0.55	4.52E-03	6.76E-01	1.81E-02	3.46E-01	1.00	2.56E-01	-0.55	1.81E-02
Hoffmann	rs6031435	20	42797358	PP	A	-0.17	2.80E-11	-0.54	-0.10	-0.34	7.82E-03	6.45E-01	2.46E-02	5.16E-01	1.00	3.10E-01	-0.34	2.46E-02
Hoffmann	rs6019378	20	47309716	DBP	C	0.16	1.80E-11	0.17	-0.07	0.07	3.34E-01	7.22E-01	6.06E-01	9.65E-01	1.00	1.00E+00	0.07	6.06E-01
Ehret '11	rs6015450	20	57751117	DBP	G	0.56	5.60E-23	0.26	-0.19	0.00	4.88E-01	5.61E-01	9.90E-01	5.89E-01	1.00	1.00E+00	0.53	6.62E-22
Ehret '11	rs6015450	20	57751117	SBP	G	0.90	3.90E-23	0.79	-0.70	-0.07	1.87E-01	1.73E-01	8.68E-01	3.82E-01	1.00	1.00E+00	0.85	6.85E-22
Hoffmann	rs6090040	20	62692060	SBP	A	0.27	1.40E-09	0.36	0.86	0.59	2.39E-01	1.06E-02	1.01E-02	8.30E-01	0.45	2.51E-01	0.59	1.01E-02
Hoffmann	rs6090040	20	62692060	PP	A	0.15	2.60E-07	-0.05	0.27	0.10	8.21E-01	2.09E-01	4.96E-01	1.00E+00	0.83	9.48E-01	0.10	4.96E-01
Hoffmann	rs13050325	21	16343812	SBP	A	-0.26	5.50E-09	-0.52	-0.54	-0.53	7.55E-02	1.71E-01	2.49E-02	5.58E-01	0.82	3.02E-01	-0.53	2.49E-02
Hoffmann	rs13050325	21	16343812	PP	A	-0.15	6.90E-07	-0.17	-0.39	-0.25	3.91E-01	1.30E-01	1.10E-01	1.00E+00	0.73	4.96E-01	-0.25	1.10E-01
Hoffmann	rs57448815	21	30123533	PP	A	-0.23	6.00E-09	0.20	-0.92	-0.38	4.60E-01	4.30E-04	4.53E-02	1.00E+00	0.05	3.10E-01	-0.38	4.53E-02
Hoffmann	rs11701033	21	33788341	SBP	C	-0.25	3.20E-07	-0.58	0.27	-0.30	6.72E-02	5.43E-01	2.54E-01	5.44E-01	1.00	6.61E-01	-0.30	2.54E-01
Hoffmann	rs11701033	21	33788341	PP	C	-0.18	5.70E-08	-0.29	0.17	-0.13	1.66E-01	5.48E-01	4.40E-01	7.15E-01	1.00	9.04E-01	-0.13	4.40E-01
Hoffmann	rs117870289	21	39983448	DBP	C	0.54	1.00E-06	-0.55	0.73	0.11	7.80E-01	6.99E-01	9.35E-01	1.00E+00	1.00	1.00E+00	0.11	9.35E-01
Hoffmann	rs117870289	21	39983448	PP	C	-0.73	2.40E-09	-1.79	-1.43	-1.61	3.78E-01	4.73E-01	2.59E-01	1.00E+00	1.00	7.12E-01	-1.61	2.59E-01
Hoffmann	rs112204826	21	44721027	SBP	C	-0.60	5.00E-07	-0.70	-0.32	-0.51	5.15E-01	7.62E-01	5.00E-01	1.00E+00	1.00	8.65E-01	-0.51	5.00E-01
Hoffmann	rs112204826	21	44721027	DBP	C	-0.40	2.70E-08	-0.98	-0.22	-0.59	1.43E-01	7.34E-01	2.05E-01	6.21E-01	1.00	6.02E-01	-0.59	2.05E-01
Ehret '16	rs12627651	21	44760603	SBP	A	0.39	2.69E-14	-0.30	0.57	0.04	3.45E-01	1.47E-01	8.70E-01	1.00E+00	0.64	1.00E+00	0.38	7.13E-14
Ehret '16	rs12627651	21	44760603	DBP	A	0.20	1.36E-11	-0.14	-0.15	-0.14	4.78E-01	5.29E-01	3.46E-01	1.00E+00	1.00	1.00E+00	0.19	1.05E-10
Hoffmann	rs8139817	22	18468369	DBP	A	-0.14	3.80E-08	0.12	-0.21	-0.05	6.00E-01	3.38E-01	7.43E-01	1.00E+00	0.92	1.00E+00	-0.05	7.43E-01
Hoffmann	rs2012714	22	19977647	SBP	C	-0.22	1.30E-06	-0.08	-0.16	-0.11	7.82E-01	6.48E-01	6.16E-01	1.00E+00	1.00	9.69E-01	-0.11	6.16E-01

Table S8: Generalization of previously reported loci associated with quantitative BP traits.

Ref	rsID	chr	position	trait	A1	discovery		HCHS/SOL beta			HCHS/SOL p-value			FDR$_g$ r-value			all meta	
						beta	p-value	Main	Carib	All	Main	Carib	All	Main	Carib	All	beta	p-value
Hoffmann	rs2012714	22	19977647	PP	C	-0.18	2.80E-09	0.08	-0.28	-0.07	6.81E-01	2.25E-01	6.53E-01	1.00E+00	0.83	1.00E+00	-0.07	6.53E-01
Hoffmann	rs34887403	22	29151150	SBP	G	-0.30	3.10E-08	0.69	-0.34	0.17	1.77E-01	5.02E-01	6.35E-01	1.00E+00	1.00	1.00E+00	0.17	6.35E-01
Liu	rs4823006	22	29451671	SBP	G	-0.26	7.90E-09	-0.36	0.04	-0.20	1.96E-01	9.06E-01	3.58E-01	3.93E-01	1.00	4.77E-01	-0.26	1.34E-07
Hoffmann	rs12485003	22	40635276	PP	G	-0.33	4.60E-13	-0.08	-0.51	-0.17	6.78E-01	1.80E-01	3.30E-01	1.00E+00	0.82	8.09E-01	-0.17	3.30E-01

5 Replication studies

5.1 COGENT

The Continental Origins and Genetic Epidemiology Network Blood Pressure (COGENT-BP) is a consortium of African American and African studies that was brought together with the goal of identifying genetic loci that account for the increased risk of hypertension in populations of African descent (Franceschini et al., 2013). Standardized protocols for phenotype harmonization and for statistical analyses have been previously described (Franceschini et al., 2013). For the replication of HCHS/SOL findings, we used data from 31,968 individuals from 19 African American studies and two Nigeria cohorts, which have imputed genotypes from Phase 1 integrated (March 2012 release) multi-ethnic reference panel from the 1000 Genomes Project (1KG) Consortium 1 (1000 Genomes Project Consortium, 2012). Participating studies:

5.2 WHI

WHI is a long-term national health study that focuses on strategies for preventing common diseases such as heart disease, cancer and fracture in postmenopausal women. A total of 161,838 women aged 50-79 years old were recruited from 40 clinical centers in the US between 1993 and 1998. WHI consists of an observational study, two clinical trials of postmenopausal hormone therapy (estrogen alone or estrogen plus progestin), a calcium and vitamin D supplement trial, and a dietary modification trial (Hays et al., 2003). Demographic data, medical history and anthropometric measures were obtained at a baseline clinical visit. BP was measured by certified staff using standardized procedures and instruments (Hsia et al., 2007). Two BP measures were recorded after 5 minutes rest using a mercury sphygmomanometer. Diastolic BP was taken from the phase V Korotkoff measures. The average of the two measurements, obtained 30 seconds apart, was used in analyses. Women were asked to bring all of their current prescription and over-the- counter medications to each visit. The WHI European ancestry samples included two studies: GARNET (N=4,279, genotyped using the Illumina HumanOmni1-Quad v1-0 B) and WHIMS (N=5,478, HumanOmniExpressExome-8v1_B), already imputed to 1000G reference panels.

5.3 UK Biobank

The UK Biobank (UKB) is a prospective cohort study including ~500,000 male and female consenting volunteers aged 40-69 years ascertained through NHS registers (Allen et al., 2012). Data includes extensive baseline phenotypic measurements, stored biological samples (Elliott and Peakman, 2008), and follow-up by electronic health record linkage (Sudlow et al., 2015). Two clinic blood pressure measurements are taken seated after two minutes rest using an appropriate cuff and an Omron HEM-7015IT digital blood pressure monitor. Interim data are available for 152,249 UKB participants: ~100k individuals from UKB genotyped at ~800,000 single nucleotide variants (SNVs) with a custom Affymetrix UK Biobank Axiom Array chip and ~50k individuals genotyped with a custom Affymetrix UK BiLEVE Axiom Array chip from the UK BiLEVE study (Wain et al., 2015), a subset of UKB. The two arrays overlap with over 95% of SNVs content in common. SNVs were imputed centrally by UKB using a merged UK10K sequencing +1000G imputation reference panel, yielding a total of ~73 million autosomal genetic variants available for analysis (Huang et al., 2015). Initial Quality Control (QC) of the genetic data was performed centrally by UK Biobank (Biobank, 2015). Further details are available at the UK Biobank website: www.ukbiobank.ac.uk. As further QC, we excluded discordant SNVs and samples with QC failures, gender discordance and high heterozygosity/missingness. We further restricted our data to a subset of individuals of European ancestry, by applying kmeans clustering to the Principal Component Analysis (PCA) data provided, with a total of N=145,315 Europeans remaining. We used the provided kinship data to exclude 1st and 2nd degree relatives, with N=141,647 unrelated individuals remaining. Finally we restricted our data to non-pregnant individuals with two automated BP measurements available, resulting in a maximum of N=140,886 unrelated individuals of European ancestry for analysis. After calculating the mean SBP and DBP values from the two BP measurements, we adjusted for medication use by adding 15 and 10 mmHg to SBP and DBP, respectively, for individuals reported to be taking BP-lowering medication (21.4% of individuals). PP is calculated as SBP - DBP. We carried out GWAS analyses of (untransformed) medication-adjusted systolic (SBP), diastolic (DBP) and pulse pressure (PP) traits using single-variant linear regression in dosage format using SNPTEST software (Marchini et al., 2007) under an additive genetic model. Each analysis included the following covariates: sex, age, age^2, body

mass index, top ten PCs and a binary indicator variable for UK Biobank vs UK BiLEVE to adjust for the different genotyping chips. Further details of the QC and GWAS analyses we performed have been described previously (Warren et al., 2017).

5.3.1 UK Biobank replication acknowledgements

Part of this work used computing resources provided by the MRC- funded UK MEDical Bioinformatics partnership programme (UK MED-BIO) (MR/L01632X/1).

This research has been conducted using the UK Biobank Resource under Application Number 236.

trait	rsID	Chr	position	A1	A2	HCHS/SOL			WHI			Biobank			EA meta	
						EAF	beta	*p*-value	EAF	beta	*p*-value	EAF	beta	*p*-value	beta	*p*-value
SBP	rs143503553	5	159593663	G	C	0.01	7.99	5.94E-08	0.01	-0.18	9.40E-01	0.01	0.44	3.51E-01	0.42	3.70E-01
SBP	rs9366626*	6	25684953	G	A	0.55	1.18	8.75E-08	0.6	-0.35	1.10E-01	0.61	-0.14	4.02E-02	-0.16	1.50E-02
MAP	rs9366626*	6	25684953	G	A	0.55	0.79	3.04E-07	0.6	-0.31	2.00E-02	NA	NA	NA	-0.31	2.25E-02
DBP		10	84135292	CA	C	0.3	0.94	7.05E-09	NA	NA	NA	0.16	0.06	3.15E-01	0.06	3.15E-01
MAP	rs7909484*	10	84206002	T	C	0.4	0.68	1.50E-05	0.77	0.15	3.40E-01	NA	NA	NA	0.15	3.37E-01
DBP	rs7909484*	10	84206002	T	C	0.4	0.6	1.55E-05	0.26	0.1	4.20E-01	0.22	-0.01	8.70E-01	0.01	8.97E-01
SBP	rs73156692	12	101608695	A	G	0.16	1.65	5.44E-08	0.24	0.52	4.00E-02	0.25	0.18	2.84E-02	0.21	7.05E-03
PP	rs117386367	17	53098512	A	G	0.01	5.01	7.61E-08	0.01	-0.14	9.50E-01	<0.01	-0.1	8.10E-01	-0.1	8.05E-01

Table S9: Replication results in the two studies of European ancestry (WHI and Biobank), and their meta-analysis. SNPs marked with an asterisk are secondary variants (not the lead HCHS/SOL SNPs in their region).

5.4 1982 Pelotas Birth Cohort Study

Characteristics of the genotyped participants from the 1982 Pelotas Birth Cohort Study (Victora and Barros, 2006; Horta et al., 2015) are provided in Table S10. Genotyping was performed on an Illumina HumanOmni2.5-8v1 array. For quality control, SNPs excluded if their call rate was $< 95\%$, their Hardy-Weinberg $P < 1 \times 10^{-7}$ or were monomorphic. Samples were excluded if there were sex mismatches (heterozygosity threshold 0.02), heterozygosity rate outside the range of median$\pm 1.5 \times$IQR, missingness $>$ 3% and cryptic relatedness (kinship> 0.1, as described elsewhere). Pre-phasing was done using SHAPEIT (Delaneau et al., 2013) and imputation using IMPUTE2 (Howie et al., 2009), with reference panel 1000 Genomes (1000 Genomes Project Consortium, 2012) Phase I integrated haplotypes - December 2013 release.

Characteristic	Measure
n	2764
Mean age (SD)	30.2 (0.3)
female sex	1422 (51.4%)
Mean BMI	27.0 (5.6)
Hypertension	329 (11.9%)
Mean SBP (SD)	121.3 (13.7)
Mean DBP (SD)	75.6 (9.4)
Mean MAP (SD)	60.4 (9.6)
Mean PP (SD)	45.7 (9.1)

Table S10: Sample characteristics of the Pelotas birth cohort study.

6 Reproducibility

All GWAS were performed and tracked by the Integrated Computing and Tracking (ICT) system (Stilp et al., 2017) at the HCHS/SOL genetic analysis center (GAC). The unique analysis IDs are provided in

Table S11.

Trait	Analysis ID - main analyses	Analysis ID - conditional analyses
DBP	s536935	s949424
SBP	s485005	s358622
MAP	s266973	s820826
PP	s246600	s174992
HT (stratified)	s930006	
HT (overall)	855289	

Table S11: Analysis IDs of the performed GWAS in the HCHS/SOL GAC tracking database. Conditional analysis used SNPs from multiple loci (SNPs rs2240736, rs1902859, rs11953630, and rs1799945) as additional covariates in the regression model. HT has different analysis ID for the overall analysis since stratified analyses could not be combined in meta-analysis when the outcome is binary and there are related individuals between the strata.

References

1000 Genomes Project Consortium (2012). An integrated map of genetic variation from 1,092 human genomes. *Nature*, **491** 56–65.

Allen, N., Sudlow, C., Downey, P., Peakman, T., Danesh, J., Elliott, P., Gallacher, J., Green, J., Matthews, P. and Pell, e. a., Jill (2012). UK Biobank: Current status and what it means for epidemiology. *Health Policy and Technology*, **1** 123–126.

Biobank, U. (2015). Genotyping and quality control of UK Biobank, a large-scale, extensively phenotyped prospective resource. *Available at biobank. ctsu. ox. ac. uk/crystal/docs/genotyping_qc. pdf. Accessed April*, **1** 2016.

Browning, S., Grinde, K., Plantinga, A., Gogarten, S., Stilp, A. and Kaplan, e. a., RC (2016). Local Ancestry Inference in a Large US-Based Hispanic/Latino Study: Hispanic Community Health Study/Study of Latinos (HCHS/SOL). *G3: Genes— Genomes— Genetics*, **6** 1525–1534.

Delaneau, O., Zagury, J.-F. and Marchini, J. (2013). Improved whole-chromosome phasing for disease and population genetic studies. *Nature methods*, **10** 5–6.

Elliott, P. and Peakman, T. C. (2008). The UK Biobank sample handling and storage protocol for the collection, processing and archiving of human blood and urine. *International Journal of Epidemiology*, **37** 234–244.

Franceschini, N., Fox, E., Zhang, Z., Edwards, T. L., Nalls, M. A., Sung, Y. et al. (2013). Genome-wide association analysis of blood-pressure traits in African-ancestry individuals reveals common associated genes in African and non-African populations. *The American Journal of Human Genetics*, **93** 545–554.

Gibbs, R. A., Belmont, J. W., Hardenbol, P., Willis, T. D., Yu, F., Yang, H., Ch'ang, L.-Y., Huang, W., Liu, B., Shen, Y. et al. (2003). The international HapMap project. *Nature*, **426** 789–796.

Hays, J., Hunt, J., Hubbell, F., Anderson, G., Limacher, M., Allen, C. and Rossouw, J. (2003). The Women's Health Initiative recruitment methods and results. *Annals of epidemiology*, **13** S18–S77.

Horta, B., Gigante, D., Gonçalves, H., dos Santos Motta, J., de Mola, C. and Oliveira, e. a., IO (2015). Cohort profile update: the 1982 Pelotas (Brazil) birth cohort study. *International journal of epidemiology*, **44** 441–441e.

Howie, B., Donnelly, P. and Marchini, J. (2009). A flexible and accurate genotype imputation method for the next generation of genome-wide association studies. *PLoS Genet*, **5** e1000529.

Hsia, J., Margolis, K., Eaton, C., Wenger, N., Allison, M. and Wu, e. a., L (2007). Prehypertension and cardiovascular disease risk in the Women?s Health Initiative. *Circulation*, **115** 855–860.

Huang, J., Howie, B., McCarthy, S., Memari, Y., Walter, K., Min, J. L., Danecek, P., Malerba, G., Trabetti, E. and Zheng, e. a., Hou-Feng (2015). Improved imputation of low-frequency and rare variants using the UK10K haplotype reference panel. *Nature communications*, **6** 8111.

Liu, C., Kraja, A., Smith, J., Brody, J., Franceschini, N. and Bis, e. a., JC (2016). Meta-analysis identifies common and rare variants influencing blood pressure and overlapping with metabolic trait loci. *Nat Genet*, **48** 1162–1170.

Marchini, J., Howie, B., Myers, S., McVean, G. and Donnelly, P. (2007). A new multipoint method for genome-wide association studies by imputation of genotypes. *Nature genetics*, **39** 906–913.

Stilp, A. M., Gogarten, S. M., Laurie, C. C. and Sofer, T. (2017). Integrated Computing And Tracking System For Centralized High-Throughput Genetic Analysis: A Case Study. *bioRxiv* 137596.

Sudlow, C., Gallacher, J., Allen, N., Beral, V., Burton, P., Danesh, J., Downey, P., Elliott, P., Green, J. and Landray, e. a., Martin (2015). UK biobank: an open access resource for identifying the causes of a wide range of complex diseases of middle and old age. *PLoS medicine*, **12** e1001779.

Victora, C. G. and Barros, F. C. (2006). Cohort profile: the 1982 Pelotas (Brazil) birth cohort study. *International journal of epidemiology*, **35** 237–242.

Wain, L. V., Shrine, N., Miller, S., Jackson, V. E., Ntalla, I., Artigas, M. S., Billington, C. K., Kheirallah, A. K., Allen, R. and Cook, e. a., James P (2015). Novel insights into the genetics of smoking behaviour, lung function, and chronic obstructive pulmonary disease (UK BiLEVE): a genetic association study in UK Biobank. *The Lancet Respiratory Medicine*, **3** 769–781.

Warren, H. R., Evangelou, E., Cabrera, C. P., Gao, H., Ren, M., Mifsud, B., Ntalla, I., Surendran, P., Liu, C. and Cook, e. a., James P (2017). Genome-wide association analysis identifies novel blood pressure loci and offers biological insights into cardiovascular risk. *Nat Genet*, **49** 403–415.

Zhang, Q., Browning, B. and Browning, S. (2016). ASAFE: ancestry-specific allele frequency estimation. *Bioinformatics*, **32 (14)** 2227–2229.
